# Participatory approaches in the development of health interventions for migrants: a systematic review

**DOI:** 10.1136/bmjopen-2021-053678

**Published:** 2021-10-25

**Authors:** Kieran Rustage, Alison Crawshaw, Saliha Majeed-Hajaj, Anna Deal, Laura Nellums, Yusuf Ciftci, Sebastian S Fuller, Lucy Goldsmith, Jon S Friedland, Sally Hargreaves

**Affiliations:** 1 Migrant Health Research Group, Institute for Infection and Immunity, St George’s University of London, London, UK; 2 School of Georgraphy, Queen Mary University of London, London, UK; 3 Department of Public Health and Policy, London School of Hygiene and Tropical Medicine, London, UK; 4 Division of Epidemiology and Public Health, University of Nottingham, Nottingham, UK; 5 Doctors of the World, London, UK; 6 Population Health Research Institute, St George’s University of London, London, UK

**Keywords:** public health, statistics & research methods, education & training (see medical education & training)

## Abstract

**Objective:**

Analysis of participatory approaches to developing health interventions for migrants and how approaches embody core participatory principles of inclusivity and democracy.

**Design:**

A systematic review of original articles. Electronic searches within the databases MEDLINE, Embase, Global Health and PsychINFO (from inception—November 2020).

**Eligibility criteria for study selection:**

Original peer-reviewed articles reporting research to develop and implement a health intervention for migrants, incorporating participatory approaches. We defined migrants as foreign-born individuals. Only articles reporting the full research cycle (inception, design, implementation, analysis, evaluation, dissemination) were included.

**Data extraction:**

We extracted information related to who was involved in research (migrants or other non-academic stakeholders), the research stage at which they were involved (inception, design, implementation, analysis, evaluation, dissemination), the method of their involvement and how this aligned with the core principles of participatory research—categorising studies as exhibiting active or pseudo (including proxy and indirect) participation.

**Results:**

1793 publications were screened, of which 28 were included in our analysis. We found substantial variation in the application of participatory approaches in designing health interventions targeting migrants: across 168 individual research stages analysed across the 28 studies, we recorded 46 instances of active participation of migrants, 30 instances of proxy participation and 24 instances of indirect participation. All studies involved non-academic stakeholders in at least one stage of the research, only two studies exhibited evidence of active participation of migrants across all research stages. Evidence is limited due to the variability of terms and approaches used.

**Conclusions:**

Important shortfalls in the meaningful inclusion of migrants in developing health interventions exist, suggesting a more rigorous and standardised approach is warranted to better define and deliver participatory research and improve quality.

**Registration:**

This review followed Preferred Reporting Items for Systematic Review and Meta-Analysis guidelines and is registered on the Open Science Framework (osf.io/2bnz5).

**Strengths and limitations of this study**

- This systematic review represents a robust and novel assessment of the application of participatory approaches and principles towards research into health interventions involving migrants.
- This review casts a critical lens over the relationship between how participatory approaches are applied and how participatory principles such as inclusivity and democracy are embodied.
- Due to the varied and interchangeable use of participatory research terms, the categorisations and definitions we use could be interpreted differently by others.
- This review is limited by the lack of clear and consistent reporting participatory methods used, suggesting that guidelines must be developed and more consistently adopted to improve transparency in all participatory research.
- This review does not address possible associations between participatory methods and final health or research outcomes, which should be better considered in future research.

## Introduction

Considerable emphasis is now being placed on ensuring patient and public engagement in health research, including striving for greater involvement of marginalised groups such as migrants and ethnic minorities.^1 2^ However, whether this is effectively and meaningfully done in practice to ensure truly patient-centred research has yet to be fully elucidated. Participatory research represents a distinct research paradigm in which research is done collaboratively with the individuals whose lived experiences and actions are the subject of study, as active partners who share power and influence over research processes and outcomes.^3–6^

Two fundamental principles of participatory research that underpin the ability for stakeholders to effectively co-operate and share power are those of inclusivity and democracy, particularly in relation to those directly affected by the research in question.^3^ That is to say, has the research included the individuals the research would otherwise be about, and have these individuals, during their inclusion, had influence or power over research decisions on par with the research professionals? Included under the umbrella of participatory research approaches are more specific methodologies, which look to uphold these principles, including: community-based participatory research (CBPR);^7^ action research;^8^ some patient & public involvement 9 as well as broader derivatives such as community-based collaborative action research. Participatory research holds the potential to bridge the gap between public health research and practice, creating a context in which patients and the public have meaningful influence over research decisions, increasing the relevance and impact of research outcomes to their own lives.^10^

Participatory research is likely to be particularly powerful when working with underserved and marginalised groups such as migrants, where traditional research has frequently failed to provide an appreciable health benefit. While a heterogeneous group, comprising a multitude of cultures, ethnicities and sociocultural circumstances, many migrants can find themselves in vulnerable situations, marginalised by health systems^11 12^ and society alike.^13 14^ There is a growing consensus around the need for academics and health systems to become more responsive to, and inclusive of refugee and migrant health concerns.^15^ Indeed, limited community engagement in public health interventions has already been shown to be effective when working with marginalised groups around a range of health outcomes and can provide benefits to participants themselves, such as in improving health behaviours and participant self-efficacy.^16^ However, the ultimate goal is to conduct participatory research with migrants as a matter of routine, so that research is better centred around and grounded in the needs of migrant communities.

Despite the potential of participatory research, there are varied interpretations as to how to apply such approaches. A review of peer models in participatory research, in which partnerships with ‘insiders’ are established reveal a norm in which practices and terms are interchangeable and inconsistently applied.^17^ Challenges exist in deciding who should be involved, and whether involvement should extend beyond the target group (in this case, migrants), to other non-academic stakeholder groups such as community organisations and professionals.^3 17^ There are also differing interpretations of the degree of participation required of individuals for research to be considered participatory rather than tokenistic, though it is suggested that unless involved individuals are partners or coresearchers throughout the entirety of a project, the work cannot be participatory.^3 18^ Overall, it is widely agreed that quality participation is characterised by non-academic stakeholders having opportunities to engage with, make decisions about and perform leadership roles around such research,^5^ empowering the public at the highest level and asserting their right to be involved in decision-making and to influence outcomes. Understanding the different approaches to participatory research and whether the core principles of participatory research are upheld is crucially important if good practice is to be identified.

We, therefore, did a systematic review to analyse participatory approaches in the development of health interventions for migrants, through use of a framework, which relates categories of participation to core principles of participation (inclusivity and democracy), and collates evidence of the benefits of using a participatory approach to research, and of the challenges of using a participatory approach to research.

## Methods

We did a systematic review, following Preferred Reporting Items for Systematic Review and Meta-Analysis guidelines, which is registered on the Open Science Framework. The primary aim of this systematic review was to analyse the use of participatory approaches to develop health interventions targeting migrants as the intended beneficiaries. Specifically, we established a framework of categories of participation, which related the data we extracted to participatory principles of inclusivity and democracy. Our secondary aims were to describe the challenges and benefits of using participatory approaches experienced in the research process.

### Inclusion and exclusion criteria

We included peer-reviewed primary-research reporting on health research into interventions aimed at benefitting migrant populations that described using a participatory approach across the whole research process. Research that purported to use a participatory research approach through descriptors in their introduction and methods, or which used a recognised participatory approach such as CBPR, action research or community-based collaborative action research and specifically targeted migrants was included in the review. We defined migrants as foreign-born individuals and considered a health intervention to be any initiative, tool or programme that looked to improve health outcomes, including those related to mental health and health literacy.

Studies were excluded if they did not report on all stages of research into the health intervention: inception, design, implementation, analysis, evaluation, dissemination.

As such, publications presenting interim results of studies which had not completed the full research cycle as well as studies specifically focusing on only codesigning interventions were excluded. We took this approach so as not to unfairly penalise ongoing research in our analysis, nor codesigned research; we consider codesign to be one component of the broader participatory research paradigm and were most interested in how approaches manifest across the entirety of a research cycle. Studies explicitly defining migrant status according to ethnic or ancestral background but not country of birth were excluded, as were papers where primary data were not reported (eg, comments, editorials, letters and reviews).

### Search strategy

We individually searched the databases MEDLINE (1946—November 2020), Embase (1974—November 2020), Global Health (1910—November 2020) and PsychINFO (1967—November 2020) within the Ovid platform using a Boolean search strategy with keywords and medical subheadings related to two major themes: migrants and participatory research. There were no geographic or language restrictions. An additional text file outlines the full searches carried out (see online supplemental file 1). The retrieved hits from each database were combined and deduplicated manually within Rayyan. We identified additional studies through hand searching the bibliographies of publications included after full-text screening.

10.1136/bmjopen-2021-053678.supp1 Supplementary data

### Study selection

Two reviewers duplicated the title and abstract screening and full-text screening (KR and SM-H), which was carried out using the web-based application Rayyan.^19^ The reasons for excluding studies during full-text screening were recorded. Any discrepancies in screening decision between the two initial reviewers were mediated by a third reviewer (AC), where retrieved articles indicated the existence of a separate methodological article, we also screened this in conjunction with the first article on condition that it was a retrospective report of methods used (across all stages of the research) rather than a prospective outline of planned methods.

### Data extraction and analysis

Studies that reported using participatory research approaches, and which reported on all stages of the research, were extracted using a piloted form by KR and SH. We extracted summary data on geographical location, the self-described participatory approach, specific target population and aims of the research. Data relating to the participatory approach of each study were extracted and analysed to achieve our primary aim. We extracted data on the stages of the research in which migrants were involved (table 1), where specific research stages did not involve migrants but did involve other non-academic stakeholders this was recorded, subcategorising these groups as community groups/third-sector organisations or professional services. We subsequently extracted data on the methods used to involve migrants (or other non-academic stakeholders) at each stage of the research (inception, design, implementation, analysis, evaluation and dissemination). An additional file provides details of the summary extracted data (see online supplemental file 2).

10.1136/bmjopen-2021-053678.supp2 Supplementary data

**Table 1:** Stages of research and evidence sought for each stage during data extraction

	Research stages					
	Inception	Design	Implementation	Analysis	Evaluation	Dissemination
Evidence sought	Who was responsible for having the idea for the research or initiating the study?	Who was involved in the initial planning and design of the intervention/study? Who decided what the final design of the intervention/study would be?	Who was responsible for implementing/piloting the intervention within the remit of the study? Who decided what this implementation should look like?	Who was involved in analysing data relating to primary endpoints/outcomes? Who decided what these endpoints or outcomes should be?	Who was involved in the overall evaluation of the intervention/study? For example, process evaluation, reflective evaluation? Who had a say in determining successes/failures/future considerations?	Who was involved in disseminating findings? What form did this take? Who decided this?

We related extracted information on who was involved, when they were involved and how they were involved in the described research, relating these factors to participatory principles of inclusion and democracy and categorising them within a framework we developed (table 2). The framework was developed with reference to the literature, particularly that relating to participatory research as a democratic process and being necessary to implement at all stages of the research.^3 5 18 20^ We used data extracted as to who was involved, and when, to guide our assessment of inclusivity. Specifically, we were concerned with whether the evidence displayed relevant inclusivity, that is, the involvement of migrant individuals that are the target or intended beneficiary of the health intervention. We used data extracted as to the method and means of involvement to guide our assessment of democracy. In this instance, we sought evidence of whether methods employed in the research suggested greater levels of democracy, such as through power-sharing and decision-making mechanisms such as equal voting, or committees for those involved. Within the framework, we categorised the aggregated data from each study, with specific reference to migrant individuals, as: active participation; pseudo participation (including proxy and indirect participation) or no explicit evidence of participation. The final framework and definitions were agreed by all coauthors (table 2).

**Table 2:** Framework of the category of participation, with definitions, criteria and relationships to participatory principles applied to aggregated data extracted in this review

Category of participation		Definition	Criteria	Relationship to participatory principles
Active participation		Migrants appeared to be both actively involved, and wielded influence in decisions relating to the research.	Migrant individuals were involved in this stage of the research. And Individuals involved appeared to have direct power and influence over the research stage through shared processes with researchers such as voting or committees.	Relevant inclusivity and greater democracy with regards to migrant involvement.
Pseudo participation	Proxy participation	Community/third-sector organisations and/or professional services are actively involved and wield influence in decisions related to the research stage ahead (or in lieu) of migrants.	There is uncertain/no clear evidence migrant individuals were involved. Or Where migrant individuals were involved, they did not appear to have direct power and influence over the research through shared processes with researchers such as voting or committees. Rather, they were appeared to be involved as research subjects, in surveys or focus groups. But Third-sector organisations and/or professional services were involved. And Third-sector organisation and/or professional services appeared to have direct power and influence over the research stage through shared processes with researchers such as voting or committees.	There may be relevant inclusivity, but lesser democracy with regards to migrant involvement. There may be greater democracy with other stakeholders.
	Indirect participation	Migrants’ involvement is restricted to activities in which they are research subjects (surveys, focus groups, interviews). No other stakeholders appear to be involved.	Where migrant individuals were involved, they did not appear to have direct power and influence over the research through shared processes with researchers such as voting or committees. Rather, they appeared to be involved as research subjects, in surveys or focus groups. And There is uncertain/no clear evidence as to the involvement of third-sector organisations or professional services.	There may be relevant inclusivity, but lesser democracy with regards to migrant involvement. There is no clear evidence of inclusivity or democracy with other stakeholders.
No explicit evidence		No non-academic stakeholders (migrant or otherwise) appear to be involved in this stage of the research.	There is no clear evidence as to the involvement of migrant individuals. There is no clear evidence as to the involvement of third-sector organisations or professional services.	There is no clear evidence of inclusivity or democracy with migrants or other stakeholders.

To achieve our secondary aim, we specifically scanned the included articles for evidence of any evaluation of the use of participatory approaches within the research, inclusive of reflections that appeared in the discussion of included articles. We extracted this data, where found, and categorised it as representing a challenge or benefit associated with the use of participatory approaches toward the overall research process.

### Patient and public involvement

Members of our authorship team have past and current experience of working within third-sector organisations. This experience helped to frame the research questions and definitions used in the analysis. However, lay patients and public specifically were not involved in this research.

## Results

### Screening results

**Figure 1:**
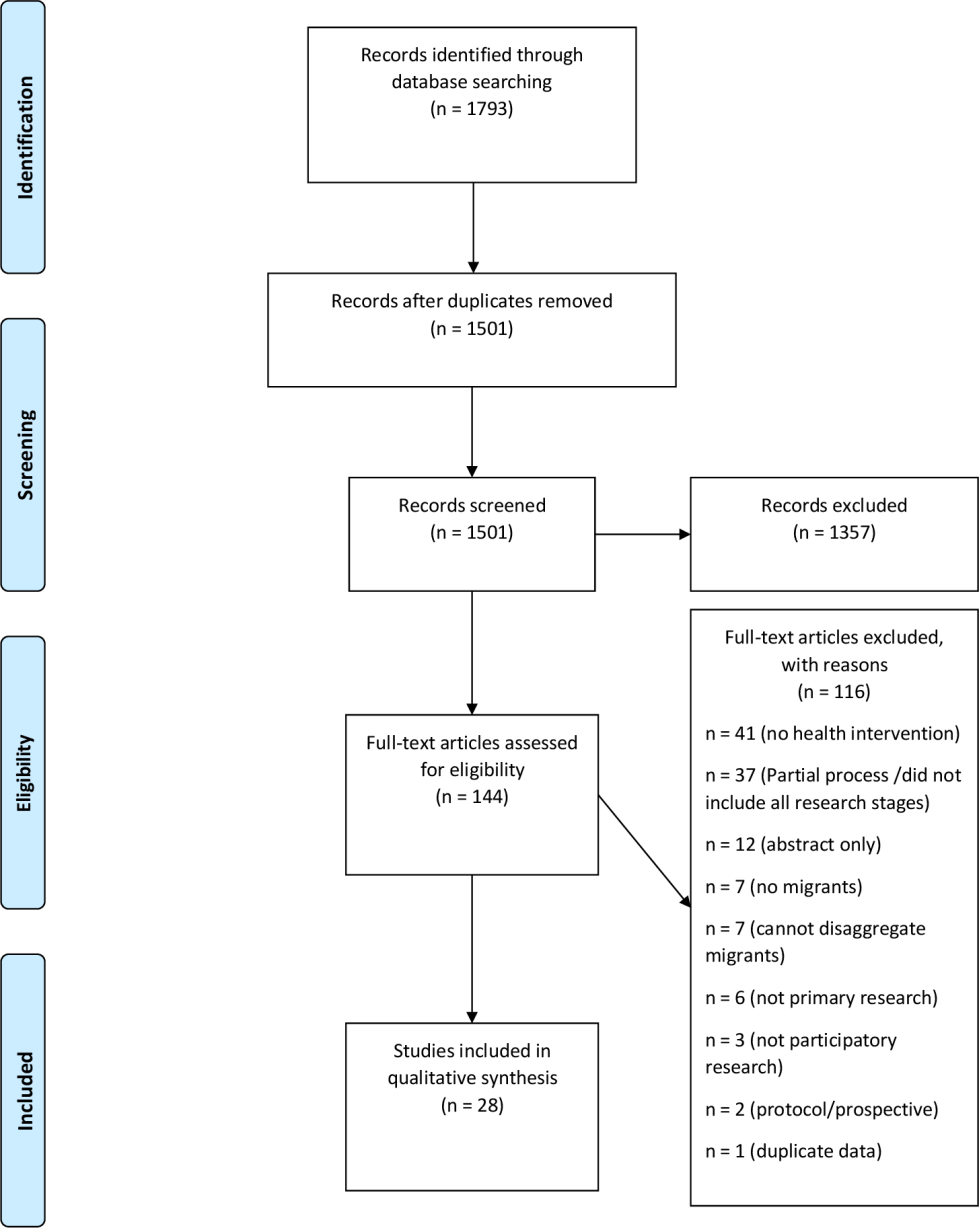
Preferred Reporting Items for Systematic Review and Meta-Analysis flow diagram of the study selection process.

Database searches returned 1793 results; a total of 292 duplicates were removed and 1501 publications were retained for title and abstract screening. Of the 1501 remaining publications, 1357 were excluded during title and abstract screening and 144 were retained for full-text screening. During full-text screening, 116 publications did not meet our criteria and were excluded, with the reasons for exclusion recorded (figure 1). Overall, 28 publications met the inclusion criteria and were included in this systematic review (table 3).

**Table 3:** Descriptive characteristic of studies included in this systematic review

Citation	Location	Self-described participatory approach/methodology	Specific target population	Aim of the health intervention
Afifi *et al*^22^	Lebanon	Community-based participatory research (CBPR)	Palestinian refugee youth	Mental health promotion
Aitaoto *et al*^25^	USA	CBPR	Micronesian women	Cancer outreach/education
Baird *et al*^47^	USA	Community-based collaborative-action research	Sudanese refugee women	Addressing health challenges associated with relocation
Barbee *et al*^26^	USA	CBPR	Haitian immigrant women	To assess the acceptability of human papillomavirus (HPV) self-sampling with community health workers to detect cervical cancer
Chesla *et al*^27^	USA	CBPR	Chinese-American Immigrants	To culturally adapt coping skills training for type-2 diabetes (T2DM)
Evans *et al*^24^	UK	CBPR	African migrants	To promote HIV testing uptake
Forst *et al*^28^	USA	CBPR	Hispanic construction workers	To increase awareness of workplace hazards and self-efficacy; expansion of worker centre agenda to include occupation health and safety
Goodkind *et al*^29^	USA	CBPR	Afghan, Great lakes Region African and Iraqi refugee adults	To address social determinants of health; to improve linkage to mental health services and retention in trauma-focused treatment
Grigg-Saito *et al*^48^	USA	Community-Based Outreach	Cambodian immigrants	Strength-based outreach to eliminate cardiovascular disease and diabetes disparities
Henderson and Slater^21^	Canada	Action Research	Newly arrived migrants	To provide tailored nutritional information and support
Jacquez *et al*^30^	USA	CBPR	Latino immigrants	Stress reduction
Kaiser *et al*^31^	USA	CBPR	Mexican immigrants	To provide obesity prevention education & outreach
Kandula *et al*^32^	USA	CBPR	South Asian immigrant women	Exercise intervention for those at risk of diabetes
Karasz *et al*^33^	USA	CBPR	Bangladeshi immigrant women	To provide and intervention tackling common mental disorders
Kim *et al*^34^	USA	CBPR	Latino immigrants	To use lay health advisors for cardiovascular health promotion
Lam *et al*^35^	USA	CBPR	Vietnamese immigrants	To increase pap screening through education and outreach through lay health workers and media
Li *et al*^36^	USA	CBPR	Chinese-American immigrants	To prevent colorectal cancer through education and outreach
Nilvarangkul *et al*^23^	Thailand	Action Research	Laotian migrants	Enhancement of a quality-of-life model
Pinsker *et al*^37^	USA	CBPR	Somali youth	To provide a culturally appropriate smoking cessation intervention
Quandt *et al*^38^	USA	CBPR	Latino immigrants	To provide Lay health promoter-led pesticide safety education
Solorio *et al*^39^	USA	CBPR	Latino immigrant MSM	To provide HIV prevention outreach for men who have sex with men
Song *et al*^40^	USA	CBPR	Korean-American immigrants	To translate current dietary guidelines into a culturally tailored nutrition programme
Suarez-Balcazar *et al*^41^	USA	CBPR	Latino immigrant families with youth with disabilities	To provide healthy lifestyle promotion
Vaughn *et al*^42^	USA	CBPR	Latino immigrants	To reduce stress and increase coping skills
Wieland *et al*^43^	USA	CBPR	Foreign-born	To promote Tuberculosis screening
Wieland *et al*^44^	USA	CBPR	Immigrants and refugees with type-2 diabetes	To provide a digital story-telling intervention to improve management of type-2 diabetes among those affected
Wieland *et al*^44^	USA	CBPR	Immigrant and refugee women	To provide a physical activity and nutrition programme
Williams *et al*^46^	USA	CBPR	Latino immigrants	Health and safety education in construction

### Study characteristics

The research articles included in this systematic review were published between 2003 and 2019. Only 13 of the publications had any discernible dates relating to when the reported work was conducted, with these dates being between 2003 and 2018. The majority of the publications related to work were carried out in the USA (24 out of 28); the remaining publications related to work were carried out in Canada,^21^ Lebanon,^22^ Thailand^23^ and the UK.^24^ The self-described approach taken by 24 of the 28 included studies was CBPR;^22 24–46^ the remaining four studies were described using community-based collaborative-action research,^47^ community-based outreach^48^ and action research.^21 23^ The dominant focus of the included studies was around education or outreach (table 3), for example, around cancer education,^25^ or healthy lifestyles promotion;^41^ five studies specifically mentioned, including refugees (table 3).^22 29 44 45 47^

### Analysis of participatory approaches in research to develop health interventions for migrants

In our analysis, participation varied substantially according to the stage of the research under scrutiny. Only two of the included studies reported explicit evidence of some degree of participation of at least one non-academic stakeholder groups across all research stages (table 4).^29 30^ Overall, we extracted and categorised evidence of the participation of at least one non-academic stakeholder group in 22 studies during the inception;^21–23 25 26 29–34 36 38–40 42–48^ 25 studies during the design;^21–23 25–39 41–46 48^ 23 studies during implementation;^23–32 34 35 37–43 45–48^ 4 studies during analysis;^29 30 39 43^ 22 studies during evaluation^21 23 24 27–37 39–42 44 46–48^ and 4 studies during dissemination (table 4).^26 29 30 42^

**Table 4:** Analysis and categorisation of participatory character displayed across research stages within included studies

Citation	Research stage					
	Inception	Design	Implementation	Analysis	Evaluation	Dissemination
Afifi *et al*^22^	○	♦	X	X	X	X
Aitaoto *et al*^25^	●	♦	♦	X	X	X
Baird *et al*^47^	♦	X	○	X	○	X
Barbee *et al*^26^	♦	♦	♦	X	X	♦
Chesla *et al*^27^	X	♦	●	X	○	X
Evans *et al*^24^	X	X	○	X	○	X
Forst *et al*^28^	X	●	●	X	♦	X
Goodkind *et al*^29^	♦	♦	♦	♦	♦	♦
Grigg-Saito *et al*^48^	♦	♦	♦	X	♦	X
Henderson and Slater^21^	●	○	X	X	○	X
Jacquez *et al*^30^	♦	♦	♦	♦	♦	♦
Kaiser *et al*^31^	○	♦	○	X	○	X
Kandula *et al*^32^	●	●	●	X	●	X
Karasz *et al*^33^	●	♦	X	X	○	X
Kim *et al*^34^	●	♦	♦	X	♦	X
Lam *et al*^35^	X	♦	♦	X	♦	X
Li *et al*^36^	●	●	X	X	○	X
Nilvarangkul, McCann. 2011	♦	♦	●	X	●	X
Pinsker *et al*^37^	X	♦	♦	X	○	X
Quandt *et al*^38^	●	●	●	X	X	X
Solorio *et al*^39^	●	○	●	○	○	X
Song *et al*^40^	●	X	●	X	○	X
Suarez-Balcazar *et al*^41^	X	♦	♦	X	○	X
Vaughn *et al*^42^	♦	♦	♦	X	○	♦
Wieland *et al*^43^	●	●	●	●	X	X
Wieland *et al*^44^	●	○	X	X	○	X
Wieland *et al*^45^	●	○	●	X	X	X
Williams *et al*^46^	●	♦	♦	X	○	X

**♦Active participation:** Migrants appeared to be both actively involved, and wielded influence in decisions relating to the research.

**●Proxy participation:** Community/third-sector organisations and/or professional services are actively involved and wield influence in decisions related to the research stage ahead (or in lieu) of migrants.

**○Indirect participation**: Migrants’ involvement is restricted to activities in which they are research subjects (surveys, focus groups, interviews). No other stakeholders appear to be involved.

X **No explicit evidence:** No non-academic stakeholders (migrant or otherwise) appear to be involved in this stage of the research.

However, there was greater variation and divergence in participatory approaches when considering the degree of participation of migrants. In our analysis, only 18 of the 28 included studies exhibit active participation of migrants (as the primary focus and target of the intervention) at any stage of the research process.^22 23 25–31 33–35 37 41 42 46–48^ Of these 18 studies, only 2 display evidence of active participation of migrants at all stages of the research process.^29 30^

Across all 168 individual research stages analysed across the 28 studies, we recorded 46 instances of active participation of migrants; 30 instances of proxy participation; 24 instances of indirect participation and 68 instances in which there was insufficient evidence to make a determination (table 4). The active participation recorded also appears to be associated with the stage of the research. There were 7 instances of active participation during study inception;^23 26 29 30 42 47 48^ 16 during design;^22 23 25–27 29–31 33–35 37 41 42 46 48^ 10 during implementation;^25 26 29 30 34 35 37 41 42 46 48^ 2 during analysis;^29 30^ 6 during evaluation^28–30 34 35 48^ and 4 during dissemination.^26 29 30 42^

### Evidence of the benefits of using a participatory approach to research

The benefit most often reported among the included articles in using participatory approaches was the assertion that interventions were better tailored to the target population through involving non-academic stakeholders.^22 25 27 32 37 41 42 46 49^ This included two studies, which spoke of the benefits of participatory research in facilitating interventions going beyond more immediately actionable cultural adaptations (such as language adaptation and ethnically matched providers), to provide interventions that more deeply reflect community values and priorities.^27 42^

Participatory approaches provided benefits through the partnerships established during the research. One study reported how participatory approaches allowed for the modification of the research programme throughout conception, development and implementation.^34^ Multiple publications provided evidence on how iterative feedback from stakeholders during the studies could further grow partnerships, improving the recruitment of individuals to implement or take part in the intervention^21 22 27 32 42 50^ and dissemination.^27^ One study also highlighted that partnerships were a feasible and appropriate means to support intervention implementation,^24^ while one set of authors reported that partnerships with non-academics can ultimately strengthen research.^26^

Better relationships between the community and academics were cited as having the capability to enhance the familiarity and trust of individuals involved in participatory research. One study cited that increased trust had direct benefits to research, leading to more open and honest dialogue than in traditional research, improving the accuracy and findings of these activities.^47^ Researchers becoming part of ongoing community relations was seen as positive, or a catalyst, acting as an impartial bridge between disparate community groups.^22^ Long-lasting partnerships built over the course of participatory research studies were cited as producing a capacity-building element, increasing the health-related knowledge and resources of the community, which academics partnered with.^28 35 43^ Finally, partnerships catalysed a greater degree of understanding of a subject among communities, leading to increased self-determination and the ability to generate change of their own accord.^47^

### Evidence of the challenges of using a participatory approach to research

Multiple studies highlighted the importance of balancing the culture and expectations of both researchers and migrant individuals to enact participatory research.^22 27 39^ For example, one study reported that reaching equitability in the research process and working on level-terms with migrants directly conflicted with the cultural norms of some of these individuals, who may revere authority figures, and so would in normal circumstances defer to their judgement.^47^ A further study highlighted challenges exist in bringing together differing stakeholders with varied views and experiences. In these situations, it was suggested there is no ‘one size fits all’ approach and that processes must be adapted to individual groups.^35^ Noting varied perspective, one study highlights the challenge that divergent perspectives as to what is most salient and important to address among those involved can present a challenge.^29^

The challenge (and importance) of building rapport and addressing mistrust,^22 23 48^ or even research fatigue among some groups,^22^ was also evident within publications. One set of authors identified the need for non-academic stakeholders to trust researchers alongside the need for researchers to reciprocate this trust, and prioritise the collaborative and democratic aim of participatory methods. This was perceived as challenging as it may shift the power dynamic and locus of control in the research away from the academics.^38^ Even when partnerships overcome challenges of culture, expectations and trust, there remain other practical challenges to operationalising these partnerships.^33 37^

Challenges in ensuring equitability in research understanding, and balancing the participatory nature of a project, with the standards expected by the wider scientific community were also highlighted.^22 35^ Furthermore, one study cited the difficulty of navigating acknowledgement and authorship of non-academics in published materials;^27^ a scenario that serves to reiterate power imbalances that can often persist,^51^ in that despite being ‘equal partners’ in research, migrants may still not be equally recognised. The lack of recognition of the requirements of participatory research in traditional academic circles is also cited as a challenge, with one set of authors stating the need for managerial, institutional and funder-level buy-in and commitment regarding participatory research.^22^ Similarly, institutional review limited participation in at least one study, preventing non-academic stakeholders’ involvement in data collection and analysis.^35^

Other practical challenges to operationalising participatory research included effective, timely communication,^23 36^ and the challenge of working with communities in which the dominant language of the researchers and migrant communities differ.^39 47^ Finally, the iterative and tailored nature of the interventions produced may also impact the generalisability of findings,^41^ while some work could seemingly omit or contradict research evidence due to localising the intervention.^44^

## Discussion

To our knowledge, this is the first systematic review to robustly measure the application of participatory approaches and principles to health intervention research with migrants and specifically examine how core participatory principles of inclusivity and democracy are reflected in this application. While specifically focusing on research with migrants, many of the findings and the framework discussed are likely to provide insight into all practitioners of participatory research. The 28 studies included reported on a variety of developed health interventions, predominantly revolving around outreach and education. Our analysis shows that 18 of the 28 included studies actively involved migrants themselves, but only 2 studies actively involved migrants during all stages of the research process. The remaining studies either provide insufficient evidence to determine the participatory approach used or were characterised by pseudoparticipation, in which community groups/third-sector organisations were directly involved (proxy-participation), or migrants were only involved through being subjects in research activities (indirect participation).

The participatory approaches taken to develop interventions in the included studies varied. The examples that represent the most participatory approach, according to our analysis, were characterised by consistent relevant inclusivity and greater democracy, which saw co-operation between researchers and migrant individuals (whom had power on par with the researchers) (table 5). The difference between active participatory approaches and those we characterised as pseudoparticipation appear subtle when viewed from a research-centric perspective but are stark when considering a participatory perspective. First, indirect participation, in which migrants are involved in activities such as surveys or interviews designed to inform health interventions, may represent a perfectly suitable means to guide development and build evidence, but does little to distribute power in a participatory manner. The risk that research is framed as participatory while failing to develop equitable partnerships has previously been highlighted and still appears to persist.^3 52^ There is also concern that participatory research continues to be one-sided, with a continued focus on and glorification of methods on the part of researchers in studies involving migrants, at the expense of participatory principles.^53^ Second, proxy participation, which may do more to uphold principles of participatory research, may still be at risk of not equitably involving the actual target population. Uncertainty persists around how to best involve non-academic stakeholders and ensure those that are involved are representative of the population of interest.^54^ While community-groups and/or professional service involvement may at times be the only, or most readily available way to represent the population of interest (due to difficulties (perceived or otherwise) in accessing, or providing access to migrants), they cannot be assumed to be representative of them. Previous research has shown that health-service users can identify different needs to service providers.^55^ Furthermore, while overall understandings of involvement processes may align, service providers may place different values on some aspects of involvement.^56^ Therefore, proxy participation could conceivably skew participatory research away from being centred on migrants’ needs.

**Table 5:** Descriptive tabulation of two studies classified as displaying active participation throughout all stage of the health intervention research with migrants

		Research stage					
		Inception	Design	Implementation	Analysis	Evaluation	Dissemination
Studies	Goodkind *et al*^29^	Study conceived from previous relations with community groups. The present study was guided by refugees and community service providers.	The community was involved in designing interview protocols and participant recruitment procedures.	All interpreters and interviewers were refugees; procedures had been agreed during inception and design.	Refugees were involved in analysis and were actively involved in setting the agenda for what evidence was meaningful.	Refugees and community involved in discussions to evaluate the process; indication that the decision a community intervention paradigm be adopted appears to have been adopted and championed by researchers as a result.	Refugees were involved in the dissemination and are co-authors of the research publication.
	Jacquez *et al*^17^	Manifested from a previous relationship with latinos unidos por la salud to promote health and healthcare for the local latino community; co-researchers in this project were drawn from the local community.	Co-researchers worked with academic partners to identify primary outcomes and helped decide that health worker delivered strategies were the preferred intervention option	Co-researchers recruited and worked with participants to identify strategies for stress reduction.	Co-researchers and academic partners identified the primary outcomes.	Academic and community partners shared decision-making in all aspects of the research process, including evaluation.	Academic and community partners shared decision-making in all aspects of the research process, including dissemination.

Upholding the core principles of participatory research, in this instance, democratising research and power sharing, is particularly pertinent to partnering with migrants. Participatory research origins are firmly rooted in increasing social justice, and the promotion of doing research with, not on or about individuals and communities, particularly those that are disadvantaged.^52^ Migrant communities are often marginalised within recipient countries,^13 14^ and by local health systems.^11 12^ Our categorisations, and the challenges and considerations we highlight speak to the deeper underlying influence of power dynamics, which are present in all research and interactions and can manifest at individual, interpersonal and structural levels within participatory research.^51^ These dynamics should not be overlooked, regardless of the perceived benefits and potential of participatory research approaches, lest participatory research unconsciously becomes a means to reinforce and further entrench power inequity individuals such as migrant participants may experience. Not only is it inappropriate for research to perpetuate or deepen any marginalisation through failing to include migrants’ voices, insights and skills, but there are also benefits to the proper utilisation of participatory approaches to the overall research process. Included studies provide evidence of the benefits to participant recruitment, implementation and dissemination. Researchers also highlighted that the iterative nature of participatory research allows more effective tailoring of work to the needs of migrants, through learning from and embedding migrant partners’ knowledge and experience into research. While studies we identify predominantly focus on community outreach and education within health research, participatory research could be better used across all disciplines. Similar methodology could be employed to better design pharmaceuticals, or on a larger scales, procedures and systems at a governance level.

Effectively partnering with migrants requires specific strategies to address the challenges identified in this review. Some of these strategies include early participatory involvement to guide research priorities, methodological approaches and strategies to manage ongoing relations; translating and back-translating materials; giving reassurance as to the confidentiality of involvement and respecting decisions around reporting (particularly as some partners may be undocumented migrants or have precarious legal status); using a variety of outreach and recruitment outlets, such as non-governmental organisations () and religious groups trusted by migrants and identifying opportunities for bidirectional benefits in the research, and capacity building to facilitate collaborative and democratic participation. Those partnering with migrants must demonstrate flexibility to negotiate potential power divides, and acknowledge and be considerate of residual mistrust that may exist among communities, even after researcher-community relationships appear well established.^57^ The challenges and extra consideration highlighted by this review must not be underestimated, while from a research perspective, more still needs to be done to assess the impact of participatory approaches on overall research processes and output as well as assessing whether there are distinct benefits to adopting particular participatory approaches (eg, active, pseudo). However, if research is to become more democratic, patient-centred and representative of the populations impacted by its work, traditional scientific approaches are likely to be inadequate, with academic researchers holding the majority of power over research.^58^

Greater adoption of consistent and transparent reporting of participatory research is needed to support the need for more critical analysis of involvement and participatory research.^17 59^ While guidelines have been developed,^60^ they have not been widely adopted, with no material improvement in the reporting quality of published studies seen within some fields as a result of the their publication, which could be attributed to a lack of awareness of the guidelines existence.^61^ Tensions exist as to whether participatory research should be conceptualised and evaluated similarly to traditional research.^59 62^ However, we believe reporting can be sympathetic to the need to evidence impact of methods and processes on research. We propose the plain reporting of: who was involved in each element of the research; why these individuals were involved; how they were involved and who ultimately controlled decisions relating to the research. These questions should be answered by all research involving non-academic stakeholders, at every stage of the research process. The development of guidelines to support this reporting would simultaneously allow a more complete assessment of how participatory approaches impacted overall outcomes, and greater reflection and evaluation of the participatory approaches employed;^62^ such guidelines and evaluative methods should incorporate and build on existing monitoring tools, such as those which specifically seek to address existing challenges around power dynamics.^51^ Any guidelines must also consider the distinct nature of participatory research, in that conventional evaluation is likely inappropriate with participatory research, and consequently, its’ evaluation may emphasise internal group evaluation, which is done for combined stakeholders in an adaptive and negotiated manner.^63^

Comparing our review to existing literature, there appears to be a common trend where academics and research as whole are primarily concerned with the impact or benefits of participatory approaches on research processes and outcomes, which is a valid question, but it is only encompassed in our research as a secondary aim. Nevertheless, our findings as to challenges and benefits corroborate and align with existing research that participatory approaches can provide benefits particularly to the recruitment and retention of trial participants.^64–67^ Challenges associated with participatory research that are previously reported and complementary to our findings often involve methodological challenges around collecting, interpreting and disseminating research.^64 66^ We found, similar to previous reviews, that the extent of engagement and involvement of non-academic stakeholders is highly varied, and that research that is expressly participatory is often limited.^17 64–67^ We also find similar trends to these previous reviews in that there is also variability in the use of naming conventions and application of reported approaches across all fields and topics of research.^17 65^

Our review has several limitations and caveats. First, we acknowledge the taxonomy of terminology around participatory research is not standardised and terms are used inconsistently. This is an ongoing challenge within the field, which has previously been evidenced in similar reviews.^17^ As such, the categorisations we have introduced and used in this review may be defined differently by others. There is also the possibility that additional publications that do not explicitly use the same language as we have are present in the literature, and that these articles may have been missed by our searches. Furthermore, whether the included studies categorised in our analysis exhibit greater or lesser participation are potentially immaterial to the quality of the research carried out, or the impact of the final intervention; quality criteria for participatory research have not been agreed on or widely adopted. The amalgamation of these limitations is that formal assessments of study quality, certainty around evidence and reporting biases are not readily applied to this systematic review as for more homogenised approaches and methods, such as clinical trials. A further consideration relates to the reporting of benefits and challenges, in that it is often unclear, which individuals or groups drove evaluations, and whether academics alone or academics and communities in partnership decided on what was reported. We have sought not only to include evidence from formal evaluation where possible but also to include evidence from articles as a whole, such as in the discussion, which could be considered reflective in nature, and potentially less rigorous on the whole. It is conceivable, given the subjective nature of some of the reported items, that differing sets of benefits and challenges would be reported dependent on the populations involved. This review represents our attempt to cast a critical lens over how the principles of participatory research are applied in practice. Our conceptual focus on migrants’ involvement is, therefore, not intended to denigrate the efforts of third-sector organisations or professional services, whose involvement we may have classified as proxy participation. Fundamentally, we believe that the examined studies are inherently more participatory than traditional research endeavours, for having even considered and attempted to involve non-academic stakeholders. We recognise the challenges associated with participatory research, and as stated, hold no assumptions about the extent of participation and its’ association with beneficial outcomes for target populations.

## Conclusion

In conclusion, participatory approaches to developing health interventions aimed at migrants are insufficiently applied and reported. We provide evidence that the application of approaches does not fully embody core principles of participatory research, particularly relating to providing decision-making power to individuals ultimately affected by the research. Those who wish to engage in participatory research must consider the approach they take, being cognisant and open to reflecting on questions of representation, democracy and overall power dynamics, and from this critically analysing whether their approach is sufficient to achieve high-quality participation, not just high-quality research. Crucially, guidelines for reporting of participatory research methods must be introduced. This will enable all parties, from academics to communities to better assess the participatory nature of individual research projects and is an important prerequisite to explore the overall impact of participatory research, which currently remains inadequately understood.

## Supplementary Material

Author's manuscript

## Data Availability

All data relevant to the study are included in the article or uploaded as supplementary information. Availability of data and materials. All data generated or analysed during this study are included in this published article.Availability of data and materials. All data generated or analysed during this study are included in this published article.Availability of data and materials. All data generated or analysed during this study are included in this published article.
