# Supplementary material for: Participatory approaches in the development of health interventions for migrants: a systematic review
Source: BMJ Open. 2021 Oct 25;11(10):e053678. doi: 10.1136/bmjopen-2021-053678 (PMC8548676; doi:10.1136/bmjopen-2021-053678)
Supplement: Supplementary data [file bmjopen-2021-053678supp001.pdf]

Additional File 1. Full reproduction of searches used within the individual databases

MEDLINE Database Search Strategy (1946 – November 2020):

1. "Transients and Migrants"/ or Migrant*.mp.
2. Migrat*.mp. [mp=title, abstract, heading word, drug trade name, original title, device manufacturer, drug manufacturer, device trade name, keyword, floating subheading word, candidate term word]
3. Refugee*.mp. or Refugees/
4. Asylum-seeker*.mp.
5. Foreign*.mp.
6. Foreign-born.mp.
7. Non-native*.mp.
8. "Emigrants and Immigrants"/ or Immigra*.mp.
9. Emigra*.mp. or "Emigration and Immigration"/
10. Oversea*.mp.
11. Foreign student*.mp.
12. International Student*.mp.
13. 1 or 2 or 3 or 4 or 5 or 6 or 7 or 8 or 9 or 10 or 11 or 12
14. Co-design*.mp.
15. Co-prod*.mp. [mp=title, abstract, heading word, drug trade name, original title, device manufacturer, drug manufacturer, device trade name, keyword, floating subheading word, candidate term word]
16. Co-creat*.mp. [mp=title, abstract, heading word, drug trade name, original title, device manufacturer, drug manufacturer, device trade name, keyword, floating subheading word, candidate term word]
17. Collab* design*.mp. [mp=title, abstract, heading word, drug trade name, original title, device manufacturer, drug manufacturer, device trade name, keyword, floating subheading word, candidate term word]
18. Collab* prod*.mp. [mp=title, abstract, heading word, drug trade name, original title, device manufacturer, drug manufacturer, device trade name, keyword, floating subheading word, candidate term word]
19. Collab* creat*.mp. [mp=title, abstract, heading word, drug trade name, original title, device manufacturer, drug manufacturer, device trade name, keyword, floating subheading word, candidate term word]
20. Community design*.mp.
21. Community prod*.mp.
22. Community creat*.mp.
23. Community-based participatory research.mp. [mp=title, abstract, heading word, drug trade name, original title, device manufacturer, drug manufacturer, device trade name, keyword, floating subheading word, candidate term word]
24. Participatory design*.mp.
25. Participatory action research.mp. or Community-Based Participatory Research/
26. (Participatory adj4 Research).mp. [mp=title, abstract, heading word, drug trade name, original title, device manufacturer, drug manufacturer, device trade name, keyword, floating subheading word, candidate term word]
27. Action Research.mp.
28. 14 or 15 or 16 or 17 or 18 or 19 or 20 or 21 or 22 or 23 or 24 or 25 or 26 or 27
29. 13 and 28

Embase Database Search Strategy (1974 – November 2020):

1. migrant/ or migrant worker/ or Migrant*.mp.
2. migration/ or Migrat*.mp.
3. refugee/ or Refugee*.mp.
4. asylum seeker/ or Asylum seeker*.mp.
5. Foreign*.mp.
6. Foreign Born.mp.
7. Non-native*.mp.
8. immigration/ or immigrant/ or Immigra*.mp.
9. emigrant/ or emigration/ or Emigrants/ or Emigra*.mp.
10. Oversea*.mp.

11. Foreign Student*.mp. or foreign student/
12. International Student*.mp.
13. 1 or 2 or 3 or 4 or 5 or 6 or 7 or 8 or 9 or 10 or 11 or 12
14. Co-design*.mp.
15. Co-prod*.mp.
16. Co-creat*.mp.
17. Collab* design*.mp.
18. Collab* prod*.mp.
19. Collab* creat*.mp.
20. Community design*.mp.
21. Community prod*.mp.
22. Community creat*.mp.
23. Community-based participatory research.mp. [mp=title, abstract, heading word, drug trade name, original title, device manufacturer, drug manufacturer, device trade name, keyword, floating subheading word, candidate term word]
24. Action research.mp. or action research/
25. Participatory research.mp. or participatory research/
26. Participatory action research.mp.
27. (Participatory adj4 Research).mp. [mp=title, abstract, heading word, drug trade name, original title, device manufacturer, drug manufacturer, device trade name, keyword, floating subheading word, candidate term word]
28. 14 or 15 or 16 or 17 or 18 or 19 or 20 or 21 or 22 or 23 or 24 or 25 or 26 or 27
29. 13 and 28

Global Health Database Search Strategy (1920 – November 2020):

1. Migrant*.mp.
2. Migrat*.mp. [mp=title, abstract, heading word, drug trade name, original title, device manufacturer, drug manufacturer, device trade name, keyword, floating subheading word, candidate term word]
3. Refugee*.mp. or refugees/
4. Asylum-seeker*.mp.
5. Foreign*.mp.
6. Foreign-born.mp.
7. Non-native*.mp.
8. Immigra*.mp.
9. Emigra*.mp. [mp=title, abstract, heading word, drug trade name, original title, device manufacturer, drug manufacturer, device trade name, keyword, floating subheading word, candidate term word]
10. Oversea*.mp.
11. Foreign Student*.mp. or foreign students.sh.
12. International Student*.mp.
13. 1 or 2 or 3 or 4 or 5 or 6 or 7 or 8 or 9 or 10 or 11 or 12
14. Co-design*.mp.
15. Co-prod*.mp. [mp=title, abstract, heading word, drug trade name, original title, device manufacturer, drug manufacturer, device trade name, keyword, floating subheading word, candidate term word]
16. Co-creat*.mp. [mp=title, abstract, heading word, drug trade name, original title, device manufacturer, drug manufacturer, device trade name, keyword, floating subheading word, candidate term word]
17. Collab* design*.mp. [mp=title, abstract, heading word, drug trade name, original title, device manufacturer, drug manufacturer, device trade name, keyword, floating subheading word, candidate term word]
18. Collab* prod*.mp. [mp=title, abstract, heading word, drug trade name, original title, device manufacturer, drug manufacturer, device trade name, keyword, floating subheading word, candidate term word]
19. Collab* creat*.mp. [mp=title, abstract, heading word, drug trade name, original title, device manufacturer, drug manufacturer, device trade name, keyword, floating subheading word, candidate term word]
20. Community design*.mp.
21. Community prod*.mp. [mp=title, abstract, heading word, drug trade name, original title, device manufacturer, drug manufacturer, device trade name, keyword, floating subheading word, candidate term word]
22. Community creat*.mp.

23. Community-based participatory research.mp. [mp=title, abstract, heading word, drug trade name, original title, device manufacturer, drug manufacturer, device trade name, keyword, floating subheading word, candidate term word]
24. Participatory design*.mp.
25. Participatory action research.mp.
26. (Participatory adj4 Research).mp. [mp=title, abstract, heading word, drug trade name, original title, device manufacturer, drug manufacturer, device trade name, keyword, floating subheading word, candidate term word]
27. Action research.mp.
28. 14 or 15 or 16 or 17 or 18 or 19 or 20 or 21 or 22 or 23 or 24 or 25 or 26 or 27
29. 13 and 28

PsychINFO Database Search Strategy (1967 – November 2020):

1. Migrant*.mp.
2. Migrat*.mp.
3. exp REFUGEES/ or Refugee*.mp.
4. exp Asylum Seeking/ or Asylum-seeker*.mp.
5. Foreign*.mp.
6. Foreign-born.mp.
7. Non-native*.mp.
8. exp Immigration/ or Immigra*.mp.
9. Emigra*.mp. [mp=title, abstract, heading word, drug trade name, original title, device manufacturer, drug manufacturer, device trade name, keyword, floating subheading word, candidate term word]
10. Oversea*.mp. [mp=title, abstract, heading word, drug trade name, original title, device manufacturer, drug manufacturer, device trade name, keyword, floating subheading word, candidate term word]
11. Foreign Student*.mp.
12. exp International Students/ or International Student*.mp.
13. 1 or 2 or 3 or 4 or 5 or 6 or 7 or 8 or 9 or 10 or 11 or 12
14. Co-design*.mp.
15. Co-prod*.mp. [mp=title, abstract, heading word, drug trade name, original title, device manufacturer, drug manufacturer, device trade name, keyword, floating subheading word, candidate term word]
16. Co-creat*.mp. [mp=title, abstract, heading word, drug trade name, original title, device manufacturer, drug manufacturer, device trade name, keyword, floating subheading word, candidate term word]
17. Collab* design*.mp.
18. Collab* prod*.mp. [mp=title, abstract, heading word, drug trade name, original title, device manufacturer, drug manufacturer, device trade name, keyword, floating subheading word, candidate term word]
19. Collab* creat*.mp. [mp=title, abstract, heading word, drug trade name, original title, device manufacturer, drug manufacturer, device trade name, keyword, floating subheading word, candidate term word]
20. Community design*.mp.
21. Community prod*.mp.
22. Community creat*.mp. [mp=title, abstract, heading word, drug trade name, original title, device manufacturer, drug manufacturer, device trade name, keyword, floating subheading word, candidate term word]
23. Community-based participatory research.mp. [mp=title, abstract, heading word, drug trade name, original title, device manufacturer, drug manufacturer, device trade name, keyword, floating subheading word, candidate term word]
24. Participatory design*.mp.
25. Participatory research.mp.
26. (Participatory adj4 Research).mp. [mp=title, abstract, heading word, drug trade name, original title, device manufacturer, drug manufacturer, device trade name, keyword, floating subheading word, candidate term word]
27. Participatory action research.mp.
28. Action research.mp. or exp Action Research/
29. 14 or 15 or 16 or 17 or 18 or 19 or 20 or 21 or 22 or 23 or 24 or 25 or 26 or 27 or 28
30. 13 and 29
