# Supplementary material for: Participatory approaches in the development of health interventions for migrants: a systematic review
Source: BMJ Open. 2021 Oct 25;11(10):e053678. doi: 10.1136/bmjopen-2021-053678 (PMC8548676; doi:10.1136/bmjopen-2021-053678)
Supplement: Author's manuscript [file bmjopen-2021-053678.draft_revisions.pdf]

BMJ Open is committed to open peer review. As part of this commitment we make the peer review history of every article we publish publicly available.

When an article is published we post the peer reviewers' comments and the authors' responses online. We also post the versions of the paper that were used during peer review. These are the versions that the peer review comments apply to.

The versions of the paper that follow are the versions that were submitted during the peer review process. They are not the versions of record or the final published versions. They should not be cited or distributed as the published version of this manuscript.

BMJ Open is an open access journal and the full, final, typeset and author-corrected version of record of the manuscript is available on our site with no access controls, subscription charges or pay-per-view fees (http://bmjopen.bmj.com).

If you have any questions on BMJ Open's open peer review process please email
info.bmjopen@bmj.com

BMJ Open

Participatory research in health intervention studies involving migrants: a systematic review

Journal:	*BMJ Open*
Manuscript ID	bmjopen-2021-053678
Article Type:	Original research
Date Submitted by the Author:	26-May-2021
Complete List of Authors:	Rustage, Kieran; St George's University of London, Institute for Infection and Immunity<br>Crawshaw, Alison; St George's University of London, Institute for Infection and Immunity<br>Majeed-Hajaj, Saliha; Queen Mary University of London<br>Deal, Anna; St George's University of London, Institute for Infection and Immunity; London School of Hygiene & Tropical Medicine<br>Nellums, Laura ; University of Nottingham, Division of Epidemiology and Public Health<br>Ciftci, Yusuf; Doctors of the World<br>Fuller, Sebastian; St George's University of London, Institute for Infection and Immunity<br>Goldsmith, Lucy; St George's University of London, Institute for Infection and Immunity, and Population Health Research Institute;<br>Friedland, Jon; St George's University of London<br>Hargreaves, Sally; St George's University of London, Institute for Infection and Immunity
Keywords:	PUBLIC HEALTH, STATISTICS & RESEARCH METHODS, EDUCATION & TRAINING (see Medical Education & Training)

SCHOLARONE™
Manuscripts

I, the Submitting Author has the right to grant and does grant on behalf of all authors of the Work (as defined in the below author licence), an exclusive licence and/or a non-exclusive licence for contributions from authors who are: i) UK Crown employees; ii) where BMJ has agreed a CC-BY licence shall apply, and/or iii) in accordance with the terms applicable for US Federal Government officers or employees acting as part of their official duties; on a worldwide, perpetual, irrevocable, royalty-free basis to BMJ Publishing Group Ltd ("BMJ") its licensees and where the relevant Journal is co-owned by BMJ to the co-owners of the Journal, to publish the Work in this journal and any other BMJ products and to exploit all rights, as set out in our licence.

The Submitting Author accepts and understands that any supply made under these terms is made by BMJ to the Submitting Author unless you are acting as an employee on behalf of your employer or a postgraduate student of an affiliated institution which is paying any applicable article publishing charge ("APC") for Open Access articles. Where the Submitting Author wishes to make the Work available on an Open Access basis (and intends to pay the relevant APC), the terms of reuse of such Open Access shall be governed by a Creative Commons licence – details of these licences and which Creative Commons licence will apply to this Work are set out in our licence referred to above.

Other than as permitted in any relevant BMJ Author's Self Archiving Policies, I confirm this Work has not been accepted for publication elsewhere, is not being considered for publication elsewhere and does not duplicate material already published. I confirm all authors consent to publication of this Work and authorise the granting of this licence.

For peer review only

Participatory research in health intervention studies involving migrants: a systematic review

Kieran Rustage,[1] Alison Crawshaw,[1] Saliha Majeed-Hajaj,[2] Anna Deal,[1] Laura B Nellums,[3] Yusuf Ciftci,[4] Sebastian Fuller,[1] Lucy Goldsmith,[1] Jon S Friedland,[1] Sally Hargreaves[1]

Corresponding author: Sally Hargreaves (s.hargreaves@sgul.ac.uk)

Word count: 3888

Key Words: Participatory Research; Systematic Review; Migrant Health; Health Intervention

Institutional affiliations:

1. Institute for Infection & Immunity, St George's University of London, United Kingdom
2. Queen Mary University of London, London, United Kingdom
3. University of Nottingham, Nottingham, United Kingdom
4. Doctors of the World UK, part of the Médicins du Monde network

For peer review only - http://bmjopen.bmj.com/site/about/guidelines.xhtml

Abstract

Objective

To analyse the use of participatory approaches in research of health interventions for migrants, and how utilised approaches embody core participatory principles of democracy and power-distribution.

Design

A systematic review of original articles. Electronic searchers were carried out in the databases MEDLINE, Embase, Global Health and PsychINFO (from inception – Nov 2020).

Eligibility criteria for study selection

The analysis included original peer-reviewed research which reported on attempts to develop and implement a health intervention for migrants using participatory approaches. We defined migrants as foreign-born individuals; studies using definitions demonstrably outside of this were excluded. Only articles reporting the full research cycle (inception, design, implementation, analysis, evaluation, dissemination) were included.

Data extraction

Information related to who was involved in research (migrants or other non-academic stakeholders), the research stage at which they were involved (inception, design, implementation, analysis, evaluation, dissemination), and how this involvement aligned with the core principles of participatory research – categorising studies as exhibiting active, pseudo, or indirect participation of migrants.

Results

For peer review only

1793 publication were screened of which 28 were included in our analysis. We found substantial variation in the application of participatory research approaches: across 168 individual research stages analysed across the 28 studies, we recorded 46 instances of active participation of migrants; 30 instances of proxy participation; and 24 instances of indirect participation. Whilst all studies involved at least one non-academic stakeholder group in at least one stage of the research, just two studies exhibited evidence of active participation of migrants across all research stages.

Conclusions

These data highlight important shortfalls in the inclusion of migrant groups in developing health interventions that affect their lives and suggest a more rigorous and standardised approach to defining and delivering participatory research is urgently needed to improve the quality of participatory research.

Registration

This review followed PRISMA guidelines and is registered on the Open Science Framework (osf.io/2bnz5)

For peer review only - http://bmjopen.bmj.com/site/about/guidelines.xhtml

For peer review only

Strengths and Limitations

- This systematic review represents a robust and novel assessment of the applications of participatory approaches and principles to health intervention research with migrants.
- This review casts a critical lens over the application and outcomes of participatory approaches, conceptually focusing on the relationship between the methods used and the populations involved, and how this all relates to participatory principles.
- This review is limited by the varied and interchangeable use of participatory research terms within this field. The categorisations and terms we introduce may therefore be defined differently by others.
- This review is limited by the lack of clear and consistent reporting of participatory methods utilised; guidelines must be prepared and consistently adopted to improve transparency in all participatory research.
- This does not analyse or consider associations between participatory methods, and health or research outcomes; future research to understand any such associations is needed.

For peer review only

For peer review only - http://bmjopen.bmj.com/site/about/guidelines.xhtml

Introduction

Considerable emphasis is now being placed on ensuring patient and public engagement in health research, including striving for greater involvement of marginalised groups such as migrants and ethnic minorities.[1,2] However, whether this is effectively and meaningfully done in practice to ensure truly patient-centred research has yet to be fully elucidated. Participatory research represents a distinct research paradigm in which research is done collaboratively the individuals whose lived experiences and actions are the subject of study, as active partners who share power and influence over research processes and outcomes.[3-6]

The fundamental principles of participatory research are those of inclusivity and democracy, particularly in relation to those directly affected by the research in question.[3] Included under the umbrella of participatory research approaches are more specific methodologies which look to uphold these principles, including: community based participatory research (CBPR); action research; some patient & public involvement (PPI); as well as broader derivatives such as community-based collaborative action research. Participatory research holds the potential to bridge the gap between public health research and practice, creating a context in which patients and the public have meaningful influence over research decisions, increasing the relevance and impact of research outcomes to their own lives.[7]

Participatory research is likely to be particularly powerful when working with underserved and marginalised groups such as migrants, where traditional research has frequently failed to provide an appreciable health benefit. Whilst a heterogenous group, comprising a multitude of cultures, ethnicities and socio-cultural circumstances, many migrants can find themselves in vulnerable situations, marginalised by health systems,[8,9] and society alike.[10,11] There is a growing consensus around the need for academics and health systems to become more responsive to, and inclusive of refugee and migrant health concerns,[12] with the ultimate goal to collaboratively pursue research that is better centred around, and grounded in the needs of migrant communities. Indeed,

For peer review only - http://bmjopen.bmj.com/site/about/guidelines.xhtml

community engagement in public health interventions has already been shown to be effective when working with marginalised groups for a range of health outcomes, and can provide benefits to participants themselves, such as in improving health behaviours and participant self-efficacy.[13]

Despite the potential of participatory research, there are varied interpretations as to how to apply such approaches. Challenges exist in deciding who should be involved, and whether, involvement should extend beyond the target group (for example migrants), to other non-academic stakeholder groups such as community organisations and professionals.[3] There are also differing interpretations of the degree of participation required of individuals for research to be considered participatory rather than tokenistic, though it is suggested that unless involved individuals are partners or co-researchers throughout the entirety of a project, the work cannot be participatory.[3,14] Overall, it is widely agreed quality participation is characterised by non-academic stakeholders having opportunities to engage in research, make decisions, and perform leadership roles around such research,[5] empowering the public at the highest level and asserting their right to be involved in decision-making and to influence outcomes. Understanding the different approaches to participatory research and whether the core principles of participatory research are upheld is crucially important if good practice is to be identified.

We therefore did a systematic review to analyse participatory approaches used in health intervention research focused on migrants. We explored at what stages, and how migrants (or other non-academic stakeholders) were involved and analysed how this compares to principles of participatory research. We also looked for evidence of how such approaches had been evaluated by study authors, and how effective it was assessed to be.

For peer review only - http://bmjopen.bmj.com/site/about/guidelines.xhtml

Methods

We did a systematic review, following PRISMA guidelines, which is registered on the Open Science Framework (osf.io/2bnz5). The primary aim of this systematic review was to describe and analyse the participatory approaches utilised in health intervention research in which the intervention was aimed at benefitting migrant populations. We specifically sought to identify evidence on which stages of the research (inception, design, implementation, analysis, evaluation, dissemination) migrants (or other non-academic stakeholders) were involved in. We then analysed how they were involved and what influence they had over the research processes, comparing this to core principles of participatory research. Secondary outcomes were related to describing author evaluations of the impact utilising a participatory approach had on the research process, or of the participatory approach itself.

Inclusion and Exclusion criteria

We included peer-reviewed primary-research reporting on health interventions aimed at benefitting migrant populations that described using a participatory approach across the whole research process. Research which purported to use a participatory research approach through descriptors in their introduction and methods, or which utilised a recognised participatory approach such as community-based participatory research (CBPR), action research, or community-based collaborative action research, and specifically targeted migrants, was included in the review. We defined migrants as foreign-born individuals and considered a health intervention to be any initiative, tool, or programme that looked to improve health outcomes, including those related to mental health and health literacy.

Studies were excluded if they did not report on all stages of research into the health intervention: Inception, design, implementation, analysis, evaluation, dissemination.

For peer review only - http://bmjopen.bmj.com/site/about/guidelines.xhtml

As such, publications presenting interim results of studies which had not completed the full research cycle, as well as studies specifically focusing on only co-designing interventions were excluded. We took this approach so as not to unfairly penalise ongoing research in our analysis, nor co-designed research; we consider co-design to be one component of the broader participatory research paradigm and were most interested in how approaches manifest across the entirety of a research cycle.

Studies explicitly defining migrant status according to ethnic or ancestral background but not country of birth were excluded, as were papers where primary data were not reported (e.g., comments, editorials, letters, and reviews).

Search strategy

We searched the databases MEDLINE, Embase, Global Health and PsychINFO from inception – November 2019 utilising a Boolean search strategy with keywords and medical subheadings (MeSH) related to two major themes: migrants and participatory research. There were no geographic or language restrictions. An additional text file shows a representative search strategy (see Additional file 1). We identified additional studies through hand searching the bibliographies of publications included after full-text screening.

Study selection and quality assessment

Two reviewers duplicated the title and abstract screening and full-text screening (KR & SM-H), which was carried out using the web-based application Rayyan.[15] The reasons for excluding studies during full-text screening were recorded. Any discrepancies in screening decision between the two initial reviewers were mediated by a third reviewer (AC).

Data extraction and analysis

For peer review only - http://bmjopen.bmj.com/site/about/guidelines.xhtml

Studies which reported using participatory research approaches, and which reported on all stages of the research were extracted using a piloted form by KR and SH.

We extracted summary data on geographical location, the self-described participatory approach, specific target population, and aims of the research. Further data relating to the participatory approach of each study was extracted, categorised, and analysed.

We extracted data on the stages of the research in which migrants were involved. Where specific research stages did not involve migrants, but did involve other non-academic stakeholders this was recorded, sub-categorising these groups as community groups/third-sector organisations or professional services. We subsequently extracted data on the methods used to involve migrants (or other non-academic stakeholders) at each stage of the research (inception, design, implementation, analysis, evaluation, and dissemination). An additional file provides details of the summary extracted data (see Additional file 2).

Using this information, we analysed the overall participatory character of the studies from the perspective of inclusion and decision-making power of migrants specifically. For each stage of the research, we categorised these data in one of three ways (active, pseudo, indirect) within a framework developed with reference to the literature, particularly that relating to participatory research as a democratic process and being necessary to implement at all stage of the research.[3,5,14,16] The final framework and definitions were agreed by all co-authors (Table 1).

For peer review only - http://bmjopen.bmj.com/site/about/guidelines.xhtml

For peer review only

Table 1. Classifications and definitions of participatory research utilised in this systematic review.

Category of Participation		Definition
Active Participation		Evidence migrants (as the target population) were actively involved and wielded influence in decisions relating to the research (e.g., involved in voting on decisions, or being participants in steering committees which made the decisions).
Pseudo-participation	Proxy-participation	Evidence community groups/third-sector organisations and/or professional services were actively involved and wielded influence in decisions relating the research. However, migrants (as the target population) did not appear to be actively involved or were indirect participants at most.
	Indirect participation	Evidence migrants (as the target population) were subject to research activities (e.g., surveys, interviews), or partook in implementing/actioning research directions (e.g., delivering the designed intervention), but did not appear to have any active involvement or influence in decisions relating to this stage of the research. Furthermore, there was no evidence of community group/third-sector organisation involvement or influence.
No explicit evidence		There was no explicit evidence of the participation of migrants, community groups/third-sector organisations, or professional services regarding decision making processes related to the research stage.

For peer review only

For peer review only - http://bmjopen.bmj.com/site/about/guidelines.xhtml

Patient and Public Involvement

Members of our authorship team have past and current experience of working within third sector organisations. This experience helped to frame the research questions, and definitions used in the analysis. However, lay-patients and public specifically were not involved in this research.

Results

Screening results

Database searches returned 1793 results; a total of 292 duplicates were removed, and 1501 publications were retained for title and abstract screening. Of the 1501 remaining publications, 1357 were excluded during title and abstract screening, and 144 were retained for full-text screening. During full-text screening, 116 publications did not meet our criteria and were excluded, with the reasons for exclusion recorded (Fig 1.). Overall, 28 publications met the inclusion criteria and were included in this systematic review (Table 2.).

For peer review only - http://bmjopen.bmj.com/site/about/guidelines.xhtml

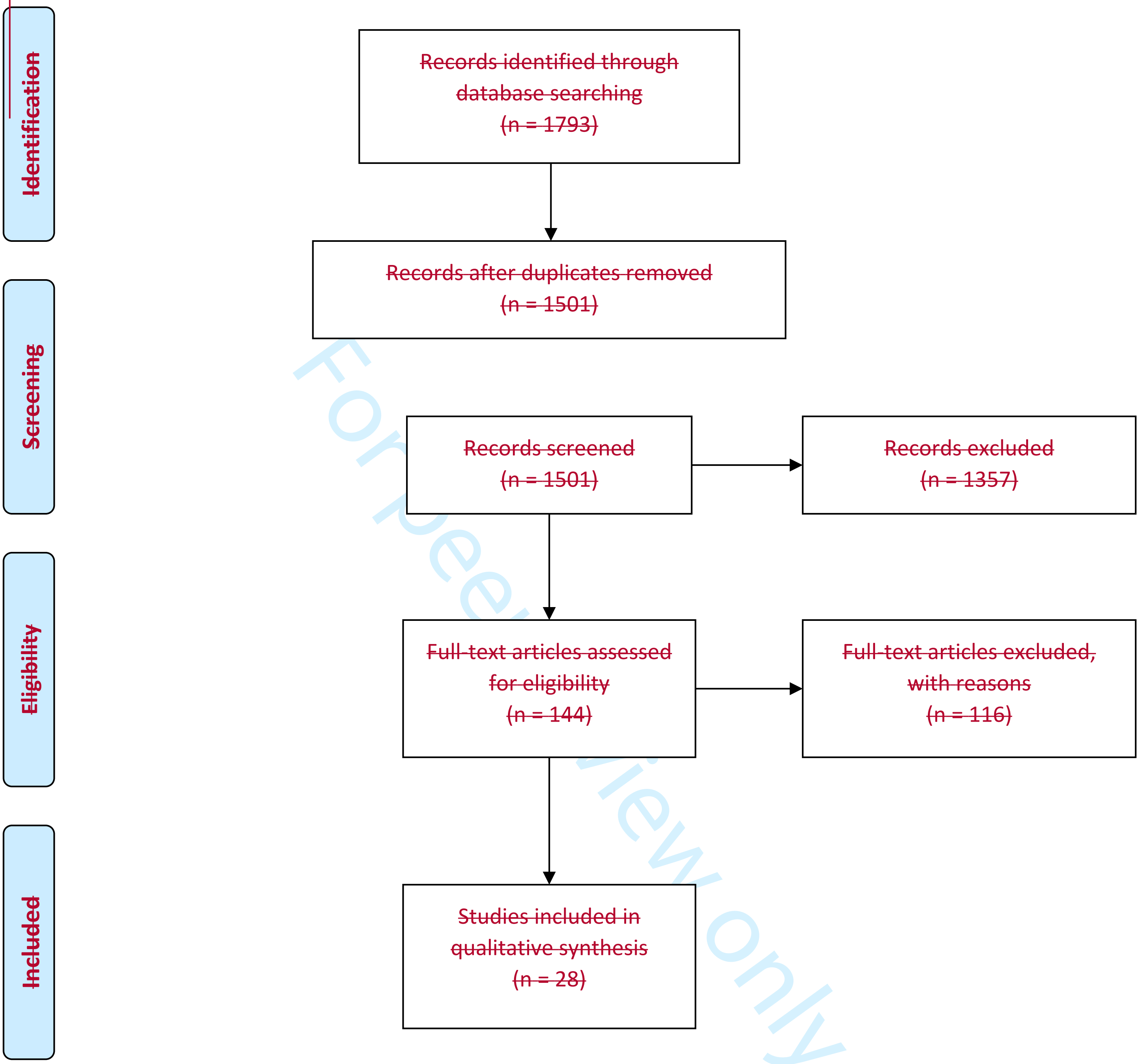

Figure 1. PRISMA flow-diagram of the study selection process

For peer review only

For peer review only - http://bmjopen.bmj.com/site/about/guidelines.xhtml

Citation	*Location*	*Self-described participatory approach*	*Specific Target population*	*Aim of the health intervention*
Afifi, Makhoul. 2011	*Lebanon*	*CBPR*	*Palestinian refugee youth*	*Mental health promotion*
Aitaoto, Braun. 2012	*United States*	*CBPR*	*Micronesian women*	*Cancer outreach/education*
Baird, Domian. 2015	*United States*	*Community-based collaborative-action research*	*Sudanese Refugee women*	*Addressing health challenges associated with relocation*
Barbee, Kobetz. 2010	*United States*	*CBPR*	*Haitian immigrant women*	*To assess the acceptability of HPV self-sampling with community health workers to detect cervical cancer*
Chesla, Chun. 2013	*United States*	*CBPR*	*Chinese-American Immigrants*	*To culturally adapt coping skills training for type-2 diabetes*
Evans, suggs. 2019	*United Kingdom*	*CBPR*	*African migrants*	*To promote HIV testing uptake*
Forst, Ahonen. 2013	*United States*	*CBPR*	*Hispanic construction workers*	*To increase awareness of workplace hazards and self-efficacy; expansion of worker centre agenda to include occupation health and safety*
Goodkind, Amer. 2017	*United States*	*CBPR*	*Afghan, Great lakes Region African, and Iraqi refugee adults*	*To address social determinants of health; to improve linkage to mental health services and retention in trauma-focused treatment*
Grigg-Saito, Och. 2008	*United*	*Community-Based*	*Cambodian*	*Strength-based outreach to eliminate*

For peer review only - http://bmjopen.bmj.com/site/about/guidelines.xhtml

	States	*Outreach*	*immigrants*	*cardiovascular disease and diabetes disparities*
Henderson, Slater.	*Canada*	*Action Research*	*Newly arrived migrants*	*To provide tailored nutritional information and support*
Jacquez, Vaughn. 2019	*United States*	*CBPR*	*Latino immigrants*	*Stress reduction*
Kaiser, Martinez. 2015	*United States*	*CBPR*	*Mexican immigrants*	*To provide obesity prevention education & outreach*
Kandula, Dave. 2016	*United States*	*CBPR*	*South Asian immigrant women*	*Exercise intervention for those at risk of diabetes*
Karasz, Raghavan. 2015	*United States*	*CBPR*	*Bangladeshi immigrant women*	*To provide and intervention tackling common mental disorders*
Kim, Koniak-Griffin. 2004	*United States*	*CBPR*	*Latino immigrants*	*To utilise Lay health advisors for cardiovascular health promotion*
Lam, Mcphee. 2003	*United States*	*CBPR*	*Vietnamese immigrants*	*To increase pap screening through education and outreach through lay health workers and media*
Li, Yeh. 2013	*United States*	*CBPR*	*Chinese-American immigrants*	*To prevent colorectal cancer through education and outreach*
Nilvarangkul, McCann. 2011	*Thailand*	*Action Research*	*Laotian migrants*	*Enhancement of a quality-of-life model*
Pinsker, Call. 2016	*United States*	*CBPR*	Somali youth	*To provide a culturally appropriate smoking cessation intervention*
Quandt, Grzywacz. 2014	*United States*	*CBPR*	*Latino immigrants*	*To provide Lay health promoter-led pesticide safety education*
Solorio, Norton-Shelpuk. 2014	*United States*	*CBPR*	*Latino immigrant MSM*	*To provide HIV prevention outreach for MSM*

For peer review only - http://bmjopen.bmj.com/site/about/guidelines.xhtml

Song, Han. 2010	*United States*	*CBPR*	*Korean-American immigrants*	*To translate current dietary guidelines into a culturally tailored nutrition program*
Suarez-Balcazar, Early. 2018	*United States*	*CBPR*	*Latino immigrants families with you with disabilities*	*To provide healthy lifestyle promotion*
Vaughn, Jacquez. 2019	*United States*	*CBPR*	*Latino immigrants*	*To reduce stress and increase coping skills*
Wieland, Nigon. 2019	*United States*	*CBPR*	*Foreign-born*	*To promote Tuberculosis screening*
Wieland, Njeru. 2017	*United States*	*CBPR*	*Immigrants and refugees with T2DM*	*To provide a digital story-telling intervention to improve management of type-2 diabetes among those affected*
Wieland, Wies. 2012	*United States*	*CBPR*	*Immigrant and refugee women*	*To provide a physical activity and nutrition program*
Williams, Ochsner. 2010	*United States*	*CBPR*	*Latino immigrants*	*Health and safety education in construction*

Table 2. Descriptive characteristic of studies included in this systematic review.

Study Characteristics

For peer review only

The research articles included in this systematic review were published between 2003 and 2019. Only 13 of the publications had any discernible dates relating to when the reported work was conducted, with these dates being between 2003 and 2018. The majority of the publications related to work carried out in the United States (24 out of 28); the remaining publications related to work carried out in Canada,[17] Lebanon,[18] Thailand,[19] and the United Kingdom.[20] The self-described approach taken by 24 of the 28 included studies was community-based participatory research (CBPR)[18,20-42]; the remaining four studies described using community-based collaborative-action research,[43] community-based outreach,[44] and action research.[17,19]. The dominant focus of the included studies was around education or outreach (Table 2), for example around cancer education,[21] or healthy lifestyles promotion;[37] five studies specifically mentioned including refugees (Table 2).[18,25,40,41,43]

Analysis of participation within included studies

In our analysis, participation varied substantially according to the stage of the research under scrutiny. Only two of the included studies reported explicit evidence of some degree of participation of at least one non-academic stakeholder groups across all research stages.[25,26] Overall, we extracted and categorised evidence of the participation of at least one non-academic stakeholder group in 22 studies during the inception;[17-19,21,22,25-30,32,34-36,38-44] 25 studies during the design;[17-19,21-35,37-42,44] 23 studies during implementation;[19-28,30,31,33-39,41-44] 4 studies during analysis;[25,26,35,39] 22 studies during evaluation;[17,19,20,23-33,35-38,40,42-44] and 4 studies during dissemination (Table 3.).[22,25,26,38]

For peer review only - http://bmjopen.bmj.com/site/about/guidelines.xhtml

Table 3. Analysis and categorisation of participatory character displayed across research stages within included studies

Citation	*Research Stage*					
	Inception	*Design*	*Implementation*	*Analysis*	*Evaluation*	*Dissemination*
Afifi, Makhoul. 2011	○	◆	X	X	X	X
Aitaoto, Braun. 2012	●	◆	◆	X	X	X
Baird, Domian. 2015	◆	X	○	X	○	X
Barbee, Kobetz. 2010	◆	◆	◆	X	X	◆
Chesla, Chun. 2013	X	◆	●	X	○	X
Evans, suggs. 2019	X	X	○	X	○	X
Forst, Ahonen. 2013	X	●	●	X	◆	X
Goodkind, Amer. 2017	◆	◆	◆	◆	◆	◆
Grigg-Saito, Och. 2008	◆	◆	◆	X	◆	X
Henderson, Slater.	●	○	X	X	○	X
Jacquez, Vaughn. 2019	◆	◆	◆	◆	◆	◆
Kaiser, Martinez. 2015	○	◆	○	X	○	X
Kandula, Dave. 2016	●	●	●	X	●	X
Karasz, Raghavan. 2015	●	◆	X	X	○	X
Kim, Koniak-Griffin. 2004	●	◆	◆	X	◆	X
Lam, Mcphee. 2003	X	◆	◆	X	◆	X
Li, Yeh. 2013	●	●	X	X	○	X
Nilvarangkul, McCann. 2011	◆	◆	●	X	●	X
Pinsker, Call. 2016	X	◆	◆	X	○	X
Quandt, Grzywacz. 2014	●	●	●	X	X	X
Solorio, Norton-Shelpuk. 2014	●	○	●	○	○	X
Song, Han. 2010	●	X	●	X	○	X
Suarez-Balcazar, Early. 2018	X	◆	◆	X	○	X
Vaughn, Jacquez. 2019	◆	◆	◆	X	○	◆

For peer review only - http://bmjopen.bmj.com/site/about/guidelines.xhtml

Wieland, Nigon. 2019	●	●	●	●	X	X
Wieland, Njeru. 2017	●	○	X	X	○	X
Wieland, Wies. 2012	●	○	●	X	X	X
Williams, Ochsner. 2010	●	◆	◆	X	○	X

◆ **Active participation:** Migrants (as the target population) were actively involved in decision-making processes and appeared to share power over decisions relating to this stage of the research.

● **Proxy-participation:** Evidence community groups/third-sector organisations and/or professional services were actively involved and wielded influence in decisions relating to this stage of the research. However, migrants (as the target population) did not appear to, and/or were indirectly participating.

○ **Indirect participation**: Evidence migrants (as the target population) were subject to research activities (e.g., surveys, interviews), or partook in implementing/actioning research directions (e.g., delivering the designed intervention), but did not appear to have any active involvement or influence in decisions relating to this stage of the research. Furthermore, there was no evidence of community group/third-sector organisation involvement or influence.

X **No explicit evidence:** There was no explicit evidence of the participation of migrants, community groups/third-sector organisations, or professional services regarding decision making processes related to this stage of the research stage.

For peer review only - http://bmjopen.bmj.com/site/about/guidelines.xhtml

However, there was greater variation and divergence in participatory research approaches when considering the degree of participation of migrants. In our analysis only 18 of the 28 included studies exhibit active participation of migrants (as the primary focus and target of the intervention) at any stage of the research process.[18,19,21-27,29-31,33,37,38,42-44] Of these 18 studies, only two display evidence of active participation of migrants at all stages of the research process.[25,26]

Across all 168 individual research stages analysed across the 28 studies, we recorded 46 instances of active participation of migrants; 30 instances of proxy participation; 24 instances of indirect participation; and 68 instances in which there was insufficient evidence to make a determination (Table 3). The active participation recorded also appears to be associated with the stage of the research. There were 7 instances of active participation during study inception;[19,22,26,38,43-45] 16 during design;[18,19,21-23,26,27,29-31,33,37,38,42,44,45] 10 during implementation;[21,22,26,30,31,33,37,38,42,44,45] 2 during analysis;[26,45] 6 during evaluation;[24,26,30,31,44,45] and 4 during dissemination.[22,26,38,45]

Benefits of participatory approaches to research

The benefit most often reported in utilising participatory approaches was the fact that interventions were better tailored to the target population through involving non-academic stakeholders.[18,21,23,28,33,37,38,42,46] This included two studies which spoke of the benefits of participatory research in facilitating interventions going beyond more immediately actionable cultural adaptations (such as language-adaptation and ethnically matched providers), to providing interventions that more deeply reflect community values and priorities.[23,38]

Partnering with non-academic stakeholders was cited as providing practical benefits during the research. One study reported how participatory approaches allowed for the modification of the research programme throughout conception, development, and implementation.[30] Multiple publications provided evidence on how iterative feedback from stakeholder during the studies could

For peer review only - http://bmjopen.bmj.com/site/about/guidelines.xhtml

aid partnerships, improving the recruitment of individuals to implement or take part in the intervention,[17,18,23,28,38,47] and dissemination.[23] One study also highlighted that partnerships were a feasible and appropriate means to support intervention implementation,[20] whilst one set of authors reported that partnerships with non-academics can ultimately strengthen research.[22]

Better relationships between the community and academics were cited as having the capability to enhance the familiarity and trust of partners. One study cited that increased trust had direct benefits to research, leading to more open and honest dialogue than in traditional research, improving the accuracy and findings of these activities.[43] Researchers becoming part of ongoing community relations was seen as positive, or a catalyst, acting as an impartial bridge between disparate community groups.[18] Long-lasting partnerships built over the course of research was cited as producing a capacity building element, increasing the health-related knowledge and resources of the community which academics partnered with.[24,31,39] Finally, partnerships catalysed a greater degree of understanding of a subject among communities, leading to increased self-determination and the ability to generate change of their own accord.[43]

Challenges of participatory approaches to research with migrants

Multiple studies highlighted the importance of balancing the culture and expectations of both researchers and partnered individuals to enact participatory research.[18,23,35] For example, one study reported that reaching equitability in the research process and working on level-terms with migrants directly conflicted with the cultural norms of some partners, who revere authority figures, and so would in normal circumstances defer to their judgement.[43] A further study highlighted challenges exist in bringing together differing stakeholders, with varied views and experiences. In these situations it was suggested there is no “one size fits all” approach, and that processes must be adapted to individual partner groups.[31] Noting varied perspective, one study highlights the challenge

For peer review only - http://bmjopen.bmj.com/site/about/guidelines.xhtml

that the divergent perspectives of what is most salient and important to address amongst partners can present a challenge.[25]

The challenge (and importance) of building rapport and addressing mistrust,[18,19,44] or even research fatigue amongst some partner groups,[18] was also evident within publications. One set of authors, spoke not just of the need for non-academic partners to trust researchers, but how It is also essential for researchers to trust wider stakeholder partners, and prioritise the collaborative and democratic aim of participatory methods; this was perceived as challenging as it may shift the power dynamic and locus of control in the research away from the academics.[34] Even when partnerships overcome challenges of culture, expectations and trust, there remain other practical challenges to operationalising these partnerships.[29,33]

Challenges in ensuring equitability in research understanding, and balancing the participatory nature of a project, with the standards expected by the wider scientific community were also highlighted.[18,31] Furthermore, one study cited the difficulty of navigating acknowledgement and authorship of non-academics in published materials.[23] The lack of recognition of the requirements of participatory research in traditional academic circles is also cited as a challenge, with one set of authors stated the need for managerial, institutional, and funder-level buy-in and commitment regarding participatory research.[18] Similarly, institutional review limited the participation in at least one study, preventing non-academic stakeholders participating in data collection and analysis.[31]

Other practical challenges to operationalising partnerships included effective, timely communication,[19,32] and the challenge of partnering with communities in which the dominant language of the researchers and migrant communities differ.[35,43] Finally, the iterative and tailored nature of the interventions produced may also impact on the generalisability of findings,[37] whilst some work could seemingly omit or contradict research evidence due to localising the intervention.[40]

For peer review only - http://bmjopen.bmj.com/site/about/guidelines.xhtml

Discussion

To our knowledge this is the first systematically review to robustly measure the application of participatory approaches and principles to health intervention research with migrants. Whilst specifically focusing on research with migrants, many of the findings, and the framework discussed are likely to provide insight to all practitioners of participatory research. The 28 studies included reported on a variety of health interventions, predominantly revolving around outreach and education. Our analysis shows that 18 of the 28 included studies actively involving migrants themselves, but only two studies actively involved migrants during all stages of the research process. The remaining studies either provide insufficient evidence to determine the participatory approach utilised, or were characterised by pseudo-participation, in which community groups/third-sector organisations were directly involved (proxy-participation), or migrants were only involved through being subjects in research activities (indirect-participation).

The approaches taken to participatory research in the studies included in this systematic review varied. The examples that represent the most participatory approach, according to our analysis, were characterised by co-operation and power-distribution (Table 4). The difference between active participatory approaches, and those we characterised as pseudo-participation, appear subtle when viewed from a research-centric perspective but are stark when considering a participatory perspective. Firstly, indirect participation, in which migrants are involved in activities such as surveys or interviews designed to inform health interventions may represent a perfectly suitable means to guide development and build evidence, but do little to distribute power in a participatory manner. The risk that research is framed as participatory whilst failing to develop equitable partnerships has previously been highlighted, and still appears to persist.[3,48] Secondly, proxy-participation, which may do more to uphold principles of participatory research, may still be at risk of not equitably involving the actual target population. Uncertainty persists around how to best involve non-academic stakeholders, and ensure those that are involved are representative of the population of interest.[49]

For peer review only - http://bmjopen.bmj.com/site/about/guidelines.xhtml

Whilst community-groups and/or professional services involvement may at times be the only, or most readily available way to represent the population of interest (due to difficulties (perceived or otherwise) in accessing, or providing access to migrants), they cannot be assumed to be representative of them. Previous research has shown that health-service users can identify different needs to service-providers.[50] Furthermore, whilst overall understandings of involvement processes may align, service-providers may place different values on some aspects of involvement.[51] Therefore, proxy-participation could conceivably skew participatory research away from being centred on migrants' needs.

For peer review only - http://bmjopen.bmj.com/site/about/guidelines.xhtml

439

440

441 **Table 4.** Descriptive tabulation of two studies classified as displaying active participation throughout

442 all stage of the health intervention research with migrants.

		Research Stage					
		Inception	Design	Implementation	Analysis	Evaluation	Dissemination
Studies	Goodkind, Amer. 2017	Study conceived from previous relations with community groups. The present study was guided by refugees, and community service providers.	The community was involved in designing interview protocols and participant recruitment procedures.	All interpreters and interviewers were refugees; procedures had been agreed during inception and design.	Refugees were involved in analysis and were actively involved in setting the agenda for what evidence was meaningful.	Refugees and community involved in discussions to evaluate the process; indication that the decision a community intervention paradigm be adopted appears to have been adopted and championed by researchers as a result.	Refugees were involved in the dissemination and are co-authors of the research publication.
	Jacquez, Vaughn. 2019	Manifested from a previous relationship with latinos unidos por la salud to promote health and	co-researchers worked with academic partners to identify primary outcomes	Co-researchers recruited and worked with participants to identify strategies for stress reduction.	co-researchers and academic partners identified the primary outcomes.	Academic and community partners shared decision-making in all aspects of the	Academic and community partners shared decision-making in all aspects of the research

For peer review only - http://bmjopen.bmj.com/site/about/guidelines.xhtml

		healthcare for the local latino community; co-researechers in this project were drawn from the local community.	, and helped decide that health-worker delivered strategies were the preferred intervention option			research process, including evaluation.	process, including dissemination.

We found that involvement across all stages of the research was limited, and least prevalent in interpreting or disseminating research findings. Our findings are supported by and corroborate existing research, including two systematic reviews carried out to analyse how CBPR enhanced clinical trials for racial and ethnic minority groups,[52] and what it means to conduct participatory research with indigenous peoples.[53] These reviews found similar trends, including that participation varies substantially, and that research that is expressly participatory is often limited with key methodological challenges, around collecting, interpreting, and disseminating research. However, these reviews align with this research highlighting benefits of participatory research for the recruitment and retention of trial participants.

Upholding the core principles of participatory research, in this instance, democratising research and power-sharing, is particularly pertinent to partnering with migrants. Participatory research origins are firmly rooted in increasing social justice, and the promotion of doing research with, not on or about individuals and communities, particularly those that are disadvantaged.[48] Migrant communities are often marginalised within recipient countries,[10,11] and by local health systems.[8,9] Not only is it inappropriate for research to perpetuate or deepen any marginalisation through failing to include migrants' voices, insights, and skills, but there are benefits to the proper utilisation of

For peer review only

participatory approaches to the overall research process. Included studies provide evidence of the benefits to participant recruitment, implementation, and dissemination. Researchers also highlighted that the iterative nature of participatory research allows more effective tailoring of work to the needs of migrants, through learning from and embedding migrant partners knowledge and experience into research. Whilst studies we identify predominantly focus on community outreach and education within health research, participatory research could be better utilised across all disciplines. Similar methodology could be employed to better design pharmaceuticals, or on a larger scales, procedures and systems at a governance level.

Effectively partner with migrants requires specific strategies, to address the challenges identified in this review. Some of these strategies include: early participatory involvement to guide research priorities, methodological approaches, and strategies to manage ongoing relations; translating and back-translating materials; giving reassurance as to the confidentiality of involvement, and respecting decisions around reporting (particularly as some partners may be undocumented migrants or have precarious legal status); utilising a variety of outreach and recruitment outlets, such as NGOs and religious groups trusted by migrants; and identifying opportunities for bidirectional benefits in the research, and capacity building to facilitate collaborative and democratic participation. Those partnering with migrants must demonstrate flexibility to negotiate potential power divides, and acknowledge and be considerate of residual mistrust that may exist among communities, even after researcher-community relationships appear well established.[54] The challenges and extra consideration highlighted by this review must not be underestimated, whilst from a research perspective, more still needs to be done to assess the impact of participatory approaches on overall research processes and output. However, if research is to become more democratic, patient-centred, and representative of the populations impacted by its work, traditional scientific approaches are likely to be inadequate, with academic researchers holding the majority of power over research.[55]

For peer review only - http://bmjopen.bmj.com/site/about/guidelines.xhtml

Greater adoption of consistent and transparent reporting of participatory research is needed, to support the need for more critical analysis of involvement and participatory research.[56] Whilst guidelines have been developed,[57] they have not been widely adopted, with no material improvement in the reporting quality of published studies seen within some fields as a result of the their publication, which could be attributed to a lack of awareness of the guidelines existence.[58] Tensions exist as to whether participatory research should be conceptualised and evaluated similarly to traditional research[56,59] However, we believe reporting can be sympathetic of the need to evidence impact of methods and processes on research. We propose the plain reporting of who was involved in each element of the research, why these individuals were involved; how they were involved; and who ultimately controlled decisions relating to each research stages should be completed for all research involving non-academic stakeholders. The development of guidelines to support this reporting would simultaneously allow a more complete assessment of how participatory research impacted research outcomes, and greater reflection and evaluation of the participatory approaches employed.[59]

Our review has several limitations. Firstly, we acknowledge the taxonomy of terminology around participatory research is not standardised and terms are used inconsistently. As such, the categorisations we have introduced and used in this review may be defined differently by others.. Furthermore, whether the included studies categorised in our analysis exhibit greater or lesser participation is potentially immaterial to the quality of the research carried out, or the impact of the final intervention. This review represents our attempt to cast a critical lens over how the principles of participatory research are applied in practice. Our conceptual focus on migrants' involvement is therefore not intended to denigrate the efforts of third-sector organisations or professionals services, whose involvement we may have classified as proxy-participation. Fundamentally we believe the examined studies are inherently more participatory than traditional research

For peer review only - http://bmjopen.bmj.com/site/about/guidelines.xhtml

endeavours, for having even considered and attempted to involve non-academic stakeholders. We recognise the challenges associated with participatory research, and as stated, hold no assumptions about the extent of participation and its' association with beneficial outcomes for target populations.

For peer review only - http://bmjopen.bmj.com/site/about/guidelines.xhtml

Conclusion

In conclusion, participatory approaches to research for health interventions aimed at migrants are insufficiently applied and reported. We provide evidence that the application of approaches does not fully embody core principles of participatory research, particularly relating to providing decision-making power to individuals ultimately affected by the research. Those who wish to engage in participatory research must consider the approach they take, and whether it is sufficient to achieve high-quality participation, not just high-quality research. Crucially, guidelines for reporting of participatory research methods must be introduced. This will enable all parties, from academics to communities to better assess the participatory nature of individual research projects, and is an important pre-requisite to exploring the overall impact of participatory research which is inadequately understood.

For peer review only

Declarations

Ethics approval and consent to participate

This research is a systematic review of existing literature. As such, no human participants were involved.

Consent for publication

Not applicable

Availability of data and materials

All data generated or analysed during this study are included in this published article.

Competing interests

The authors declare that they have no competing interests.

Funding Statement.

This work has been supported by the European Society of Clinical Microbiology and Infectious Diseases (ESCMID) Study Group for Infections in Travellers and Migrants (ESGITM). KR is funded by the Rosetrees Trust (M775). SH, AFC, and LG are funded by the NIHR (NIHR Advanced Fellowship NIHR300072) and SH and AFC are funded by the Academy of Medical Sciences (SBF005\1111). AD is funded by the MRC (MR/N013638/1).

Author Contributions

The study was conceptualised by KR, LN, SH and JSF with input on finalising the study question and protocol from SM-H and AC. Searches were carried out by KR, and screening done by KR, SM-H and AC. Data extraction and analysis was done by KR, AC and SH. Interpretation of the results and drafting of the manuscript was done primarily by KR with input from all authors. All authors commented upon and approved the final manuscript.

Acknowledgements

Not applicable

Authors' information

Not applicable

For peer review only

For peer review only - http://bmjopen.bmj.com/site/about/guidelines.xhtml

Additional Files

Additional file 1.docx – Representative search strategy and terms used in the systematic review. This additional file contains a table displaying a representative search strategy used to source articles through database searching.

Additional file 2.docx – Summary extracted data from studies included in this systematic review. This additional files contains a table displaying the summary extracted data obtained and analysed in this systematic review.

Figure Legends:

Figure 1. PRISMA flow-diagram of the study selection process

For peer review only - http://bmjopen.bmj.com/site/about/guidelines.xhtml

References

1 Control., E. C. f. D. P. a. Guidance on community engagement for public health events caused by communicable disease threats in the EU/EEA. (ECDC; 2020, Stockholm, 2020).

2 Research., N. I. f. H. UK Standards for Public Involvement. (2019).

3 Bergold, J. & Thomes, S. Participatory Research Methods: A Methodological Approach in Motion. *Forum Qualitative Sozialforschung / Forum: Qualitative Social Research* **13**, doi:http://nbn-resolving.de/urn:nbn:de:0114-fqs1201302. (2012).

4 Position Paper 1: What is Participatory Health Research? , (International Collaboration for Participatory Health Research, Berlin, 2013).

5 Springett, J., Katkey, K., Kongats, K., Zulla, R. & Wilkins, E. Conceptualizing Quality in Participatory Health Research: A Phenomenographic Inquiry. *Forum Qualitative Sozialforschung / Forum: Qualitative Social Research* **17**, doi:http://nbn-resolving.de/urn:nbn:de:0114-fqs1602274. (2016).

6 Crawshaw, A. F. *et al.* What must be done to tackle vaccine hesitancy and barriers to COVID-19 vaccination in migrants? *Journal of Travel Medicine*, doi:10.1093/jtm/taab048 (2021).

7 Cargo, M. & Mercer, S. The Value and Challenges of Participatory Research: Strengthening its Practice. *Annual Review of Public Health* **29**, 325-350 (2008).

8 Legido-Quigley, H. *et al.* Healthcare is not universal if undocumented migrants are excluded. doi:10.1136/bmj.l4160 (2019).

9 O'Donnel, c. Health Care Access for Migrants in Europe - Oxford Research Encyclopedia of Global Public Health. *Global Public Health*, doi:10.1093/acrefore/9780190632366.013.6 (2018).

10 Thorbjørnsrud, K. & Figenschou, T. U. Do Marginalized Sources Matter? *http://dx.doi.org/10.1080/1461670X.2014.987549*, doi:10.1080/1461670X.2014.987549 (2014).

For peer review only - http://bmjopen.bmj.com/site/about/guidelines.xhtml

11 Migrants perspectives on Brexit and UK immigration policies. (Migrants Rights Network, 2017).

12 Health promotion for improved refugee and migrant health: Technical guidance. (World Health Organization, Geneva, 2017).

13 O'Mara-Eves, A. *et al.* The effectiveness of community engagement in public health interventions for disadvantaged groups: a meta-analysis. *BMC Public Health* **15**, 1-23, doi:doi:10.1186/s12889-015-1352-y (2015).

14 Cook, T. Where Participatory Approaches Meet Pragmatism in Funded (Health) Research: The Challenge of Finding Meaningful Spaces. *Forum Qualitative Sozialforschung / Forum: Qualitative Social Research* **13**, doi:http://dx.doi.org/10.17169/fqs-13.1.1783 (2012).

15 Ouzzani, M., Hammady, H., Fedorowicz, Z. & Elmagarmid, A. Rayyan—a web and mobile app for systematic reviews. *Systematic Reviews* **5**, 1-10, doi:doi:10.1186/s13643-016-0384-4 (2016).

16 *Core Values, Ethics, Spectrum - The 3 Pillars of Public Participation*, <https://www.iap2.org/page/pillars> (2020).

17 Henderson, A. & Slater, J. Growing Roots: A Newcomer Nutrition Program Designed Using Action Research Methods. *Ecology of Food and Nutrition* **58**, 430-455, doi:10.1080/03670244.2019.1636792. (2019).

18 Afifi, R. A., Makhoul, J., El Hajj, T. & Nakkash, R. T. Developing a logic model for youth mental health: participatory research with a refugee community in Beirut. *Health Policy Plan* **26**, 508-517, doi:10.1093/heapol/czr001 (2011).

19 Nilvarangkul, K., McCann, T. V., Rungreangkulkij, S. & Wongprom, J. Enhancing a health-related quality-of-life model for Laotian migrant workers in Thailand. *Qual Health Res* **21**, 312-323, doi:10.1177/1049732310376635 (2011).

For peer review only

For peer review only - http://bmjopen.bmj.com/site/about/guidelines.xhtml

20 Evans, C. *et al.* Mobile phone messaging to promote uptake of HIV testing among migrant African communities in the UK:. https://doi.org/10.1177/0017896918785928, doi:10.1177_0017896918785928 (2018).

21 Aitaoto, N., Braun, K. L., Estrella, J., Epeluk, A. & Tsark, J. Design and results of a culturally tailored cancer outreach project by and for Micronesian women. *Prev Chronic Dis* **9**, E82, doi:10.5888/pcd9.100262 (2012).

22 Barbee, L. *et al.* Assessing the acceptability of self-sampling for HPV among Haitian immigrant women: CBPR in action. *Cancer Causes Control* **21**, 421-431, doi:10.1007/s10552-009-9474-0 (2010).

23 Chesla, C. A. *et al.* Testing the efficacy of culturally adapted coping skills training for Chinese American immigrants with type 2 diabetes using community-based participatory research. *Res Nurs Health* **36**, 359-372, doi:10.1002/nur.21543 (2013).

24 Forst, L. *et al.* More than training: Community-based participatory research to reduce injuries among hispanic construction workers. *Am J Ind Med* **56**, 827-837, doi:10.1002/ajim.22187 (2013).

25 Goodkind, J. R. *et al.* Challenges and Innovations in a Community-Based Participatory Randomized Controlled Trial. *Health Education & Behavior* **44**, 123-130, doi:10.1177/1090198116639243 (2017).

26 Jacquez, F., Vaughn, L. M. & Suarez-Cano, G. Implementation of a Stress Intervention with Latino Immigrants in a Non-traditional Migration City. *Journal of Immigrant and Minority Health* **21**, 372-382, doi:doi:10.1007/s10903-018-0732-7 (2018).

27 Kaiser, L. *et al.* Adaptation of a culturally relevant nutrition and physical activity program for low-income, Mexican-origin parents with young children. *Prev Chronic Dis* **12**, E72, doi:10.5888/pcd12.140591 (2015).

28 Kandula, N. R. *et al.* An Exercise Intervention for South Asian Mothers with Risk Factors for Diabetes. *Transl J Am Coll Sports Med* **1**, 52-59 (2016).

For peer review only

For peer review only - http://bmjopen.bmj.com/site/about/guidelines.xhtml

29 Karasz, A. *et al.* ASHA: Using Participatory Methods to Develop an Asset-building Mental Health Intervention for Bangladeshi Immigrant Women. *Progress in Community Health Partnerships: Research, Education, and Action* **9**, 501-512, doi:https://doi.org/10.1353/cpr.2015.0080 (2015).

30 Kim, S., Koniak-Griffin, D., Flaskerud, J. H. & Guarnero, P. A. The impact of lay health advisors on cardiovascular health promotion: using a community-based participatory approach. *J Cardiovasc Nurs* **19**, 192-199, doi:10.1097/00005082-200405000-00008 (2004).

31 Lam, T. K. *et al.* Encouraging Vietnamese-American Women to Obtain Pap Tests Through Lay Health Worker Outreach and Media Education. *J Gen Intern Med* **18**, 516-524, doi:10.1046/j.1525-1497.2003.21043.x (2003).

32 Li, M., Yeh, Y.-L., Sun, H., Chang, B. & Chen, L.-s. Community-Based Participatory Research: a Family Health History-Based Colorectal Cancer Prevention Program Among Chinese Americans. *Journal of Cancer Education : the Official Journal of the American Association for Cancer Education*, doi:10.1007/s13187-019-1486-1 (2019).

33 Pinsker, E. *et al.* The Development of Culturally Appropriate Tobacco Prevention Videos Targeted Toward Somali Youth. *Progress in Community Health Partnerships: Research, Education, and Action* **11**, doi:https://doi.org/10.1353/cpr.2017.0017 (2017).

34 Quandt, S. A. *et al.* Evaluating the effectiveness of a lay health promoter-led, community-based participatory pesticide safety intervention with farmworker families. *Health Promot Pract* **14**, 425-432, doi:10.1177/1524839912459652 (2013).

35 Solorio, R., Norton-Shelpuk, P., Forehand, M., Martinez, M. & Aguirre, J. HIV Prevention Messages Targeting Young Latino Immigrant MSM. *AIDS Res Treat* **2014**, 353092, doi:10.1155/2014/353092 (2014).

For peer review only

36 Song, H. J. *et al.* Translating current dietary guidelines into a culturally tailored nutrition education program for Korean American immigrants with type 2 diabetes. *Diabetes Educ* **36**, 752-761, doi:10.1177/0145721710376328 (2010).

37 Suarez-Balcazar, Y. *et al.* Community-based participatory research to promote healthy lifestyles among Latino immigrant families with youth with disabilities. https://doi.org/10.1080/11038128.2018.1502348, doi:1502348 (2018).

38 Vaughn, L., Jacquez, F. & Suarez-Cano, G. Developing and Implementing a Stress and Coping Intervention in Partnership With Latino Immigrant Coresearchers. *Translational Issues in Psychological Science* **5**, 62-77, doi:10.1037/tps0000172 (2019).

39 Wieland, M. L. *et al.* Sustainability of a Tuberculosis Screening Program at an Adult Education Center Through Community-Based Participatory Research. *J Public Health Manag Pract* **25**, 602-605, doi:10.1097/phh.0000000000000851 (2019).

40 Wieland, M. L. *et al.* Pilot Feasibility Study of A Digital Storytelling Intervention for Immigrant and Refugee Adults with Diabetes. *Diabetes Educ* **43**, 349-359, doi:10.1177/0145721717713317 (2017).

41 Wieland, M. L. *et al.* Physical activity and nutrition among immigrant and refugee women: a community-based participatory research approach. *Womens Health Issues* **22**, 225-232, doi:10.1016/j.whi.2011.10.002. (2012).

42 Williams, Q., Jr., Ochsner, M., Marshall, E., Kimmel, L. & Martino, C. The impact of a peer-led participatory health and safety training program for Latino day laborers in construction. *J Safety Res* **41**, 253-261, doi:10.1016/j.jsr.2010.02.009 (2010).

43 Baird, M. B. *et al.* Creating a Bridge of Understanding between Two Worlds: Community-Based Collaborative-Action Research with Sudanese Refugee Women. *Public Health Nurs* **32**, 388-396, doi:10.1111/phn.12172 (2015).

For peer review only - http://bmjopen.bmj.com/site/about/guidelines.xhtml

44 Grigg-Saito, D., Och, S., Liang, S., Toof, R. & Silka, L. Building on the strengths of a Cambodian refugee community through community-based outreach. *Health Promot Pract* **9**, 415-425, doi:10.1177/1524839906292176 (2008).

45 Goodkind, J. R. *et al.* Challenges and Innovations in a Community-Based Participatory Randomized Controlled Trial. *https://doi.org/10.1177/1090198116639243*, doi:10.1177_1090198116639243 (2016).

46 Coffman, M. J. *et al.* Using CBPR to Decrease Health Disparities in a Suburban Latino Neighborhood. *Hisp Health Care Int* **15**, 121-129, doi:10.1177/1540415317727569 (2017).

47 Documet, P. I. *et al.* A Male Promotores Network for Latinos: Process Evaluation From a Community-Based Participatory Project. *Health Promot Pract* **17**, 332-342, doi:10.1177/1524839915609059 (2016).

48 Macaulay, A. C. Participatory research: What is the history? Has the purpose changed? *Family Practice* **34**, 256-258, doi:https://doi.org/10.1093/fampra/cmw117 (2017).

49 Lander, J., Langhof, H. & Dierks, M.-L. Involving patients and the public in medical and health care research studies: An exploratory survey on participant recruiting and representativeness from the perspective of study authors. *PLoS One* **14**, doi:10.1371/journal.pone.0204187 (2019).

50 Edwards, L. *et al.* What matters most to patients about primary healthcare: mixed-methods patient priority setting exercises within the PREFeR (PRioritiEs For Research) project. doi:10.1136/bmjopen-2018-025954 (2019).

51 Rise, M. B. *et al.* Same description, different values. How service users and providers define patient and public involvement in health care. *Health Expect.* **16**, 266-276, doi:10.1111/j.1369-7625.2011.00713.x (2013).

52 De Las Nueces, D., Hacker, K., DiGirolamo, A. & Hicks, L. S. A Systematic Review of Community-Based Participatory Research toEnhance Clinical Trials in Racial andEthnic

For peer review only

For peer review only - http://bmjopen.bmj.com/site/about/guidelines.xhtml

Minority Groups. *Health Services Research* **47**, 1363-1386, doi:https://doi.org/10.1111/j.1475-6773.2012.01386.x (2012).

53 Dadich, A., Moore, L. & Eapen, V. What does it mean to conduct participatory research with Indigenous peoples? A lexical review. *BMC Public Health* **19**, doi:https://doi.org/10.1186/s12889-019-7494-6 (2019).

54 Ahmed, A., Vandrevala, T., Hendy, J., Kelly, C. & Ala, A. An examination of how to engage migrants in the research process: building trust through an 'insider' perspective. *https://doi.org/10.1080/13557858.2019.1685651*, doi:CETH-2019-0030.R2 (2019).

55 Green, G. Power to the people: To what extent has public involvement in applied health research achieved this? *Research Involvement and Engagement* **2**, 1-13, doi:doi:10.1186/s40900-016-0042-y (2016).

56 Russel, J., Fudge, N. & Greenhalgh, T. The impact of public involvement in health research: what are we measuring? Why are we measuring it? Should we stop measuring it? *Research Involvement and Engagement* **6**, doi:https://doi.org/10.1186/s40900-020-00239-w (2020).

57 Smith, L., Rosenzweig, L. & Schmidt, M. Best Practices in the Reporting of Participatory Action Research: Embracing Both the Forest and the Trees 1Ψ7:. *http://dx.doi.org/10.1177/0011000010376416*, doi:10.1177_0011000010376416 (2010).

58 Kato, D. *et al.* Reporting Guidelines for Community-Based Participatory Research Did Not Improve the Reporting Quality of Published Studies: A Systematic Review of Studies on Smoking Cessation. *Int J Environ Res Public Health* **17**, doi:10.3390/ijerph17113898 (2020).

59 Staley, K. & Barron, D. Learning as an outcome of involvement in research: what are the implications for practice, reporting and evaluation? *Research Involvement and Engagement* **5**, doi:https://doi.org/10.1186/s40900-019-0147-1 (2019).

For peer review only - http://bmjopen.bmj.com/site/about/guidelines.xhtml

For peer review only

Appendix 1. Representative search terms/strategy used

**Migrant identifier terms**
Migrant* *or*
Migrat* *or*
Refugee* *or*
Foreign* *or*
Non-native* *or*
immigrant* *or*
emigra* *or*
oversea* *or*
Foreign students
**Participatory research terms**
Co-design* *or*
Co-prod* *or*
Co-creat* *or*
Collab* design* *or*
Collab* prod* *or*
Collab* create* *or*
Community design* *or*
Community prod* *or*
Community create* *or*
Community-based participatory research *or*
Action research *or*
Participatory action research *or*
Participatory adj4 research *or*
Patient involvement *or*
Patient and public involvement

For peer review only

Appendix 1. Summary of extracted data relating to involvement and engagement of non-academic stakeholders, and their influence over given research stages.

1. **Afifi, Makhoul et al. 2011**

   Aim & Target population: *Development of a mental health promotion intervention for Palestinian refugee youths.*

   Inception: *Based on a previous survey of the target population which had been designed with community input*
   Design: *Interview and survey of target population; formation of a community youth council with youth council representatives which agreed internal rules and implemented democratic voting re: design decisions*
   Implementation: *No explicit evidence of non-academic stakeholder involvement, engagement, or influence.*
   Analysis: *No explicit evidence of non-academic stakeholder involvement, engagement, or influence.*
   Evaluation: *No explicit evidence of non-academic stakeholder involvement, engagement, or influence.*
   Dissemination: *No explicit evidence of non-academic stakeholder involvement, engagement, or influence.*

2. **Aitaoto, Braun et al. 2012**

   Aim & Target population: *Implementation of a cancer outreach programme for Micronesian women*

   Inception: *Community group representing the target population approached researchers to highlight this topic*
   Design: *Focus groups with the target population; champions from the target population were involved in literature review and aided in designing the outreach programme*
   Implementation: *Target population delivered the programme as lay-educators, a decision clearly defined in the design stage by working with the champions*
   Analysis: *No explicit evidence of non-academic stakeholder involvement, engagement, or influence.*
   Evaluation: *No explicit evidence of non-academic stakeholder involvement, engagement, or influence; reported that the research did not ask the target population about perceptions of the intervention*
   Dissemination: *No explicit evidence of non-academic stakeholder involvement, engagement, or influence.*

3. **Baird, Domian et al. 2015**

   Aim & Target population: *To address the health challenges associated with relocation among Sudanese refugees*

   Inception: *Collaborative inception between Sudanese community and researchers manifesting from previous research shared to community members, who then formed a committee which worked with the researchers; Whilst topics for intervention were collaboratively developed between researchers and the target population*
   Design: *research team developed educational seminars on intervention topics.*
   Implementation: *Educational seminars were delivered by the research team; target population were involved in advertising and recruiting other participants.*
   Analysis: *No explicit evidence of non-academic stakeholder involvement, engagement, or influence.*
   Evaluation: *Focus group discussion to evaluate the process, discuss overall findings, and plan future relationships.*

For peer review only

Dissemination: *No explicit evidence of non-academic stakeholder involvement, engagement, or influence.*

4. Barbee, Kobetz et al. 2010

Aim & Target population: *To assess the acceptability of HPV self-sampling with community health workers, to detect cervical cancer among Haitian immigrant women.*

Inception: *Conceived by a campus-community partnership; the partnership is formed of community leaders and academics; community leaders are largely responsible for identifying research needs*
Design: *Community partners responsible for defining research focus, recruitment strategies, and data collection methods; academic partners write grants applications and obtain institutional approvals*
Implementation: *Community and academic partners contribute collectively to study implementation*
Analysis: *No explicit evidence of non-academic stakeholder involvement, engagement, or influence.*
Evaluation: *No explicit evidence of non-academic stakeholder involvement, engagement, or influence.*
Dissemination: *Community and academic partners contribute collectively to study dissemination*

5. Chesla, Chun. 2013

Aim & Target population: *Cultural adaptation of coping skills training for Chinese-American immigrants with type 2 diabetes.*

Inception: *No explicit evidence of non-academic stakeholder involvement, or influence; there is mention of a CBPR work group, and a community advisory board, but no indication these groups were involved in the inception.*
Design: *A CBPR work group composed of 10 paid staff from community agencies and academic institutions. The work group culturally adapted protocols based on clinical guidelines, clinical knowledge, and previous studies.; A community advisory board met semi-annually and provided oversight on all aspect of the CBPR activities.*
Implementation: *Work group members who were bilingual with degree level qualification in social work implemented the intervention.*
Analysis: *No explicit evidence of non-academic stakeholder involvement, engagement, or influence.*
Evaluation: *Evaluated through research/survey with participants.*
Dissemination: *No explicit evidence of non-academic stakeholder involvement, engagement, or influence.*

6. Evans, Suggs et al. 2019

Aim & Target population: *Promoting HIV testing uptake amongst African migrant communities*

Inception: *No explicit evidence of non-academic stakeholder involvement, engagement, or influence.*
Design: *The design was guided by previous formative research conducted by the researchers*
Implementation: *Participants were invited by community researchers that represented diverse African communities and genders.*
Analysis: *No explicit evidence of non-academic stakeholder involvement, engagement, or influence.*
Evaluation: *The target population provided feedback through surveys*
Dissemination: *No explicit evidence of non-academic stakeholder involvement, engagement, or influence.*

For peer review only

7. Forst, Ahonen et al. 2013

Aim & Target population: Increase awareness of workplace hazards and self-efficacy among foreign-born Hispanic construction workers; and expand worker centre agenda to include occupational health & safety.

Inception: *No explicit evidence of non-academic stakeholder involvement, engagement, or influence.*
Design: *Worker centres (WC) and academics partnered to set joint goals and specific responsibilities: WC would recruit peer-educators and workers to the training, partner with implementation and evaluation; university partners assumed responsibility for subject protection, evaluation, design and implementation.*
Implementation: *Worker centre staff were responsible for recruiting peer-educators to deliver the curriculum; university partners assumed responsibility for implementation*
Analysis: *No explicit evidence of non-academic stakeholder involvement, engagement, or influence.*
Evaluation: *Iterative evaluation of the intervention with peer-educators in a democratic manner; peer-educators were able to influence study design*
Dissemination: *No explicit evidence of non-academic stakeholder involvement, engagement, or influence.*

8. Goodkind, Amer et al. 2017

Aim & Target population: **to address social determinants of health, improve links to mental health services, and improve retention in trauma-focused treatment for Afghan, Great lakes region african, and iraqi Refugee adults**

Inception: Inception came from previous work with other community groups; present study guided by refugees, formers students and community service providers
Design: Community involved in designing the interview protocols, and participant recruitment
Implementation: target population involved in implementation; all interpreters and interviewers were refugees
Analysis: target population involved in analysis; active involvement in asking what evidence is meaningful
Evaluation: CAC were involved in conversation concerning the use of CAC; have voice opinions that a community intervention paradigm should be used which the researchers appear to have heeded.
Dissemination*: target population involved in dissemination; 2nd and 8th author are refugees*

9. Grigg-Saito, Och et al.

Aim & Target population: *Outreach to reduce health disparities in cardiovascular disease and diabetes among Cambodian refugees*

Inception: *Coalition formed prior to this intervention to address needs of Cambodian community - resulted in the funding bid for this work; the coalition involved community leaders and elders*
Design: *Collaboration amongst the coalition to develop a community action plan. Target population took part in focus groups; community forum involving target population (engagement event) took place*
Implementation: *Cambodian community health programme Staff health educators go door-to-door, staff also encouraged participation from elders; Staff conducted business outreach and educational groups/workshops; A peer-support group was established; elders council (advisory group) established at the behest of elder program participants*
Analysis: *No explicit evidence of non-academic stakeholder involvement, engagement, or influence.*

For peer review only

Evaluation: *Data was reported back to staff and steering committee periodically, everyone was able to contribute to lessons learned, draw conclusions and make suggestions for improvements based on the evaluation.*
Dissemination*: No explicit evidence of non-academic stakeholder involvement, engagement, or influence.*

10. Henderson, Slater

Aim & Target population: **Nutrition information for newly arrived immigrants**

Inception: based on previous community-based participatory research
Design: Community consultation
Implementation: *No explicit evidence of non-academic stakeholder involvement, engagement, or influence.*
Analysis: *No explicit evidence of non-academic stakeholder involvement, engagement, or influence.*
Evaluation: community consultation on evaluation
Dissemination*: No explicit evidence of non-academic stakeholder involvement, engagement, or influence.*

11. Jacquez, Vaughn et al. 2019

Aim & Target population: *Implementation of a stress reduction intervention for latino immigrants.*

Inception: *Manifested from a previous relationship with latinos unidos por la salud to promote health and healthcare for the local latino community; community members applied and were selected as co-researchers*
Design*: Community group co-researchers worked with academic partners to identify primary outcomes, and that health-worker delivered strategies were the preferred intervention option.*
Implementation: *Co-researchers recruited and worked with participants to identify strategies with participants.*
Analysis: *co-researchers and academic partners identified the primary outcomes*
Evaluation: *Academic and community partners shared decision-making in all aspects of the research process, including evaluation.*
Dissemination*: Academic and community partners shared decision-making in all aspects of the research process, including dissemination.*

12. Kaiser, Martinez et al. 2015

Aim & Target population: *Adaptation of a culturally relevant nutrition and exercise program for Mexican immigrant parents with young children*

Inception: *Researchers conceived through reviewing literature and involving the target population in focus groups to assess their interpretation and prioritisation of key childhood obesity messages.*
Design: *Researchers performed a literature search and involved the target population in focus groups; the researchers presented the plans for a nutrition program to a local advisory committee whom convened quarterly and gave advice on how to deal with specific issues*
Implementation: *A local educator with experience of family counselling was hired to deliver the intervention*
Analysis: *No explicit evidence of non-academic stakeholder involvement, engagement, or influence.*
Evaluation: *pilot testing with a subsection of families who were invited to give feedback; educator and specialist would evaluate intervention events after each one for changes; participants were invited to focus groups to report back on the intervention.*
Dissemination*: No explicit evidence of non-academic stakeholder involvement, engagement, or influence.*

For peer review only

13. Kandula, Dave et al. 2016

Aim & Target population: *Exercise intervention for south-asian mother with risk factors for diabetes*

Inception: *Local community group and researchers collaboratively chose the focus of the research*
Design: *Community partner and academics adapted and applied evidence-based behaviour change principles in partnership; a memorandum of understanding was agreed by the partners: The community group was involved in design, implementation, recruitment and evaluation.*
Implementation: *Community partner was responsible for recruitment and outreach*
Analysis: *No explicit evidence of non-academic stakeholder involvement, engagement, or influence.*
Evaluation: *The community partner was involved in evaluation; participants were involved in the evaluation of the intervention through qualitative interviews.*
Dissemination: *No explicit evidence of non-academic stakeholder involvement, engagement, or influence.*

14. Karasz, Raghavan et al. 2015

Aim & Target population: *Development of an asset-building mental health intervention for Bangladeshi immigrant women*

Inception: *Outgrowth from previous partnerships; the development involved local physicians, a health advocate and researchers.*
Design: *The target population were included as part of the intervention design group*
Implementation: *No explicit evidence of non-academic stakeholder involvement, engagement, or influence.*
Analysis: *No explicit evidence of non-academic stakeholder involvement, engagement, or influence.*
Evaluation: *No explicit evidence of non-academic stakeholder involvement, engagement, or influence; target population were involved in the evaluation through research methods.*
Dissemination: *No explicit evidence of non-academic stakeholder involvement, engagement, or influence.*

15. Kim, Koniak-Griffin et al. 2004

Aim & Target population: *Cardovascular health promotion among latino immigrants*

Inception: *Collaboration with local department of health and nurses*
Design: *Local department of health refined area for targeting; 2 community based organizations and the formation of an advisory board (including migrants) was instrumental in tailoring the study to the target community (e.g. changing name from lay health advisor to promotoras de salud).*
Implementation: *Lay health advisors were recruited and trained, by the local department of health, to deliver the intervention*
Analysis: *No explicit evidence of non-academic stakeholder involvement, engagement, or influence.*
Evaluation: *Feedback from the local health department, community advisory boards and lay health educators allowed flexible iteration of the intervention*
Dissemination: *No explicit evidence of non-academic stakeholder involvement, engagement, or influence.*

16. Lam, McPhee et al. 2003

For peer review only

Aim & Target population: *Increase pap/cervical cancer screening among Vietnamese-american women*<br><br>***Inception:*** *No explicit evidence of non-academic stakeholder involvement, engagement, or influence.*<br>***Design:*** *Shared with the REACH (community) coalition (who had overall power); community forum was held with 200 vietnamese-american women and their families to discuss cervical cancer and brainstorm strategies to increase screening (the REACH coalition then took findings to generate the intervention); The results were shared with a second community forum, with feedback used to finalise the intervention (by the coalition).*<br>***Implementation:*** *The lay health worker campaign was community-led; Lay health educators were the target population; the community coalition was involved in implementing a media campaign.*<br>***Analysis:*** *No explicit evidence of non-academic stakeholder involvement, engagement, or influence; institutional review board deemed partners could not collect data.*<br>***Evaluation:*** *Media campaign was evaluated at a community forum in which community members opinions were sought through questionnaires and discussions. The REACH coalition retained the power to effect changes; lay health worker opinion was solicited through research methods/questionnaires; No evidence of individual health workers being able to influence intervention in progress.*<br>***Dissemination:*** *No explicit evidence of non-academic stakeholder involvement, engagement, or influence.*
**17. Li, Yeh et al. 2019**<br><br>***Aim & Target population:*** *Colorectal cancer prevention among Chinese-americans*<br><br>***Inception:*** *Researchers and leaders from community groups agreed to promote colorectal cancer prevention in Chinese-americans.*<br>***Design:*** *Designed between the researchers with feedback from the community group; Researcher carried out a literature review with feedback from community partner, research with the actual community in the form of qualitative interviews was conducted to inform the design; Appears community group did have influence, changed from a web-based idea due to community group input.*<br>***Implementation:*** *Delivered by the researchers*<br>***Analysis:*** *No explicit evidence of non-academic stakeholder involvement, engagement, or influence.*<br>***Evaluation:*** *The community group members were involved in filling out surveys as part of the evaluation*<br>***Dissemination:*** *No explicit evidence of non-academic stakeholder involvement, engagement, or influence.*
**18. Nilvarangkul, McCann et al. 2011**<br><br>***Aim & Target population:*** *Enhancing health-related quality of life for Laotian migrants*<br><br>***Inception:*** *Conceived by researchers with a broad action plan developed in collaboration with the target population*<br>***Design:*** *The target population were involved in interviews used to shape the intervention; meetings were held with community workers and stakeholders to create action plans*<br>***Implementation:*** *Community workers, stakeholders and researchers implemented the action plan*<br>***Analysis:*** *No explicit evidence of non-academic stakeholder involvement, engagement, or influence.*<br>***Evaluation:*** *Meetings were held with community workers and stakeholders as part of the formative evaluation; intervention participants were followed up via qualitative data collection*<br>***Dissemination:*** *No explicit evidence of non-academic stakeholder involvement, engagement, or influence.*
**19. Pinsker, Call et al. 2017**

For peer review only

Aim & Target population: *Tobacco use prevention videos for Somali youth*

Inception: *No explicit evidence of non-academic stakeholder involvement, engagement, or influence.*
Design: *There was indirect involvement through focus groups of the target population; there was direct involvement of the target population in developing the videos, building upon the focus groups.*
Implementation: *Target population were involved in shaping the implementation of the intervention*
Analysis: *No explicit evidence of non-academic stakeholder involvement, engagement, or influence.*
Evaluation: *Target population evaluated the intervention through participant interviews*
Dissemination: *No explicit evidence of non-academic stakeholder involvement, engagement, or influence.*

20. Quandt, Grzywacz et al. 2013

Aim & Target population: *Pesticide safety intervention for migrant farmworkers*

Inception: *Based on an existing relationship between the university and community partners*
Design: *Based on an existing partnership; not immediately obvious how taget population and non-academic stakeholders were involved.*
Implementation: *Implemented by the community partners; lay health promoters were recruited from within the community and trained by the community partner*
Analysis: *No explicit evidence of non-academic stakeholder involvement, engagement, or influence.*
Evaluation: *No explicit evidence of non-academic stakeholder involvement, engagement, or influence.*
Dissemination: *No explicit evidence of non-academic stakeholder involvement, engagement, or influence.*

21. Solorio, Norton-Shelpuk et al. 2014

Aim & Target population: *HIV prevention messages for young Latino immigrant MSM*

Inception: *There is a previous community-university relationship, uncertain as to where the inception for the project came.*
Design: *Storyboard/intervention was put before the target group in focus groups, Latino MSM were hired to facilitate the focus groups and help interpret the findings*
Implementation: *Intervention was actioned by an external digital marketing/media team who used the design focus groups to develop the campaign*
Analysis: *Latino MSM were hired to help interpret the findings*
Evaluation: *The target population were involved in focus groups to assess their comprehension of the intervention*
Dissemination: *No explicit evidence of non-academic stakeholder involvement, engagement, or influence.*

22. Song, Han et al. 2010

Aim & Target population: *Translating dietary guidelines into a culturally tailored nutrition education program for Korean-American immigrants with type 2 diabetes*

Inception: *There is a previous relationship between researchers and community, but no clear link as to how this explicitly influenced inception*
Design: *No explicit evidence of non-academic stakeholder involvement, engagement, or influence.*
Implementation: *No explicit evidence of non-academic stakeholder involvement, engagement, or influence; appears the education program was delivered by nurses and dieticians*

For peer review only

Analysis: *No explicit evidence of non-academic stakeholder involvement, engagement, or influence.*
Evaluation: *Target population were involved in evaluating the interventions' acceptability*
Dissemination: *No explicit evidence of non-academic stakeholder involvement, engagement, or influence.*

23. Suarez-Balcazar, Early et al. 2018

Aim & Target population: *Healthy lifestyle promotion among Latino immigrant families with youth with disabilities*

Inception: *No explicit evidence of non-academic stakeholder involvement, engagement, or influence.*
Design: *Focus groups with the target population; interviews and conversations with community stakeholders and agency personnel; research built on by collaboration with mothers and staff from community organisations*
Implementation: *Delivered by bilingual occupational therapy and nutrition students; co-facilitated by mothers who presented Zumba sessions and prepared healthy snacks.*
Analysis: *No explicit evidence of non-academic stakeholder involvement, engagement, or influence.*
Evaluation: *Evaluated through focus groups at the end of the intervention*
Dissemination: *No explicit evidence of non-academic stakeholder involvement, engagement, or influence.*

24. Vaughn & Jacquez. 2019

Aim & Target population: *Stress and coping intervention for Latino immigrants*

Inception: *There are existing relations between the researchers and community; migrant community members were selected to be co-researcher*
Design: *Design input came from researchers and community co-researchers*
Implementation: *Community co-researchers implemented the intervention*
Analysis:
Evaluation: *Co-researchers provided evaluation through interviews with the researchers*
Dissemination: *No clear evidence, though does mention co-researchers want to be involved in dissemination including publications and presentations*

25. Wieland, Nigon. 2019

Aim & Target population: *Tuberculosis screening for migrant adults*

Inception: *Conceived and initiated by a community organisation*
Design: *Academic and non-academic community organisation co-designed the intervention; not sure of the involvement of the target population*
Implementation: *Intervention was implemented by community organisation staff, along with clinical/nursing staff and students*
Analysis: *Evidence was collected and analysed by local health practitioners*
Evaluation: *No explicit evidence of non-academic stakeholder involvement, engagement, or influence.*
Dissemination: *No explicit evidence of non-academic stakeholder involvement, engagement, or influence.*

26. Wieland, Njeru et al. 2017

Aim & Target population: *Digital storytelling intervention for immigrants and refugees with diabetes*

Inception: *Impetus came from an existing researcher-community partnership; diabetes co-agreed as an area for research and intervention*

For peer review only - http://bmjopen.bmj.com/site/about/guidelines.xhtml

Design: survey developed by community-academic partnership to understand target population, attitudes and behaviours in T2DM; focus groups were conducted to further inform intervention content and to identify gifted storytellers; storytellers were recruited by community partners and their stories were captured, recorded and edited to derive the final intervention product.
Implementation: *No explicit evidence of non-academic stakeholder involvement, engagement, or influence.*
Analysis: *No explicit evidence of non-academic stakeholder involvement, engagement, or influence.*
Evaluation: *participants completed follow-up surveys*
Dissemination: *No explicit evidence of non-academic stakeholder involvement, engagement, or influence.*

27. Wieland, Wies. 2012

Aim & Target population: **Physical activity and nutrion program for immigrant and refugee women**

Inception: Conceived from a community-academic partnership with long-standing ties; formalised to use CBPR to improve migrant health
Design: Exercise programme piloted; refugee and immigrant women were included as focus group participants
Implementation: Implemented by researchers and community organisation; community organisation recruited women to the programme; community organisation provided facilities
Analysis: *No explicit evidence of non-academic stakeholder involvement, engagement, or influence.*
Evaluation: *No explicit evidence of non-academic stakeholder involvement, engagement, or influence.*
Dissemination: *No explicit evidence of non-academic stakeholder involvement, engagement, or influence.*

28. Williams, Ochsner et al. 2010

Aim & Target population: *Health and safety training for Latino day laborers*

Inception: *Inception appears to come from advocacy/community groups*
Design: *Focus groups held with the target population to identify typical exposures and situations to be addressed by peer trainers; curriculum was revised with feedback from these peer trainers*
Implementation: *Delivered by peer-trainers*
Analysis: *No explicit evidence of non-academic stakeholder involvement, engagement, or influence.*
Evaluation: *Evaluated through focus group discussions and pre & post-training surveys;*
Dissemination: *No explicit evidence of non-academic stakeholder involvement, engagement, or influence.*

For peer review only

BMJ Open

Participatory approaches in research to develop health interventions for migrants: a systematic review

Journal:	*BMJ Open*
Manuscript ID	bmjopen-2021-053678.R1
Article Type:	Original research
Date Submitted by the Author:	04-Aug-2021
Complete List of Authors:	Rustage, Kieran; St George's University of London, Institute for Infection and Immunity<br>Crawshaw, Alison; St George's University of London, Institute for Infection and Immunity<br>Majeed-Hajaj, Saliha; Queen Mary University of London<br>Deal, Anna; St George's University of London, Institute for Infection and Immunity; London School of Hygiene & Tropical Medicine<br>Nellums, Laura ; University of Nottingham, Division of Epidemiology and Public Health<br>Ciftci, Yusuf; Doctors of the World<br>Fuller, Sebastian; St George's University of London, Institute for Infection and Immunity<br>Goldsmith, Lucy; St George's University of London, Institute for Infection and Immunity, and Population Health Research Institute;<br>Friedland, Jon; St George's University of London<br>Hargreaves, Sally; St George's University of London, Institute for Infection and Immunity
**Primary Subject Heading**:	Global health
Secondary Subject Heading:	Public health, Patient-centred medicine
Keywords:	PUBLIC HEALTH, STATISTICS & RESEARCH METHODS, EDUCATION & TRAINING (see Medical Education & Training)

SCHOLARONE™
Manuscripts

I, the Submitting Author has the right to grant and does grant on behalf of all authors of the Work (as defined in the below author licence), an exclusive licence and/or a non-exclusive licence for contributions from authors who are: i) UK Crown employees; ii) where BMJ has agreed a CC-BY licence shall apply, and/or iii) in accordance with the terms applicable for US Federal Government officers or employees acting as part of their official duties; on a worldwide, perpetual, irrevocable, royalty-free basis to BMJ Publishing Group Ltd ("BMJ") its licensees and where the relevant Journal is co-owned by BMJ to the co-owners of the Journal, to publish the Work in this journal and any other BMJ products and to exploit all rights, as set out in our licence.

The Submitting Author accepts and understands that any supply made under these terms is made by BMJ to the Submitting Author unless you are acting as an employee on behalf of your employer or a postgraduate student of an affiliated institution which is paying any applicable article publishing charge ("APC") for Open Access articles. Where the Submitting Author wishes to make the Work available on an Open Access basis (and intends to pay the relevant APC), the terms of reuse of such Open Access shall be governed by a Creative Commons licence – details of these licences and which Creative Commons licence will apply to this Work are set out in our licence referred to above.

Other than as permitted in any relevant BMJ Author's Self Archiving Policies, I confirm this Work has not been accepted for publication elsewhere, is not being considered for publication elsewhere and does not duplicate material already published. I confirm all authors consent to publication of this Work and authorise the granting of this licence.

For peer review only

Participatory approaches in research to develop health interventions for migrants: a systematic review

Kieran Rustage,[1] Alison Crawshaw,[1] Saliha Majeed-Hajaj,[2] Anna Deal,[1] Laura B Nellums,[3] Yusuf Ciftci,[4] Sebastian Fuller,[1] Lucy Goldsmith,[1] Jon S Friedland,[1] Sally Hargreaves[1]

Corresponding author: Sally Hargreaves (s.hargreaves@sgul.ac.uk)

Word count: 4699

Key Words: Participatory Research; Systematic Review; Migrant Health; Health Intervention

Institutional affiliations:

1. The Migrant Health Research Group, Institute for Infection & Immunity, St George's University of London, United Kingdom
2. Queen Mary University of London, London, United Kingdom
3. University of Nottingham, Nottingham, United Kingdom
4. Doctors of the World UK, part of the Médecins du Monde network

For peer review only - http://bmjopen.bmj.com/site/about/guidelines.xhtml

For peer review only

Abstract

Objective

Analysis of participatory approaches in research to develop health interventions for migrants, and how approaches embody core participatory principles of inclusivity and democracy.

Design

A systematic review of original articles. Electronic searches within the databases MEDLINE, Embase, Global Health and PsychINFO (from inception – Nov 2020).

Eligibility criteria for study selection

Original peer-reviewed articles that reporting research to develop and implement a health intervention for migrants, incorporating using participatory approaches. We defined migrants as foreign-born individuals. Only articles reporting the full research cycle (inception, design, implementation, analysis, evaluation, dissemination) were included.

Data extraction

We extracted information related to who was involved in research (migrants or other non-academic stakeholders), the research stage at which they were involved (inception, design, implementation, analysis, evaluation, dissemination), the method of their involvement, and how this aligned with the core principles of participatory research – categorising studies as exhibiting active or pseudo (including proxy and indirect) participation.

Results

1793 publication were screened of which 28 were included in our analysis. We found substantial variation in the application of participatory research approaches in research designing health interventions targeting migrants: across 168 individual research stages analysed across the 28 studies, we recorded 46 instances of active participation of migrants, 30 instances of proxy

For peer review only - http://bmjopen.bmj.com/site/about/guidelines.xhtml

participation, and 24 instances of indirect participation. all studies involved non-academic stakeholders in at least one stage of the research, only two studies exhibited evidence of active participation of migrants across all research stages. Evidence is limited due to the variability of terms and approaches used.

Conclusions

Important shortfalls in the meaningful inclusion of migrants in developing health interventions exists, suggesting a more rigorous and standardised approach is warranted to better define and deliver participatory research and improve quality.

Registration

This review followed PRISMA guidelines and is registered on the Open Science Framework (osf.io/2bnz5).

For peer review only - http://bmjopen.bmj.com/site/about/guidelines.xhtml

Strengths and Limitations

- This systematic review represents a robust and novel assessment of the application of participatory approaches and principles toward research into health interventions involving migrants.
- This review casts a critical lens over the relationship between how participatory approaches are applied, and how participatory principles such as inclusivity and democracy are embodied.
- Due to the varied and interchangeable use of participatory research terms the categorisations and definitions we use could be interpreted differently by others.
- This review is limited by the lack of clear and consistent reporting of participatory methods used, suggesting that guidelines must be developed and more consistently adopted to improve transparency in all participatory research.
- This review does not address possible associations between participatory methods and final health or research outcomes, which should be better considered in future research.

For peer review only

Introduction

Considerable emphasis is now being placed on ensuring patient and public engagement in health research, including striving for greater involvement of marginalised groups such as migrants and ethnic minorities.[1,2] However, whether this is effectively and meaningfully done in practice to ensure truly patient-centred research has yet to be fully elucidated. Participatory research represents a distinct research paradigm in which research is done collaboratively with the individuals whose lived experiences and actions are the subject of study, as active partners who share power and influence over research processes and outcomes.[3-6]

Two fundamental principles of participatory research that underpin the ability for stakeholders to effectively co-operate and share power are those of inclusivity and democracy, particularly in relation to those directly affected by the research in question.[3] That is to say, has the research included the individuals the research would otherwise be about, and have these individuals, during their inclusion, had influence or power over research decisions on par with the research professionals? Included under the umbrella of participatory research approaches are more specific methodologies which look to uphold these principles, including: community based participatory research (CBPR)[7]; action research[8]; some patient & public involvement (PPI)[9]; as well as broader derivatives such as community-based collaborative action research. Participatory research holds the potential to bridge the gap between public health research and practice, creating a context in which patients and the public have meaningful influence over research decisions, increasing the relevance and impact of research outcomes to their own lives.[10]

Participatory research is likely to be particularly powerful when working with underserved and marginalised groups such as migrants, where traditional research has frequently failed to provide an appreciable health benefit. Whilst a heterogenous group, comprising a multitude of cultures, ethnicities and socio-cultural circumstances, many migrants can find themselves in vulnerable situations, marginalised by health systems,[11,12] and society alike.[13,14] There is a growing consensus

For peer review only - http://bmjopen.bmj.com/site/about/guidelines.xhtml

around the need for academics and health systems to become more responsive to, and inclusive of refugee and migrant health concerns.[15] Indeed, limited community engagement in public health interventions has already been shown to be effective when working with marginalised groups around a range of health outcomes, and can provide benefits to participants themselves, such as in improving health behaviours and participant self-efficacy.[16] However, the ultimate goal is to conduct participatory research with migrants as a matter of routine, so that research is better centred around, and grounded in the needs of migrant communities.

Despite the potential of participatory research, there are varied interpretations as to how to apply such approaches. A review of peer models in participatory research, in which partnerships with 'insiders' are established reveal a norm in which practices and terms are interchangeable, and inconsistently applied.[17] Challenges exist in deciding who should be involved, and whether involvement should extend beyond the target group (in this case, migrants), to other non-academic stakeholder groups such as community organisations and professionals.[3,17] There are also differing interpretations of the degree of participation required of individuals for research to be considered participatory rather than tokenistic, though it is suggested that unless involved individuals are partners or co-researchers throughout the entirety of a project, the work cannot be participatory.[3,18] Overall, it is widely agreed quality participation is characterised by non-academic stakeholders having opportunities to engage with, make decisions about, and perform leadership roles around such research,[5] empowering the public at the highest level and asserting their right to be involved in decision-making and to influence outcomes. Understanding the different approaches to participatory research and whether the core principles of participatory research are upheld is crucially important if good practice is to be identified.

We therefore did a systematic review to analyse participatory approaches in research to develop health interventions for migrants, through use of a framework which relates categories of participation to core principles of participation (inclusivity and democracy), and collate evidence of

For peer review only - http://bmjopen.bmj.com/site/about/guidelines.xhtml

the benefits of utilising a participatory approach to research, and of the challenges of utilising a participatory approach to research.

For peer review only - http://bmjopen.bmj.com/site/about/guidelines.xhtml

Methods

We did a systematic review, following PRISMA guidelines, which is registered on the Open Science Framework (osf.io/2bnz5). The primary aim of this systematic review was to analyse the use of participatory approaches in research developing health interventions targeting migrants as the intended beneficiaries. Specifically, we established a framework of categories of participation, which related the data we extracted to participatory principles of inclusivity and democracy. Our secondary aims were to describe the challenges and benefits of utilising participatory approaches experienced in the research process.

Inclusion and Exclusion criteria

We included peer-reviewed primary-research reporting on health research into interventions aimed at benefitting migrant populations that described using a participatory approach across the whole research process. Research which purported to use a participatory research approach through descriptors in their introduction and methods, or which utilised a recognised participatory approach such as community-based participatory research (CBPR), action research, or community-based collaborative action research, and specifically targeted migrants, was included in the review. We defined migrants as foreign-born individuals and considered a health intervention to be any initiative, tool, or programme that looked to improve health outcomes, including those related to mental health and health literacy.

Studies were excluded if they did not report on all stages of research into the health intervention: Inception, design, implementation, analysis, evaluation, dissemination.

As such, publications presenting interim results of studies which had not completed the full research cycle, as well as studies specifically focusing on only co-designing interventions were excluded. We took this approach so as not to unfairly penalise ongoing research in our analysis, nor co-designed

For peer review only - http://bmjopen.bmj.com/site/about/guidelines.xhtml

research; we consider co-design to be one component of the broader participatory research paradigm and were most interested in how approaches manifest across the entirety of a research cycle. Studies explicitly defining migrant status according to ethnic or ancestral background but not country of birth were excluded, as were papers where primary data were not reported (e.g., comments, editorials, letters, and reviews).

Search strategy

We individually searched the databases MEDLINE (1946 – November 2020), Embase (1974 – November 2020), Global Health (1910 – November 2020) and PsychINFO (1967 – November 2020) within the Ovid platform utilising a Boolean search strategy with keywords and medical subheadings (MeSH) related to two major themes: migrants and participatory research. There were no geographic or language restrictions. An additional text file outlines the full searches carried out (see Additional file 1). The retrieved hits from each database were combined and deduplicated manually within Rayyan. We identified additional studies through hand searching the bibliographies of publications included after full-text screening.

Study selection

Two reviewers duplicated the title and abstract screening and full-text screening (KR & SM-H), which was carried out using the web-based application Rayyan.[19] The reasons for excluding studies during full-text screening were recorded. Any discrepancies in screening decision between the two initial reviewers were mediated by a third reviewer (AC). Where retrieved articles indicated the existence of a separate methodological article, we also screened this in conjunction with the first article on condition that it was a retrospective report of methods used (across all stages of the research) rather than a prospective outline of planned methods.

Data extraction and analysis

For peer review only - http://bmjopen.bmj.com/site/about/guidelines.xhtml

Studies which reported using participatory research approaches, and which reported on all stages of the research were extracted using a piloted form by KR and SH. We extracted summary data on geographical location, the self-described participatory approach, specific target population, and aims of the research. Data relating to the participatory approach of each study was extracted and analysed to achieve our primary aim. We extracted data on the stages of the research in which migrants were involved (Table 1). Where specific research stages did not involve migrants, but did involve other non-academic stakeholders this was recorded, sub-categorising these groups as community groups/third-sector organisations or professional services. We subsequently extracted data on the methods used to involve migrants (or other non-academic stakeholders) at each stage of the research (inception, design, implementation, analysis, evaluation, and dissemination). An additional file provides details of the summary extracted data (see Additional file 2).

For peer review only

For peer review only - http://bmjopen.bmj.com/site/about/guidelines.xhtml

Table 1. Stages of research and evidence sought for each stage during data extraction

	Research Stages					
	Inception	Design	Implementation	Analysis	Evaluation	Dissemination
Evidence Sought	Who was responsible for having the idea for the research, or initiating the study?	Who was involved in the initial planning and design of the intervention/study? Who decided what the final design of the intervention/study would be?	Who was responsible for implementing/piloting the intervention within the remit of the study? Who decided what this implementation should look like?	Who was involved in analysing data relating to primary endpoints/outcomes? Who decided what these endpoints or outcomes should be?	Who was involved in the overall evaluative of the intervention/study? For example, process evaluation, reflective evaluation? Who had a say in determining successes/failures/future considerations?	Who was involved disseminating findings? What form did this take? Who decided this?

For peer review only

We related extracted information on who was involved, when they were involved, and how they were involved in the described research, relating these factors to participatory principles of inclusion and democracy and categorising them within a framework we developed (Table 2). The framework was developed with reference to the literature, particularly that relating to participatory research as a democratic process and being necessary to implement at all stage of the research.[3,5,18,20] . We used data extracted as to who was involved, and when, to guide our assessment of inclusivity. Specifically, we were concerned with whether the evidence displayed relevant inclusivity, that is, the involvement of migrant individuals that are the target, or intended beneficiary of the health intervention. We used data extracted as to the method and means of involvement to guide our assessment of democracy. In this instance we sought evidence of whether methods employed in the research suggested greater levels of democracy, such as through power-sharing and decision-making mechanisms such as equal voting, or committees for those involved. Within the framework we categorised the aggregated data from each study, with specific reference to migrant individuals, as: Active participation; Pseudo Participation (including proxy and indirect participation); or No explicit evidence of participation. The final framework and definitions were agreed by all co-authors (table 2).

To achieve our secondary aim, we specifically scanned the included articles for evidence of any evaluation of the use of participatory approaches within the research. We extracted this data, where found, and categorised it as representing a challenge or benefit associated with the use of participatory approaches toward the overall research process.

Patient and Public Involvement

Members of our authorship team have past and current experience of working within third sector organisations. This experience helped to frame the research questions, and definitions used in the analysis. However, lay-patients and public specifically, were not involved in this research.

For peer review only - http://bmjopen.bmj.com/site/about/guidelines.xhtml

Table 2. Framework of the category of participation, with definitions, criteria, and relationships to participatory principles applied to aggregated data extracted in this review.

Category of Participation		Definition	Criteria	Relationship to participatory principles
Active Participation		Migrants) appeared to be both actively involved, and wielded influence in decisions relating to the research.	• Migrant individuals were involved in this stage of the research.<br>&<br>• Individuals involved appeared to have direct power and influence over the research stage through shared processes with researchers such as voting, or committees.	Relevant inclusivity & greater democracy with regards to migrant's involvement.
Pseudo-participation	Proxy-participation	Community/third-sector organisations and/or professional services are actively involved and wield influence in decisions related to the research stage ahead (or in lieu) of migrants.	• There is uncertain/no clear evidence migrant individuals were involved.<br>Or<br>• Where migrant individuals were involved, they did not appear to have direct power and influence over the research through shared processes with researchers such as voting, or committees. Rather, they were appeared to be involved as research subjects, in surveys or focus groups.<br>But<br>• Third-sector organisations and/or professional services were involved.<br>&<br>• Third-sector organisation and/or professional services appeared to have direct power and influence over the research stage through	There may be relevant inclusivity, but lesser democracy with regards to migrant involvement.<br><br>There may be greater democracy with other stakeholders.

For peer review only

			shared processes with researchers such as voting, or committees.	
	Indirect participation	Migrants' involvement is restricted to activities in which they are research subjects (surveys, focus groups, interviews). No other stakeholders appear to be involved.	• Where migrant individuals were involved, they did not appear to have direct power and influence over the research through shared processes with researchers such as voting, or committees. Rather, they appeared to be involved as research subjects, in surveys or focus groups.<br>&<br>• There is uncertain/no clear evidence as to the involvement of third-sector organisations or professional services.	There may be relevant inclusivity, but lesser democracy with regards to migrant involvement.<br><br>There is no clear evidence of inclusivity or democracy with other stakeholders.
No explicit evidence		No non-academic stakeholders (migrant or otherwise) appear to be involved in this stage of the research.	• There is no clear evidence as to the involvement of migrant individuals.<br><br>• There is no clear evidence as to the involvement of third-sector organisations or professional services.	There is no clear evidence of inclusivity or democracy with migrants or other stakeholders.

For peer review only - http://bmjopen.bmj.com/site/about/guidelines.xhtml

Results

Screening results

Database searches returned 1793 results; a total of 292 duplicates were removed, and 1501 publications were retained for title and abstract screening. Of the 1501 remaining publications, 1357 were excluded during title and abstract screening, and 144 were retained for full-text screening. During full-text screening, 116 publications did not meet our criteria and were excluded, with the reasons for exclusion recorded (Fig 1). Overall, 28 publications met the inclusion criteria and were included in this systematic review (Table 3).

For peer review only

For peer review only - http://bmjopen.bmj.com/site/about/guidelines.xhtml

Table 3. Descriptive characteristic of studies included in this systematic review.

Citation	***Location***	***Self-described participatory approach/methodology***	***Specific Target population***	***Aim of the health intervention***
Afifi, Makhoul. 2011	*Lebanon*	Community-Based Participatory Research (CBPR)	*Palestinian refugee youth*	*Mental health promotion*
Aitaoto, Braun. 2012	*United States*	Community-Based Participatory Research (CBPR)	*Micronesian women*	*Cancer outreach/education*
Baird, Domian. 2015	*United States*	*Community-based collaborative-action research*	*Sudanese Refugee women*	*Addressing health challenges associated with relocation*
Barbee, Kobetz. 2010	*United States*	Community-Based Participatory Research (CBPR)	*Haitian immigrant women*	*To assess the acceptability of HPV self-sampling with community health workers to detect cervical cancer*
Chesla, Chun. 2013	*United States*	Community-Based Participatory Research (CBPR)	*Chinese-American Immigrants*	*To culturally adapt coping skills training for type-2 diabetes*
Evans, suggs. 2019	*United Kingdom*	Community-Based Participatory Research (CBPR)	*African migrants*	*To promote HIV testing uptake*
Forst, Ahonen. 2013	*United States*	Community-Based Participatory Research (CBPR)	*Hispanic construction workers*	*To increase awareness of workplace hazards and self-efficacy; expansion of worker centre agenda to include occupation health and safety*
Goodkind, Amer. 2017	*United States*	Community-Based Participatory Research (CBPR)	*Afghan, Great lakes Region African, and Iraqi refugee adults*	*To address social determinants of health; to improve linkage to mental health services and retention in trauma-focused treatment*
Grigg-Saito, Och. 2008	*United States*	*Community-Based Outreach*	*Cambodian immigrants*	*Strength-based outreach to eliminate cardiovascular disease and diabetes disparities*
Henderson, Slater.	*Canada*	*Action Research*	*Newly arrived migrants*	*To provide tailored nutritional information and support*
Jacquez, Vaughn. 2019	*United States*	Community-Based Participatory Research (CBPR)	*Latino immigrants*	*Stress reduction*
Kaiser, Martinez. 2015	*United States*	Community-Based Participatory Research (CBPR)	*Mexican immigrants*	*To provide obesity prevention education & outreach*
Kandula, Dave. 2016	*United States*	Community-Based Participatory Research (CBPR)	*South Asian immigrant women*	*Exercise intervention for those at risk of diabetes*
Karasz, Raghavan. 2015	*United*	Community-Based Participatory Research	*Bangladeshi immigrant women*	*To provide and intervention tackling common mental disorders*

For peer review only

	States	(CBPR)		
Kim, Koniak-Griffin. 2004	United States	Community-Based Participatory Research (CBPR)	Latino immigrants	To utilise Lay health advisors for cardiovascular health promotion
Lam, Mcphee. 2003	United States	Community-Based Participatory Research (CBPR)	Vietnamese immigrants	To increase pap screening through education and outreach through lay health workers and media
Li, Yeh. 2013	United States	Community-Based Participatory Research (CBPR)	Chinese-American immigrants	To prevent colorectal cancer through education and outreach
Nilvarangkul, McCann. 2011	Thailand	Action Research	Laotian migrants	Enhancement of a quality-of-life model
Pinsker, Call. 2016	United States	Community-Based Participatory Research (CBPR)	Somali youth	To provide a culturally appropriate smoking cessation intervention
Quandt, Grzywacz. 2014	United States	Community-Based Participatory Research (CBPR)	Latino immigrants	To provide Lay health promoter-led pesticide safety education
Solorio, Norton-Shelpuk. 2014	United States	Community-Based Participatory Research (CBPR)	Latino immigrant MSM	To provide HIV prevention outreach for men who have sex with men
Song, Han. 2010	United States	Community-Based Participatory Research (CBPR)	Korean-American immigrants	To translate current dietary guidelines into a culturally tailored nutrition program
Suarez-Balcazar, Early. 2018	United States	Community-Based Participatory Research (CBPR)	Latino immigrant families with youth with disabilities	To provide healthy lifestyle promotion
Vaughn, Jacquez. 2019	United States	Community-Based Participatory Research (CBPR)	Latino immigrants	To reduce stress and increase coping skills
Wieland, Nigon. 2019	United States	Community-Based Participatory Research (CBPR)	Foreign-born	To promote Tuberculosis screening
Wieland, Njeru. 2017	United States	Community-Based Participatory Research (CBPR)	Immigrants and refugees with T2DM	To provide a digital story-telling intervention to improve management of type-2 diabetes among those affected
Wieland, Wies. 2012	United States	Community-Based Participatory Research (CBPR)	Immigrant and refugee women	To provide a physical activity and nutrition program
Williams, Ochsner. 2010	United States	Community-Based Participatory Research (CBPR)	Latino immigrants	Health and safety education in construction

For peer review only - http://bmjopen.bmj.com/site/about/guidelines.xhtml

Study Characteristics

The research articles included in this systematic review were published between 2003 and 2019. Only 13 of the publications had any discernible dates relating to when the reported work was conducted, with these dates being between 2003 and 2018. The majority of the publications related to work carried out in the United States (24 out of 28); the remaining publications related to work carried out in Canada,[21] Lebanon,[22] Thailand,[23] and the United Kingdom.[24] The self-described approach taken by 24 of the 28 included studies was community-based participatory research (CBPR)[22,24-46]; the remaining four studies described using community-based collaborative-action research,[47] community-based outreach,[48] and action research.[21,23] The dominant focus of the included studies was around education or outreach (Table 3), for example around cancer education,[25] or healthy lifestyles promotion;[41] five studies specifically mentioned including refugees (Table 3).[22,29,44,45,47]

Analysis of participatory approaches in research to develop health interventions for migrants

In our analysis, participation varied substantially according to the stage of the research under scrutiny. Only two of the included studies reported explicit evidence of some degree of participation of at least one non-academic stakeholder groups across all research stages (Table 4).[29,30] Overall, we extracted and categorised evidence of the participation of at least one non-academic stakeholder group in 22 studies during the inception;[21-23,25,26,29-34,36,38-40,42-48] 25 studies during the design;[21-23,25-39,41-46,48] 23 studies during implementation;[23-32,34,35,37-43,45-48] 4 studies during analysis;[29,30,39,43] 22 studies during evaluation;[21,23,24,27-37,39-42,44,46-48] and 4 studies during dissemination (Table 4).[26,29,30,42]

For peer review only - http://bmjopen.bmj.com/site/about/guidelines.xhtml

Table 4. Analysis and categorisation of participatory character displayed across research stages within included studies

Citation	*Research Stage*					
	Inception	*Design*	*Implementation*	*Analysis*	*Evaluation*	*Dissemination*
Afifi, Makhoul. 2011	○	◆	X	X	X	X
Aitaoto, Braun. 2012	●	◆	◆	X	X	X
Baird, Domian. 2015	◆	X	○	X	○	X
Barbee, Kobetz. 2010	◆	◆	◆	X	X	◆
Chesla, Chun. 2013	X	◆	●	X	○	X
Evans, suggs. 2019	X	X	○	X	○	X
Forst, Ahonen. 2013	X	●	●	X	◆	X
Goodkind, Amer. 2017	◆	◆	◆	◆	◆	◆
Grigg-Saito, Och. 2008	◆	◆	◆	X	◆	X
Henderson, Slater.	●	○	X	X	○	X
Jacquez, Vaughn. 2019	◆	◆	◆	◆	◆	◆
Kaiser, Martinez. 2015	○	◆	○	X	○	X
Kandula, Dave. 2016	●	●	●	X	●	X
Karasz, Raghavan. 2015	●	◆	X	X	○	X
Kim, Koniak-Griffin. 2004	●	◆	◆	X	◆	X
Lam, Mcphee. 2003	X	◆	◆	X	◆	X
Li, Yeh. 2013	●	●	X	X	○	X
Nilvarangkul, McCann. 2011	◆	◆	●	X	●	X
Pinsker, Call. 2016	X	◆	◆	X	○	X
Quandt, Grzywacz. 2014	●	●	●	X	X	X
Solorio, Norton-Shelpuk. 2014	●	○	●	○	○	X
Song, Han. 2010	●	X	●	X	○	X
Suarez-Balcazar, Early. 2018	X	◆	◆	X	○	X
Vaughn, Jacquez. 2019	◆	◆	◆	X	○	◆

For peer review only - http://bmjopen.bmj.com/site/about/guidelines.xhtml

Wieland, Nigon. 2019	●	●	●	●	X	X
Wieland, Njeru. 2017	●	○	X	X	○	X
Wieland, Wies. 2012	●	○	●	X	X	X
Williams, Ochsner. 2010	●	◆	◆	X	○	X

◆ **Active participation:** Migrants appeared to be both actively involved, and wielded influence in decisions relating to the research.

● **Proxy-participation:** Community/third-sector organisations and/or professional services are actively involved and wield influence in decisions related to the research stage ahead (or in lieu) of migrants.

○ **Indirect participation**: Migrants' involvement is restricted to activities in which they are research subjects (surveys, focus groups, interviews). No other stakeholders appear to be involved.

X **No explicit evidence:** No non-academic stakeholders (migrant or otherwise) appear to be involved in this stage of the research.

For peer review only

However, there was greater variation and divergence in participatory research approaches when considering the degree of participation of migrants. In our analysis only 18 of the 28 included studies exhibit active participation of migrants (as the primary focus and target of the intervention) at any stage of the research process.[22,23,25-31,33-35,37,41,42,46-48] Of these 18 studies, only two display evidence of active participation of migrants at all stages of the research process.[29,30]

Across all 168 individual research stages analysed across the 28 studies, we recorded 46 instances of active participation of migrants; 30 instances of proxy participation; 24 instances of indirect participation; and 68 instances in which there was insufficient evidence to make a determination (Table 4). The active participation recorded also appears to be associated with the stage of the research. There were 7 instances of active participation during study inception;[23,26,30,42,47-49] 16 during design;[22,23,25-27,30,31,33-35,37,41,42,46,48,49] 10 during implementation;[25,26,30,34,35,37,41,42,46,48,49] 2 during analysis;[30,49] 6 during evaluation;[28,30,34,35,48,49] and 4 during dissemination.[26,30,42,49]

Evidence of the benefits of utilising a participatory approach to research

The benefit most often reported among the included articles in utilising participatory approaches was the assertion that interventions were better tailored to the target population through involving non-academic stakeholders.[22,25,27,32,37,41,42,46,50] This included two studies which spoke of the benefits of participatory research in facilitating interventions going beyond more immediately actionable cultural adaptations (such as language-adaptation and ethnically matched providers), to providing interventions that more deeply reflect community values and priorities.[27,42]

Participatory approaches provided benefits through the partnerships established during the research. One study reported how participatory approaches allowed for the modification of the research programme throughout conception, development, and implementation.[34] Multiple publications provided evidence on how iterative feedback from stakeholders during the studies

For peer review only - http://bmjopen.bmj.com/site/about/guidelines.xhtml

could further grow partnerships, improving the recruitment of individuals to implement or take part in the intervention,[21,22,27,32,42,51] and dissemination.[27] One study also highlighted that partnerships were a feasible and appropriate means to support intervention implementation,[24] whilst one set of authors reported that partnerships with non-academics can ultimately strengthen research.[26]

Better relationships between the community and academics were cited as having the capability to enhance the familiarity and trust of individuals involved in participatory research. One study cited that increased trust had direct benefits to research, leading to more open and honest dialogue than in traditional research, improving the accuracy and findings of these activities.[47] Researchers becoming part of ongoing community relations was seen as positive, or a catalyst, acting as an impartial bridge between disparate community groups.[22] Long-lasting partnerships built over the course of participatory research studies was cited as producing a capacity building element, increasing the health-related knowledge and resources of the community which academics partnered with.[28,35,43] Finally, partnerships catalysed a greater degree of understanding of a subject among communities, leading to increased self-determination and the ability to generate change of their own accord.[47]

Evidence of the challenges of utilising a participatory approach to research

Multiple studies highlighted the importance of balancing the culture and expectations of both researchers and migrant individuals to enact participatory research.[22,27,39] For example, one study reported that reaching equitability in the research process and working on level-terms with migrants directly conflicted with the cultural norms of some of these individuals, who may revere authority figures, and so would in normal circumstances defer to their judgement.[47] A further study highlighted challenges exist in bringing together differing stakeholders with varied views and experiences. In these situations it was suggested there is no “one size fits all” approach and that processes must be adapted to individual groups.[35] Noting varied perspective, one study highlights

For peer review only - http://bmjopen.bmj.com/site/about/guidelines.xhtml

the challenge that divergent perspectives as to what is most salient and important to address amongst those involved can present a challenge.[29]

The challenge (and importance) of building rapport and addressing mistrust,[22,23,48] or even research fatigue amongst some groups,[22] was also evident within publications. One set of authors, spoke not just of the need for non-academic stakeholders to trust researchers, but how It is also essential for researchers to reciprocate this trust, and prioritise the collaborative and democratic aim of participatory methods; this was perceived as challenging as it may shift the power dynamic and locus of control in the research away from the academics.[38] Even when partnerships overcome challenges of culture, expectations and trust, there remain other practical challenges to operationalising these partnerships.[33,37]

Challenges in ensuring equitability in research understanding, and balancing the participatory nature of a project, with the standards expected by the wider scientific community were also highlighted.[22,35] Furthermore, one study cited the difficulty of navigating acknowledgement and authorship of non-academics in published materials;[27]a scenario which serves to reiterate power-imbalances that can often persist,[52] in that despite being 'equal partners' in research, migrants may still not be equally recognised. The lack of recognition of the requirements of participatory research in traditional academic circles is also cited as a challenge, with one set of authors stating the need for managerial, institutional, and funder-level buy-in and commitment regarding participatory research.[22] Similarly, institutional review limited participation in at least one study, preventing non-academic stakeholders involvement in data collection and analysis.[35]

Other practical challenges to operationalising participatory research included effective, timely communication,[23,36] and the challenge of working with communities in which the dominant language of the researchers and migrant communities differ.[39,47] Finally, the iterative and tailored nature of

For peer review only - http://bmjopen.bmj.com/site/about/guidelines.xhtml

the interventions produced may also impact on the generalisability of findings,[41] whilst some work could seemingly omit or contradict research evidence due to localising the intervention.[44]

For peer review only - http://bmjopen.bmj.com/site/about/guidelines.xhtml

Discussion

To our knowledge this is the first systematic review to robustly measure the application of participatory approaches and principles to health intervention research with migrants, and specifically examine how core participatory principles of inclusivity and democracy are reflected in this application. Whilst specifically focusing on research with migrants, many of the findings, and the framework discussed are likely to provide insight to all practitioners of participatory research. The 28 studies included reported on a variety of developed health interventions, predominantly revolving around outreach and education. Our analysis shows that 18 of the 28 included studies actively involving migrants themselves, but only two studies actively involved migrants during all stages of the research process. The remaining studies either provide insufficient evidence to determine the participatory approach utilised, or were characterised by pseudo-participation, in which community groups/third-sector organisations were directly involved (proxy-participation), or migrants were only involved through being subjects in research activities (indirect-participation).

The approaches taken to participatory research in the studies included in this systematic review varied. The examples that represent the most participatory approach, according to our analysis, were characterised by consistent relevant inclusivity and greater democracy, which saw co-operation between researchers and migrant individuals (whom had power on par with the researchers) (Table 5). The difference between active participatory approaches, and those we characterised as pseudo-participation, appear subtle when viewed from a research-centric perspective but are stark when considering a participatory perspective. Firstly, indirect participation, in which migrants are involved in activities such as surveys or interviews designed to inform health interventions may represent a perfectly suitable means to guide development and build evidence but do little to distribute power in a participatory manner. The risk that research is framed as participatory whilst failing to develop equitable partnerships has previously been highlighted, and still appears to persist.[3,53] There is also concern that participatory research continues to be one-sided, with a continued focus on and glorification of methods on the part of researchers in studies

For peer review only - http://bmjopen.bmj.com/site/about/guidelines.xhtml

involving migrants, at the expense of participatory principles.[54] Secondly, proxy-participation, which may do more to uphold principles of participatory research, may still be at risk of not equitably involving the actual target population. Uncertainty persists around how to best involve non-academic stakeholders, and ensure those that are involved are representative of the population of interest.[55] Whilst community-groups and/or professional services involvement may at times be the only, or most readily available way to represent the population of interest (due to difficulties (perceived or otherwise) in accessing, or providing access to migrants), they cannot be assumed to be representative of them. Previous research has shown that health-service users can identify different needs to service-providers.[56] Furthermore, whilst overall understandings of involvement processes may align, service-providers may place different values on some aspects of involvement.[57] Therefore, proxy-participation could conceivably skew participatory research away from being centred on migrants' needs.

For peer review only - http://bmjopen.bmj.com/site/about/guidelines.xhtml

Table 5. Descriptive tabulation of two studies classified as displaying active participation throughout all stage of the health intervention research with migrants.

		Research Stage					
		Inception	Design	Implementation	Analysis	Evaluation	Dissemination
Studies	Goodkind, Amer. 2017	Study conceived from previous relations with community groups. The present study was guided by refugees, and community service providers.	The community was involved in designing interview protocols and participant recruitment procedures.	All interpreters and interviewers were refugees; procedures had been agreed during inception and design.	Refugees were involved in analysis and were actively involved in setting the agenda for what evidence was meaningful.	Refugees and community involved in discussions to evaluate the process; indication that the decision a community intervention paradigm be adopted appears to have been adopted and championed by researchers as a result.	Refugees were involved in the dissemination and are co-authors of the research publication.
	Jacquez, Vaughn. 2019	Manifested from a previous relationship with latinos unidos por la salud to promote health and healthcare for the local latino community; co-researchers in this project were drawn from the local community.	co-researchers worked with academic partners to identify primary outcomes, and helped decide that health-worker delivered strategies were the preferred intervention option	Co-researchers recruited and worked with participants to identify strategies for stress reduction.	co-researchers and academic partners identified the primary outcomes.	Academic and community partners shared decision-making in all aspects of the research process, including evaluation.	Academic and community partners shared decision-making in all aspects of the research process, including dissemination.

For peer review only

Upholding the core principles of participatory research, in this instance, democratising research and power-sharing, is particularly pertinent to partnering with migrants. Participatory research origins are firmly rooted in increasing social justice, and the promotion of doing research with, not on or about individuals and communities, particularly those that are disadvantaged.[53] Migrant communities are often marginalised within recipient countries,[13,14] and by local health systems.[11,12] Our categorisations, and the challenges and considerations we highlight speak to the deeper underlying influence of power dynamics, which are present in all research and interactions, and can manifest at individual, interpersonal and structural levels within participatory research.[52] These dynamics should not be overlooked, regardless of the perceived benefits and potential of participatory research approaches, lest participatory research unconsciously becomes a means to reinforce and further entrench power inequities individuals such as migrant participants may experience. Not only is it inappropriate for research to perpetuate or deepen any marginalisation through failing to include migrants' voices, insights, and skills, but there are benefits to the proper utilisation of participatory approaches to the overall research process. Included studies provide evidence of the benefits to participant recruitment, implementation, and dissemination. Researchers also highlighted that the iterative nature of participatory research allows more effective tailoring of work to the needs of migrants, through learning from and embedding migrant partners knowledge and experience into research. Whilst studies we identify predominantly focus on community outreach and education within health research, participatory research could be better utilised across all disciplines. Similar methodology could be employed to better design pharmaceuticals, or on a larger scales, procedures and systems at a governance level.

Effectively partner with migrants requires specific strategies, to address the challenges identified in this review. Some of these strategies include: early participatory involvement to guide research

For peer review only - http://bmjopen.bmj.com/site/about/guidelines.xhtml

priorities, methodological approaches, and strategies to manage ongoing relations; translating and back-translating materials; giving reassurance as to the confidentiality of involvement, and respecting decisions around reporting (particularly as some partners may be undocumented migrants or have precarious legal status); utilising a variety of outreach and recruitment outlets, such as NGOs and religious groups trusted by migrants; and identifying opportunities for bidirectional benefits in the research, and capacity building to facilitate collaborative and democratic participation. Those partnering with migrants must demonstrate flexibility to negotiate potential power divides, and acknowledge and be considerate of residual mistrust that may exist among communities, even after researcher-community relationships appear well established.[58] The challenges and extra consideration highlighted by this review must not be underestimated, whilst from a research perspective, more still needs to be done to assess the impact of participatory approaches on overall research processes and output, as well as assessing whether there are distinct benefits to adopting particular participatory approaches (e.g. active, pseudo). However, if research is to become more democratic, patient-centred, and representative of the populations impacted by its work, traditional scientific approaches are likely to be inadequate, with academic researchers holding the majority of power over research.[59]

Greater adoption of consistent and transparent reporting of participatory research is needed to support the need for more critical analysis of involvement and participatory research.[17,60] Whilst guidelines have been developed,[61] they have not been widely adopted, with no material improvement in the reporting quality of published studies seen within some fields as a result of the their publication, which could be attributed to a lack of awareness of the guidelines existence.[62] Tensions exist as to whether participatory research should be conceptualised and evaluated similarly to traditional research.[60,63] However, we believe reporting can be sympathetic of the need to evidence impact of methods and processes on research. We propose the plain reporting of: who was involved in each element of the research; why these individuals were involved; how they were

For peer review only - http://bmjopen.bmj.com/site/about/guidelines.xhtml

involved; and who ultimately controlled decisions relating the research. These questions should be answered by all research involving non-academic stakeholders, at every stage of the research process. The development of guidelines to support this reporting would simultaneously allow a more complete assessment of how participatory research impacted research outcomes, and greater reflection and evaluation of the participatory approaches employed;[63] Such guidelines and evaluative methods should incorporate and build upon existing monitoring tools, such as those which specifically seek to address existing challenges around power dynamics.[52] Any guidelines must also consider the distinct nature of participatory research, in that conventional evaluation is likely inappropriate, with participatory research, and consequently its' evaluation emphasising internal group evaluation which is done for combined stakeholders in an adaptive and negotiated manner.[64]

Comparing our review to existing literature there appears to be a common trend where academics and research as whole are primarily concerned with the impact or benefits of participatory methods on research processes and outcomes, which is a valid question, but is only encompassed in our research as a secondary aim. Nevertheless, our findings as to challenges and benefits corroborate and align with existing research that participatory approaches can provide benefits particularly to the recruitment and retention of trial participants.[65-68] Challenges associated with participatory research that is previously reported and complementary to our findings often involve methodological challenges around collecting, interpreting, and disseminating research.[65,67] We found, similar to previous reviews, that the extent of engagement and involvement of non-academic stakeholders is highly varied, and that research that is expressly participatory is often limited.[17,65-68] We also find similar trends to these previous review in that there is also variability in the use of naming conventions, and application of reported approaches across all fields and topics of research.[17,66]

Our review has several limitations and caveats. Firstly, we acknowledge the taxonomy of terminology around participatory research is not standardised and terms are used inconsistently. This is an ongoing challenge within the field which has previously been evidenced in similar

For peer review only - http://bmjopen.bmj.com/site/about/guidelines.xhtml

reviews.[17] As such, the categorisations we have introduced and used in this review may be defined differently by others. There is also the possibility that additional publications that don't explicitly use the same language as we have are present in the literature, and that these articles may have been missed by our searches. Furthermore, whether the included studies categorised in our analysis exhibit greater or lesser participation is potentially immaterial to the quality of the research carried out, or the impact of the final intervention; quality criteria for participatory research have not been agreed upon or widely adopted. The amalgamation of these limitations is that formal assessments of study quality, certainty around evidence, and reporting biases, are not readily applied to this systematic review as for more homogenised approaches and methods, such as clinical trials. A further consideration relates to the reporting of benefits and challenges, in that it is often unclear which individuals or groups drove evaluations, and whether academics alone, or academics and communities in partnership decided on what was reported. It is conceivable, given the subjective nature of some of the reported items, that differing sets of benefits and challenges would be reported dependent on the populations involved. This review represents our attempt to cast a critical lens over how the principles of participatory research are applied in practice. Our conceptual focus on migrants' involvement is therefore not intended to denigrate the efforts of third-sector organisations or professionals services, whose involvement we may have classified as proxy-participation. Fundamentally we believe the examined studies are inherently more participatory than traditional research endeavours, for having even considered and attempted to involve non-academic stakeholders. We recognise the challenges associated with participatory research, and as stated, hold no assumptions about the extent of participation and its' association with beneficial outcomes for target populations.

For peer review only

Conclusion

In conclusion, participatory approaches to research for health interventions aimed at migrants are insufficiently applied and reported. We provide evidence that the application of approaches does not fully embody core principles of participatory research, particularly relating to providing decision-making power to individuals ultimately affected by the research. Those who wish to engage in participatory research must consider the approach they take, being cognisant and open to reflecting on question of representation, democracy, and overall power dynamics, and from this critically analysing whether their approach is sufficient to achieve high-quality participation, not just high-quality research. Crucially, guidelines for reporting of participatory research methods must be introduced. This will enable all parties, from academics to communities to better assess the participatory nature of individual research projects and is an important pre-requisite to exploring the overall impact of participatory research, which currently remains inadequately understood.

For peer review only

Declarations

Ethics approval and consent to participate

This research is a systematic review of existing literature. As such, no human participants were involved.

Consent for publication

Not applicable

Availability of data and materials

All data generated or analysed during this study are included in this published article.

Competing interests

The authors declare that they have no competing interests.

Funding Statement.

This work has been funded by the Rosetrees Trust (M775) and the NIHR (NIHR300072). KR is specifically funded by the Rosetrees Trust (M775), SH, AFC, and LG are funded by the NIHR (NIHR Advanced Fellowship NIHR300072), and SH and AFC are additionally funded by the Academy of Medical Sciences (SBF005\1111). AD is funded by the MRC (MR/N013638/1). We acknowledge the support of the European Society of Clinical Microbiology and Infectious Diseases (ESCMID) Study Group for Infections in Travellers and Migrants (ESGITM).

Author Contributions

The study was conceptualised by KR, LN, SH and JSF with input on finalising the study question and protocol from SM-H and AC. Searches were carried out by KR, and screening done by KR, SM-H and AC. Data extraction and analysis was done by KR, AC and SH. Interpretation of the results and drafting of the manuscript was done primarily by KR with input from all authors (AC, SM-H, AD, LN, YC, SF, LG, JSF, SH). All authors commented upon and approved the final manuscript.

Acknowledgements

Not applicable

For peer review only

Authors' information

Not applicable

Additional Files

Additional file 1.docx – Representative search strategy and terms used in the systematic review. This additional file contains a table displaying a representative search strategy used to source articles through database searching.

Additional file 2.docx – Summary extracted data from studies included in this systematic review. This additional file contains a table displaying the summary extracted data obtained and analysed in this systematic review.

Figure Legends:

Figure 1. PRISMA flow-diagram of the study selection process

For peer review only

For peer review only - http://bmjopen.bmj.com/site/about/guidelines.xhtml

References

1 European Centre for Disease Prevention and Control. Guidance on community engagement for public health events caused by communicable disease threats in the EU/EEA. (ECDC; 2020, Stockholm, 2020).

2 Research., N. I. f. H. UK Standards for Public Involvement. (2019).

3 Bergold, J. & Thomes, S. Participatory Research Methods: A Methodological Approach in Motion. *Forum Qualitative Sozialforschung / Forum: Qualitative Social Research* **13**, doi:http://nbn-resolving.de/urn:nbn:de:0114-fqs1201302. (2012).

4 Position Paper 1: What is Participatory Health Research? , (International Collaboration for Participatory Health Research, Berlin, 2013).

5 Springett, J., Katkey, K., Kongats, K., Zulla, R. & Wilkins, E. Conceptualizing Quality in Participatory Health Research: A Phenomenographic Inquiry. *Forum Qualitative Sozialforschung / Forum: Qualitative Social Research* **17**, doi:http://nbn-resolving.de/urn:nbn:de:0114-fqs1602274. (2016).

6 Crawshaw, A. F. *et al.* What must be done to tackle vaccine hesitancy and barriers to COVID-19 vaccination in migrants? *Journal of Travel Medicine*, doi:10.1093/jtm/taab048 (2021).

7 Israel, B. A., Schulz, A. J., Parker, E. A. & Becker, A. B. Community-based participatory research: policy recommendations for promoting a partnership approach in health research. *Education for health (Abingdon, England)* **14**, doi:10.1080/13576280110051055 (2001).

8 Koshy, E., Koshy, V. & Waterman, H. *Action Research in Healthcare*. Vol. 2021 (SAGE Publications Ltd, 2011).

9 Health Research Authority. *What is public involvement in research?*, 2021).

10 Cargo, M. & Mercer, S. The Value and Challenges of Participatory Research: Strengthening its Practice. *Annual Review of Public Health* **29**, 325-350 (2008).

11 Legido-Quigley, H. *et al.* Healthcare is not universal if undocumented migrants are excluded. doi:10.1136/bmj.l4160 (2019).

12 O'Donnel, c. Health Care Access for Migrants in Europe - Oxford Research Encyclopedia of Global Public Health. *Global Public Health*, doi:10.1093/acrefore/9780190632366.013.6 (2018).

13 Thorbjørnsrud, K. & Figenschou, T. U. Do Marginalized Sources Matter? *http://dx.doi.org/10.1080/1461670X.2014.987549*, doi:10.1080/1461670X.2014.987549 (2014).

14 Migrants perspectives on Brexit and UK immigration policies. (Migrants Rights Network, 2017).

15 Health promotion for improved refugee and migrant health: Technical guidance. (World Health Organization, Geneva, 2017).

16 O'Mara-Eves, A. *et al.* The effectiveness of community engagement in public health interventions for disadvantaged groups: a meta-analysis. *BMC Public Health* **15**, 1-23, doi:doi:10.1186/s12889-015-1352-y (2015).

17 Vaughn, L. M. *et al.* Partnering with insiders: A review of peer models across community-engaged research, education and social care. *Health & social care in the community* **26**, doi:10.1111/hsc.12562 (2018).

18 Cook, T. Where Participatory Approaches Meet Pragmatism in Funded (Health) Research: The Challenge of Finding Meaningful Spaces. *Forum Qualitative Sozialforschung / Forum: Qualitative Social Research* **13**, doi:http://dx.doi.org/10.17169/fqs-13.1.1783 (2012).

19 Ouzzani, M., Hammady, H., Fedorowicz, Z. & Elmagarmid, A. Rayyan—a web and mobile app for systematic reviews. *Systematic Reviews* **5**, 1-10, doi:doi:10.1186/s13643-016-0384-4 (2016).

20 *Core Values, Ethics, Spectrum - The 3 Pillars of Public Participation*, <https://www.iap2.org/page/pillars> (2020).

For peer review only - http://bmjopen.bmj.com/site/about/guidelines.xhtml

21 Henderson, A. & Slater, J. Growing Roots: A Newcomer Nutrition Program Designed Using Action Research Methods. *Ecology of Food and Nutrition* **58**, 430-455, doi:10.1080/03670244.2019.1636792. (2019).

22 Afifi, R. A., Makhoul, J., El Hajj, T. & Nakkash, R. T. Developing a logic model for youth mental health: participatory research with a refugee community in Beirut. *Health Policy Plan* **26**, 508-517, doi:10.1093/heapol/czr001 (2011).

23 Nilvarangkul, K., McCann, T. V., Rungreangkulkij, S. & Wongprom, J. Enhancing a health-related quality-of-life model for Laotian migrant workers in Thailand. *Qual Health Res* **21**, 312-323, doi:10.1177/1049732310376635 (2011).

24 Evans, C. *et al.* Mobile phone messaging to promote uptake of HIV testing among migrant African communities in the UK:. *https://doi.org/10.1177/0017896918785928*, doi:10.1177_0017896918785928 (2018).

25 Aitaoto, N., Braun, K. L., Estrella, J., Epeluk, A. & Tsark, J. Design and results of a culturally tailored cancer outreach project by and for Micronesian women. *Prev Chronic Dis* **9**, E82, doi:10.5888/pcd9.100262 (2012).

26 Barbee, L. *et al.* Assessing the acceptability of self-sampling for HPV among Haitian immigrant women: CBPR in action. *Cancer Causes Control* **21**, 421-431, doi:10.1007/s10552-009-9474-0 (2010).

27 Chesla, C. A. *et al.* Testing the efficacy of culturally adapted coping skills training for Chinese American immigrants with type 2 diabetes using community-based participatory research. *Res Nurs Health* **36**, 359-372, doi:10.1002/nur.21543 (2013).

28 Forst, L. *et al.* More than training: Community-based participatory research to reduce injuries among hispanic construction workers. *Am J Ind Med* **56**, 827-837, doi:10.1002/ajim.22187 (2013).

29 Goodkind, J. R. *et al.* Challenges and Innovations in a Community-Based Participatory Randomized Controlled Trial. *Health Education & Behavior* **44**, 123-130, doi:10.1177/1090198116639243 (2017).

30 Jacquez, F., Vaughn, L. M. & Suarez-Cano, G. Implementation of a Stress Intervention with Latino Immigrants in a Non-traditional Migration City. *Journal of Immigrant and Minority Health* **21**, 372-382, doi:doi:10.1007/s10903-018-0732-7 (2018).

31 Kaiser, L. *et al.* Adaptation of a culturally relevant nutrition and physical activity program for low-income, Mexican-origin parents with young children. *Prev Chronic Dis* **12**, E72, doi:10.5888/pcd12.140591 (2015).

32 Kandula, N. R. *et al.* An Exercise Intervention for South Asian Mothers with Risk Factors for Diabetes. *Transl J Am Coll Sports Med* **1**, 52-59 (2016).

33 Karasz, A. *et al.* ASHA: Using Participatory Methods to Develop an Asset-building Mental Health

Intervention for Bangladeshi Immigrant Women. *Progress in Community Health Partnerships: Research, Education, and Action* **9**, 501-512, doi:https://doi.org/10.1353/cpr.2015.0080 (2015).

34 Kim, S., Koniak-Griffin, D., Flaskerud, J. H. & Guarnero, P. A. The impact of lay health advisors on cardiovascular health promotion: using a community-based participatory approach. *J Cardiovasc Nurs* **19**, 192-199, doi:10.1097/00005082-200405000-00008 (2004).

35 Lam, T. K. *et al.* Encouraging Vietnamese-American Women to Obtain Pap Tests Through Lay Health Worker Outreach and Media Education. *J Gen Intern Med* **18**, 516-524, doi:10.1046/j.1525-1497.2003.21043.x (2003).

36 Li, M., Yeh, Y.-L., Sun, H., Chang, B. & Chen, L.-s. Community-Based Participatory Research: a Family Health History-Based Colorectal Cancer Prevention Program Among Chinese Americans. *Journal of Cancer Education : the Official Journal of the American Association for Cancer Education*, doi:10.1007/s13187-019-1486-1 (2019).

For peer review only

For peer review only - http://bmjopen.bmj.com/site/about/guidelines.xhtml

37 Pinsker, E. *et al.* The Development of Culturally Appropriate Tobacco Prevention Videos Targeted

Toward Somali Youth. *Progress in Community Health Partnerships: Research, Education, and Action* **11**, doi:https://doi.org/10.1353/cpr.2017.0017 (2017).

38 Quandt, S. A. *et al.* Evaluating the effectiveness of a lay health promoter-led, community-based participatory pesticide safety intervention with farmworker families. *Health Promot Pract* **14**, 425-432, doi:10.1177/1524839912459652 (2013).

39 Solorio, R., Norton-Shelpuk, P., Forehand, M., Martinez, M. & Aguirre, J. HIV Prevention Messages Targeting Young Latino Immigrant MSM. *AIDS Res Treat* **2014**, 353092, doi:10.1155/2014/353092 (2014).

40 Song, H. J. *et al.* Translating current dietary guidelines into a culturally tailored nutrition education program for Korean American immigrants with type 2 diabetes. *Diabetes Educ* **36**, 752-761, doi:10.1177/0145721710376328 (2010).

41 Suarez-Balcazar, Y. *et al.* Community-based participatory research to promote healthy lifestyles among Latino immigrant families with youth with disabilities. *https://doi.org/10.1080/11038128.2018.1502348*, doi:1502348 (2018).

42 Vaughn, L., Jacquez, F. & Suarez-Cano, G. Developing and Implementing a Stress and Coping Intervention in Partnership With Latino Immigrant Coresearchers. *Translational Issues in Psychological Science* **5**, 62-77, doi:10.1037/tps0000172 (2019).

43 Wieland, M. L. *et al.* Sustainability of a Tuberculosis Screening Program at an Adult Education Center Through Community-Based Participatory Research. *J Public Health Manag Pract* **25**, 602-605, doi:10.1097/phh.0000000000000851 (2019).

44 Wieland, M. L. *et al.* Pilot Feasibility Study of A Digital Storytelling Intervention for Immigrant and Refugee Adults with Diabetes. *Diabetes Educ* **43**, 349-359, doi:10.1177/0145721717713317 (2017).

45 Wieland, M. L. *et al.* Physical activity and nutrition among immigrant and refugee women: a community-based participatory research approach. *Womens Health Issues* **22**, 225-232, doi:10.1016/j.whi.2011.10.002. (2012).

46 Williams, Q., Jr., Ochsner, M., Marshall, E., Kimmel, L. & Martino, C. The impact of a peer-led participatory health and safety training program for Latino day laborers in construction. *J Safety Res* **41**, 253-261, doi:10.1016/j.jsr.2010.02.009 (2010).

47 Baird, M. B. *et al.* Creating a Bridge of Understanding between Two Worlds: Community-Based Collaborative-Action Research with Sudanese Refugee Women. *Public Health Nurs* **32**, 388-396, doi:10.1111/phn.12172 (2015).

48 Grigg-Saito, D., Och, S., Liang, S., Toof, R. & Silka, L. Building on the strengths of a Cambodian refugee community through community-based outreach. *Health Promot Pract* **9**, 415-425, doi:10.1177/1524839906292176 (2008).

49 Goodkind, J. R. *et al.* Challenges and Innovations in a Community-Based Participatory Randomized Controlled Trial. *https://doi.org/10.1177/1090198116639243*, doi:10.1177_1090198116639243 (2016).

50 Coffman, M. J. *et al.* Using CBPR to Decrease Health Disparities in a Suburban Latino Neighborhood. *Hisp Health Care Int* **15**, 121-129, doi:10.1177/1540415317727569 (2017).

51 Documet, P. I. *et al.* A Male Promotores Network for Latinos: Process Evaluation From a Community-Based Participatory Project. *Health Promot Pract* **17**, 332-342, doi:10.1177/1524839915609059 (2016).

52 Roura, M. The Social Ecology of Power in Participatory Health Research. *https://doi.org/10.1177/1049732320979187*, doi:10.1177_1049732320979187 (2020).

53 Macaulay, A. C. Participatory research: What is the history? Has the purpose changed? *Family Practice* **34**, 256-258, doi:https://doi.org/10.1093/fampra/cmw117 (2017).

54 Ozkul, D. Participatory Research: Still a One-Sided Research Agenda? *Migration Letters* **17**, doi:https://journals.tplondon.com/ml/article/view/804 (2020).

For peer review only - http://bmjopen.bmj.com/site/about/guidelines.xhtml

55 Lander, J., Langhof, H. & Dierks, M.-L. Involving patients and the public in medical and health care research studies: An exploratory survey on participant recruiting and representativeness from the perspective of study authors. *PLoS One* **14**, doi:10.1371/journal.pone.0204187 (2019).

56 Edwards, L. *et al.* What matters most to patients about primary healthcare: mixed-methods patient priority setting exercises within the PREFeR (PRioritiEs For Research) project. doi:10.1136/bmjopen-2018-025954 (2019).

57 Rise, M. B. *et al.* Same description, different values. How service users and providers define patient and public involvement in health care. *Health Expect.* **16**, 266-276, doi:10.1111/j.1369-7625.2011.00713.x (2013).

58 Ahmed, A., Vandrevala, T., Hendy, J., Kelly, C. & Ala, A. An examination of how to engage migrants in the research process: building trust through an 'insider' perspective. *https://doi.org/10.1080/13557858.2019.1685651*, doi:CETH-2019-0030.R2 (2019).

59 Green, G. Power to the people: To what extent has public involvement in applied health research achieved this? *Research Involvement and Engagement* **2**, 1-13, doi:doi:10.1186/s40900-016-0042-y (2016).

60 Russel, J., Fudge, N. & Greenhalgh, T. The impact of public involvement in health research: what are we measuring? Why are we measuring it? Should we stop measuring it? *Research Involvement and Engagement* **6**, doi:https://doi.org/10.1186/s40900-020-00239-w (2020).

61 Smith, L., Rosenzweig, L. & Schmidt, M. Best Practices in the Reporting of Participatory Action Research: Embracing Both the Forest and the Trees 1Ψ7:. *http://dx.doi.org/10.1177/0011000010376416*, doi:10.1177_0011000010376416 (2010).

62 Kato, D. *et al.* Reporting Guidelines for Community-Based Participatory Research Did Not Improve the Reporting Quality of Published Studies: A Systematic Review of Studies on Smoking Cessation. *Int J Environ Res Public Health* **17**, doi:10.3390/ijerph17113898 (2020).

63 Staley, K. & Barron, D. Learning as an outcome of involvement in research: what are the implications for practice, reporting and evaluation? *Research Involvement and Engagement* **5**, doi:https://doi.org/10.1186/s40900-019-0147-1 (2019).

64 Campilan, D. in *Forum on Evaluation of International Cooperation Projects: Centering on Development of Human Resources in the Field of Agriculture.*

65 De las Nueces, D., Hacker, K., DiGirolamo, A. & Hicks, L. S. A systematic review of community-based participatory research to enhance clinical trials in racial and ethnic minority groups. *Health services research* **47**, doi:10.1111/j.1475-6773.2012.01386.x (2012).

66 Vaughn, L. M., Jacquez, F., Lindquist-Grantz, R., Parsons, A. & Melink, K. Immigrants as Research Partners: A Review of Immigrants in Community-Based Participatory Research (CBPR). *Journal of Immigrant and Minority Health* **19**, 1457-1468, doi:doi:10.1007/s10903-016-0474-3 (2016).

67 Dadich, A., Moore, L. & Eapen. What does it mean to conduct participatory research with Indigenous peoples? A lexical review. *BMC public health* **19**, doi:10.1186/s12889-019-7494-6 (2019).

68 Wieland, M. L., Njeru, J. W., Alahdab, F., Doubeni, C. A. & Sia, I. G. Community-Engaged Approaches for Minority Recruitment Into Clinical Research: A Scoping Review of the Literature. *Mayo Clinical Proceedings* **93**, 733-743, doi:https://doi.org/10.1016/j.mayocp.2020.03.028 (2021).

For peer review only - http://bmjopen.bmj.com/site/about/guidelines.xhtml

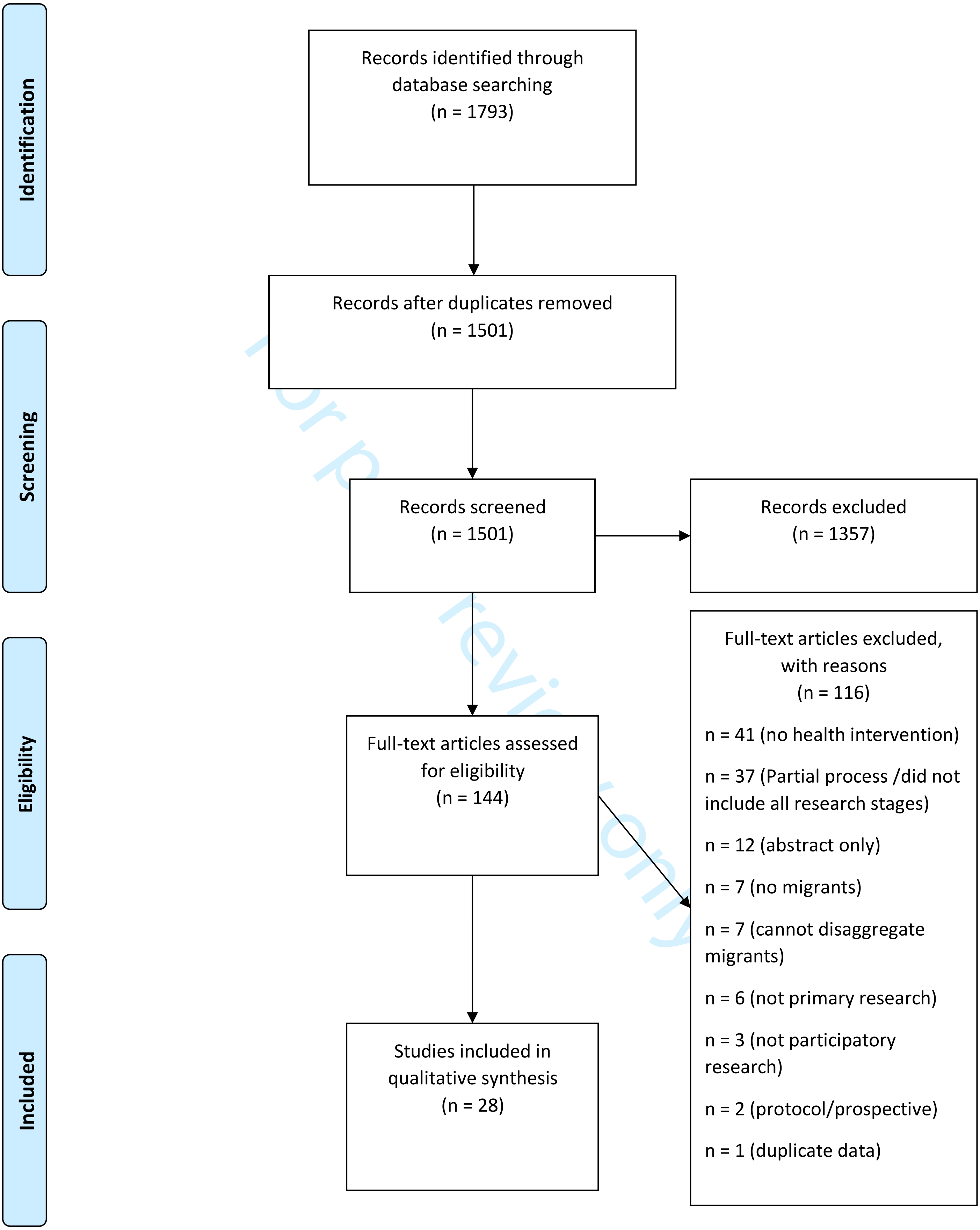

Figure 1. PRISMA flow-diagram of the study selection process

For peer review only - http://bmjopen.bmj.com/site/about/guidelines.xhtml

Additional File 1. Full reproduction of searches used within the individual databases

MEDLINE Database Search Strategy (1946 – November 2020):

1. "Transients and Migrants"/ or Migrant*.mp.
2. Migrat*.mp. [mp=title, abstract, heading word, drug trade name, original title, device manufacturer, drug manufacturer, device trade name, keyword, floating subheading word, candidate term word]
3. Refugee*.mp. or Refugees/
4. Asylum-seeker*.mp.
5. Foreign*.mp.
6. Foreign-born.mp.
7. Non-native*.mp.
8. "Emigrants and Immigrants"/ or Immigra*.mp.
9. Emigra*.mp. or "Emigration and Immigration"/
10. Oversea*.mp.
11. Foreign student*.mp.
12. International Student*.mp.
13. 1 or 2 or 3 or 4 or 5 or 6 or 7 or 8 or 9 or 10 or 11 or 12
14. Co-design*.mp.
15. Co-prod*.mp. [mp=title, abstract, heading word, drug trade name, original title, device manufacturer, drug manufacturer, device trade name, keyword, floating subheading word, candidate term word]
16. Co-creat*.mp. [mp=title, abstract, heading word, drug trade name, original title, device manufacturer, drug manufacturer, device trade name, keyword, floating subheading word, candidate term word]
17. Collab* design*.mp. [mp=title, abstract, heading word, drug trade name, original title, device manufacturer, drug manufacturer, device trade name, keyword, floating subheading word, candidate term word]
18. Collab* prod*.mp. [mp=title, abstract, heading word, drug trade name, original title, device manufacturer, drug manufacturer, device trade name, keyword, floating subheading word, candidate term word]
19. Collab* creat*.mp. [mp=title, abstract, heading word, drug trade name, original title, device manufacturer, drug manufacturer, device trade name, keyword, floating subheading word, candidate term word]
20. Community design*.mp.
21. Community prod*.mp.
22. Community creat*.mp.
23. Community-based participatory research.mp. [mp=title, abstract, heading word, drug trade name, original title, device manufacturer, drug manufacturer, device trade name, keyword, floating subheading word, candidate term word]
24. Participatory design*.mp.
25. Participatory action research.mp. or Community-Based Participatory Research/
26. (Participatory adj4 Research).mp. [mp=title, abstract, heading word, drug trade name, original title, device manufacturer, drug manufacturer, device trade name, keyword, floating subheading word, candidate term word]
27. Action Research.mp.
28. 14 or 15 or 16 or 17 or 18 or 19 or 20 or 21 or 22 or 23 or 24 or 25 or 26 or 27
29. 13 and 28

Embase Database Search Strategy (1974 – November 2020):

1. migrant/ or migrant worker/ or Migrant*.mp.
2. migration/ or Migrat*.mp.
3. refugee/ or Refugee*.mp.
4. asylum seeker/ or Asylum seeker*.mp.
5. Foreign*.mp.
6. Foreign Born.mp.
7. Non-native*.mp.
8. immigration/ or immigrant/ or Immigra*.mp.
9. emigrant/ or emigration/ or Emigrants/ or Emigra*.mp.
10. Oversea*.mp.

11. Foreign Student*.mp. or foreign student/
12. International Student*.mp.
13. 1 or 2 or 3 or 4 or 5 or 6 or 7 or 8 or 9 or 10 or 11 or 12
14. Co-design*.mp.
15. Co-prod*.mp.
16. Co-creat*.mp.
17. Collab* design*.mp.
18. Collab* prod*.mp.
19. Collab* creat*.mp.
20. Community design*.mp.
21. Community prod*.mp.
22. Community creat*.mp.
23. Community-based participatory research.mp. [mp=title, abstract, heading word, drug trade name, original title, device manufacturer, drug manufacturer, device trade name, keyword, floating subheading word, candidate term word]
24. Action research.mp. or action research/
25. Participatory research.mp. or participatory research/
26. Participatory action research.mp.
27. (Participatory adj4 Research).mp. [mp=title, abstract, heading word, drug trade name, original title, device manufacturer, drug manufacturer, device trade name, keyword, floating subheading word, candidate term word]
28. 14 or 15 or 16 or 17 or 18 or 19 or 20 or 21 or 22 or 23 or 24 or 25 or 26 or 27
29. 13 and 28

Global Health Database Search Strategy (1920 – November 2020):

1. Migrant*.mp.
2. Migrat*.mp. [mp=title, abstract, heading word, drug trade name, original title, device manufacturer, drug manufacturer, device trade name, keyword, floating subheading word, candidate term word]
3. Refugee*.mp. or refugees/
4. Asylum-seeker*.mp.
5. Foreign*.mp.
6. Foreign-born.mp.
7. Non-native*.mp.
8. Immigra*.mp.
9. Emigra*.mp. [mp=title, abstract, heading word, drug trade name, original title, device manufacturer, drug manufacturer, device trade name, keyword, floating subheading word, candidate term word]
10. Oversea*.mp.
11. Foreign Student*.mp. or foreign students.sh.
12. International Student*.mp.
13. 1 or 2 or 3 or 4 or 5 or 6 or 7 or 8 or 9 or 10 or 11 or 12
14. Co-design*.mp.
15. Co-prod*.mp. [mp=title, abstract, heading word, drug trade name, original title, device manufacturer, drug manufacturer, device trade name, keyword, floating subheading word, candidate term word]
16. Co-creat*.mp. [mp=title, abstract, heading word, drug trade name, original title, device manufacturer, drug manufacturer, device trade name, keyword, floating subheading word, candidate term word]
17. Collab* design*.mp. [mp=title, abstract, heading word, drug trade name, original title, device manufacturer, drug manufacturer, device trade name, keyword, floating subheading word, candidate term word]
18. Collab* prod*.mp. [mp=title, abstract, heading word, drug trade name, original title, device manufacturer, drug manufacturer, device trade name, keyword, floating subheading word, candidate term word]
19. Collab* creat*.mp. [mp=title, abstract, heading word, drug trade name, original title, device manufacturer, drug manufacturer, device trade name, keyword, floating subheading word, candidate term word]
20. Community design*.mp.
21. Community prod*.mp. [mp=title, abstract, heading word, drug trade name, original title, device manufacturer, drug manufacturer, device trade name, keyword, floating subheading word, candidate term word]
22. Community creat*.mp.

23. Community-based participatory research.mp. [mp=title, abstract, heading word, drug trade name, original title, device manufacturer, drug manufacturer, device trade name, keyword, floating subheading word, candidate term word]
24. Participatory design*.mp.
25. Participatory action research.mp.
26. (Participatory adj4 Research).mp. [mp=title, abstract, heading word, drug trade name, original title, device manufacturer, drug manufacturer, device trade name, keyword, floating subheading word, candidate term word]
27. Action research.mp.
28. 14 or 15 or 16 or 17 or 18 or 19 or 20 or 21 or 22 or 23 or 24 or 25 or 26 or 27
29. 13 and 28

PsychINFO Database Search Strategy (1967 – November 2020):

1. Migrant*.mp.
2. Migrat*.mp.
3. exp REFUGEES/ or Refugee*.mp.
4. exp Asylum Seeking/ or Asylum-seeker*.mp.
5. Foreign*.mp.
6. Foreign-born.mp.
7. Non-native*.mp.
8. exp Immigration/ or Immigra*.mp.
9. Emigra*.mp. [mp=title, abstract, heading word, drug trade name, original title, device manufacturer, drug manufacturer, device trade name, keyword, floating subheading word, candidate term word]
10. Oversea*.mp. [mp=title, abstract, heading word, drug trade name, original title, device manufacturer, drug manufacturer, device trade name, keyword, floating subheading word, candidate term word]
11. Foreign Student*.mp.
12. exp International Students/ or International Student*.mp.
13. 1 or 2 or 3 or 4 or 5 or 6 or 7 or 8 or 9 or 10 or 11 or 12
14. Co-design*.mp.
15. Co-prod*.mp. [mp=title, abstract, heading word, drug trade name, original title, device manufacturer, drug manufacturer, device trade name, keyword, floating subheading word, candidate term word]
16. Co-creat*.mp. [mp=title, abstract, heading word, drug trade name, original title, device manufacturer, drug manufacturer, device trade name, keyword, floating subheading word, candidate term word]
17. Collab* design*.mp.
18. Collab* prod*.mp. [mp=title, abstract, heading word, drug trade name, original title, device manufacturer, drug manufacturer, device trade name, keyword, floating subheading word, candidate term word]
19. Collab* creat*.mp. [mp=title, abstract, heading word, drug trade name, original title, device manufacturer, drug manufacturer, device trade name, keyword, floating subheading word, candidate term word]
20. Community design*.mp.
21. Community prod*.mp.
22. Community creat*.mp. [mp=title, abstract, heading word, drug trade name, original title, device manufacturer, drug manufacturer, device trade name, keyword, floating subheading word, candidate term word]
23. Community-based participatory research.mp. [mp=title, abstract, heading word, drug trade name, original title, device manufacturer, drug manufacturer, device trade name, keyword, floating subheading word, candidate term word]
24. Participatory design*.mp.
25. Participatory research.mp.
26. (Participatory adj4 Research).mp. [mp=title, abstract, heading word, drug trade name, original title, device manufacturer, drug manufacturer, device trade name, keyword, floating subheading word, candidate term word]
27. Participatory action research.mp.
28. Action research.mp. or exp Action Research/
29. 14 or 15 or 16 or 17 or 18 or 19 or 20 or 21 or 22 or 23 or 24 or 25 or 26 or 27 or 28
30. 13 and 29

Additional File 2. Summary of extracted data relating to involvement and engagement of non-academic stakeholders, and their influence over given research stages.

1. Afifi, Makhoul et al. 2011

Aim & Target population: *Development of a mental health promotion intervention for Palestinian refugee youths.*

Inception: *Based on a previous survey of the target population which had been designed with community input*
Design: *Interview and survey of target population; formation of a community youth council with youth council representatives which agreed internal rules and implemented democratic voting re: design decisions*
Implementation: *No explicit evidence of non-academic stakeholder involvement, engagement, or influence.*
Analysis: *No explicit evidence of non-academic stakeholder involvement, engagement, or influence.*
Evaluation: *No explicit evidence of non-academic stakeholder involvement, engagement, or influence.*
Dissemination: *No explicit evidence of non-academic stakeholder involvement, engagement, or influence.*

2. Aitaoto, Braun et al. 2012

Aim & Target population: *Implementation of a cancer outreach programme for Micronesian women*

Inception: *Community group representing the target population approached researchers to highlight this topic*
Design: *Focus groups with the target population; champions from the target population were involved in literature review and aided in designing the outreach programme*
Implementation: *Target population delivered the programme as lay-educators, a decision clearly defined in the design stage by working with the champions*
Analysis: *No explicit evidence of non-academic stakeholder involvement, engagement, or influence.*
Evaluation: *No explicit evidence of non-academic stakeholder involvement, engagement, or influence; reported that the research did not ask the target population about perceptions of the intervention*
Dissemination: *No explicit evidence of non-academic stakeholder involvement, engagement, or influence.*

3. Baird, Domian et al. 2015

Aim & Target population: *To address the health challenges associated with relocation among Sudanese refugees*

Inception: *Collaborative inception between Sudanese community and researchers manifesting from previous research shared to community members, who then formed a committee which worked with the researchers; Whilst topics for intervention were collaboratively developed between researchers and the target population*
Design: *research team developed educational seminars on intervention topics.*
Implementation: *Educational seminars were delivered by the research team; target population were involved in advertising and recruiting other participants.*
Analysis: *No explicit evidence of non-academic stakeholder involvement, engagement, or influence.*
Evaluation: *Focus group discussion to evaluate the process, discuss overall findings, and plan future relationships.*

For peer review only

	Dissemination: *No explicit evidence of non-academic stakeholder involvement, engagement, or influence.*
**4.**	**Barbee, Kobetz et al. 2010**<br><br>***Aim & Target population:*** *To assess the acceptability of HPV self-sampling with community health workers, to detect cervical cancer among Haitian immigrant women.*<br><br>***Inception:*** *Conceived by a campus-community partnership; the partnership is formed of community leaders and academics; community leaders are largely responsible for identifying research needs*<br>***Design:*** *Community partners responsible for defining research focus, recruitment strategies, and data collection methods; academic partners write grants applications and obtain institutional approvals*<br>***Implementation:*** *Community and academic partners contribute collectively to study implementation*<br>***Analysis:*** *No explicit evidence of non-academic stakeholder involvement, engagement, or influence.*<br>***Evaluation:*** *No explicit evidence of non-academic stakeholder involvement, engagement, or influence.*<br>***Dissemination:*** *Community and academic partners contribute collectively to study dissemination*
**5.**	**Chesla, Chun. 2013**<br><br>***Aim & Target population:*** *Cultural adaptation of coping skills training for Chinese-American immigrants with type 2 diabetes.*<br><br>***Inception:*** *No explicit evidence of non-academic stakeholder involvement, or influence; there is mention of a CBPR work group, and a community advisory board, but no indication these groups were involved in the inception.*<br>***Design:*** *A CBPR work group composed of 10 paid staff from community agencies and academic institutions. The work group culturally adapted protocols based on clinical guidelines, clinical knowledge, and previous studies.; A community advisory board met semi-annually and provided oversight on all aspect of the CBPR activities.*<br>***Implementation:*** *Work group members who were bilingual with degree level qualification in social work implemented the intervention.*<br>***Analysis:*** *No explicit evidence of non-academic stakeholder involvement, engagement, or influence.*<br>***Evaluation:*** *Evaluated through research/survey with participants.*<br>***Dissemination:*** *No explicit evidence of non-academic stakeholder involvement, engagement, or influence.*
**6.**	**Evans, Suggs et al. 2019**<br><br>***Aim & Target population:*** *Promoting HIV testing uptake amongst African migrant communities*<br><br>***Inception:*** *No explicit evidence of non-academic stakeholder involvement, engagement, or influence.*<br>***Design:*** *The design was guided by previous formative research conducted by the researchers*<br>***Implementation:*** *Participants were invited by community researchers that represented diverse African communities and genders.*<br>***Analysis:*** *No explicit evidence of non-academic stakeholder involvement, engagement, or influence.*<br>***Evaluation:*** *The target population provided feedback through surveys*<br>***Dissemination:*** *No explicit evidence of non-academic stakeholder involvement, engagement, or influence.*

For peer review only

7. Forst, Ahonen et al. 2013

Aim & Target population: Increase awareness of workplace hazards and self-efficacy among foreign-born Hispanic construction workers; and expand worker centre agenda to include occupational health & safety.

Inception: *No explicit evidence of non-academic stakeholder involvement, engagement, or influence.*
Design: *Worker centres (WC) and academics partnered to set joint goals and specific responsibilities: WC would recruit peer-educators and workers to the training, partner with implementation and evaluation; university partners assumed responsibility for subject protection, evaluation, design and implementation.*
Implementation: *Worker centre staff were responsible for recruiting peer-educators to deliver the curriculum; university partners assumed responsibility for implementation*
Analysis: *No explicit evidence of non-academic stakeholder involvement, engagement, or influence.*
Evaluation: *Iterative evaluation of the intervention with peer-educators in a democratic manner; peer-educators were able to influence study design*
Dissemination: *No explicit evidence of non-academic stakeholder involvement, engagement, or influence.*

8. Goodkind, Amer et al. 2017

Aim & Target population: **to address social determinants of health, improve links to mental health services, and improve retention in trauma-focused treatment for Afghan, Great lakes region african, and iraqi Refugee adults**

Inception: *Inception came from previous work with other community groups; present study guided by refugees, formers students and community service providers*
Design: *Community involved in designing the interview protocols, and participant recruitment*
Implementation: target population involved in implementation; all interpreters and interviewers were refugees
Analysis: *target population involved in analysis; active involvement in asking what evidence is meaningful*
Evaluation: *CAC were involved in conversation concerning the use of CAC; have voice opinions that a community intervention paradigm should be used which the researchers appear to have heeded.*
Dissemination*: target population involved in dissemination; 2nd and 8th author are refugees*

9. Grigg-Saito, Och et al.

Aim & Target population: *Outreach to reduce health disparities in cardiovascular disease and diabetes among Cambodian refugees*

Inception: *Coalition formed prior to this intervention to address needs of Cambodian community - resulted in the funding bid for this work; the coalition involved community leaders and elders*
Design: *Collaboration amongst the coalition to develop a community action plan. Target population took part in focus groups; community forum involving target population (engagement event) took place*
Implementation: *Cambodian community health programme Staff health educators go door-to-door, staff also encouraged participation from elders; Staff conducted business outreach and educational groups/workshops; A peer-support group was established; elders council (advisory group) established at the behest of elder program participants*
Analysis: *No explicit evidence of non-academic stakeholder involvement, engagement, or influence.*
Evaluation: *Data was reported back to staff and steering committee periodically, everyone was able to contribute to lessons learned, draw conclusions and make suggestions for improvements based on the evaluation.*

For peer review only - http://bmjopen.bmj.com/site/about/guidelines.xhtml

***Dissemination**: No explicit evidence of non-academic stakeholder involvement, engagement, or influence.*

10. Henderson, Slater

Aim & Target population: Nutrition information for newly arrived immigrants

***Inception:** based on previous community-based participatory research*
***Design:** Community consultation*
***Implementation:** No explicit evidence of non-academic stakeholder involvement, engagement, or influence.*
***Analysis:** No explicit evidence of non-academic stakeholder involvement, engagement, or influence.*
***Evaluation:** community consultation on evaluation*
***Dissemination**: No explicit evidence of non-academic stakeholder involvement, engagement, or influence.*

11. Jacquez, Vaughn et al. 2019

***Aim & Target population:** Implementation of a stress reduction intervention for latino immigrants.*

***Inception:** Manifested from a previous relationship with latinos unidos por la salud to promote health and healthcare for the local latino community; community members applied and were selected as co-researchers*
***Design**: Community group co-researchers worked with academic partners to identify primary outcomes, and that health-worker delivered strategies were the preferred intervention option.*
***Implementation:** Co-researchers recruited and worked with participants to identify strategies with participants.*
***Analysis:** co-researchers and academic partners identified the primary outcomes*
***Evaluation:** Academic and community partners shared decision-making in all aspects of the research process, including evaluation.*
***Dissemination**: Academic and community partners shared decision-making in all aspects of the research process, including dissemination.*

12. Kaiser, Martinez et al. 2015

***Aim & Target population:** Adaptation of a culturally relevant nutrition and exercise program for Mexican immigrant parents with young children*

***Inception:** Researchers conceived through reviewing literature and involving the target population in focus groups to assess their interpretation and prioritisation of key childhood obesity messages.*
***Design:** Researchers performed a literature search and involved the target population in focus groups; the researchers presented the plans for a nutrition program to a local advisory committee whom convened quarterly and gave advice on how to deal with specific issues*
***Implementation:** A local educator with experience of family counselling was hired to deliver the intervention*
***Analysis:** No explicit evidence of non-academic stakeholder involvement, engagement, or influence.*
***Evaluation:** pilot testing with a subsection of families who were invited to give feedback; educator and specialist would evaluate intervention events after each one for changes; participants were invited to focus groups to report back on the intervention.*
***Dissemination**: No explicit evidence of non-academic stakeholder involvement, engagement, or influence.*

13. Kandula, Dave et al. 2016

Aim & Target population: *Exercise intervention for south-asian mother with risk factors for diabetes*

Inception: *Local community group and researchers collaboratively chose the focus of the research*
Design: *Community partner and academics adapted and applied evidence-based behaviour change principles in partnership; a memorandum of understanding was agreed by the partners: The community group was involved in design, implementation, recruitment and evaluation.*
Implementation: *Community partner was responsible for recruitment and outreach*
Analysis: *No explicit evidence of non-academic stakeholder involvement, engagement, or influence.*
Evaluation: *The community partner was involved in evaluation; participants were involved in the evaluation of the intervention through qualitative interviews.*
Dissemination*: No explicit evidence of non-academic stakeholder involvement, engagement, or influence.*

14. Karasz, Raghavan et al. 2015

Aim & Target population: *Development of an asset-building mental health intervention for Bangladeshi immigrant women*

Inception: *Outgrowth from previous partnerships; the development involved local physicians, a health advocate and researchers.*
Design: *The target population were included as part of the intervention design group*
Implementation: *No explicit evidence of non-academic stakeholder involvement, engagement, or influence.*
Analysis: *No explicit evidence of non-academic stakeholder involvement, engagement, or influence.*
Evaluation: *No explicit evidence of non-academic stakeholder involvement, engagement, or influence; target population were involved in the evaluation through research methods.*
Dissemination: *No explicit evidence of non-academic stakeholder involvement, engagement, or influence.*

15. Kim, Koniak-Griffin et al. 2004

Aim & Target population: *Cardovascular health promotion among latino immigrants*

Inception: *Collaboration with local department of health and nurses*
Design: *Local department of health refined area for targeting; 2 community based organizations and the formation of an advisory board (including migrants) was instrumental in tailoring the study to the target community (e.g. changing name from lay health advisor to promotoras de salud).*
Implementation: *Lay health advisors were recruited and trained, by the local department of health, to deliver the intervention*
Analysis: *No explicit evidence of non-academic stakeholder involvement, engagement, or influence.*
Evaluation: *Feedback from the local health department, community advisory boards and lay health educators allowed flexible iteration of the intervention*
Dissemination: *No explicit evidence of non-academic stakeholder involvement, engagement, or influence.*

16. Lam, McPhee et al. 2003

Aim & Target population: *Increase pap/cervical cancer screening among Vietnamese-american women*

For peer review only

Inception: *No explicit evidence of non-academic stakeholder involvement, engagement, or influence.*
Design: *Shared with the REACH (community) coalition (who had overall power); community forum was held with 200 vietnamese-american women and their families to discuss cervical cancer and brainstorm strategies to increase screening (the REACH coalition then took findings to generate the intervention); The results were shared with a second community forum, with feedback used to finalise the intervention (by the coalition).*
Implementation: *The lay health worker campaign was community-led; Lay health educators were the target population; the community coalition was involved in implementing a media campaign.*
Analysis: *No explicit evidence of non-academic stakeholder involvement, engagement, or influence; institutional review board deemed partners could not collect data.*
Evaluation: *Media campaign was evaluated at a community forum in which community members opinions were sought through questionnaires and discussions. The REACH coalition retained the power to effect changes; lay health worker opinion was solicited through research methods/questionnaires; No evidence of individual health workers being able to influence intervention in progress.*
Dissemination: *No explicit evidence of non-academic stakeholder involvement, engagement, or influence.*

17. Li, Yeh et al. 2019

Aim & Target population: *Colorectal cancer prevention among Chinese-americans*

Inception: *Researchers and leaders from community groups agreed to promote colorectal cancer prevention in Chinese-americans.*
Design: *Designed between the researchers with feedback from the community group; Researcher carried out a literature review with feedback from community partner, research with the actual community in the form of qualitative interviews was conducted to inform the design; Appears community group did have influence, changed from a web-based idea due to community group input.*
Implementation: *Delivered by the researchers*
Analysis: *No explicit evidence of non-academic stakeholder involvement, engagement, or influence.*
Evaluation: *The community group members were involved in filling out surveys as part of the evaluation*
Dissemination: *No explicit evidence of non-academic stakeholder involvement, engagement, or influence.*

18. Nilvarangkul, McCann et al. 2011

Aim & Target population: *Enhancing health-related quality of life for Laotian migrants*

Inception: *Conceived by researchers with a broad action plan developed in collaboration with the target population*
Design: *The target population were involved in interviews used to shape the intervention; meetings were held with community workers and stakeholders to create action plans*
Implementation: *Community workers, stakeholders and researchers implemented the action plan*
Analysis: *No explicit evidence of non-academic stakeholder involvement, engagement, or influence.*
Evaluation: *Meetings were held with community workers and stakeholders as part of the formative evaluation; intervention participants were followed up via qualitative data collection*
Dissemination: *No explicit evidence of non-academic stakeholder involvement, engagement, or influence.*

19. Pinsker, Call et al. 2017

Aim & Target population: *Tobacco use prevention videos for Somali youth*

For peer review only

Inception: *No explicit evidence of non-academic stakeholder involvement, engagement, or influence.*
Design: *There was indirect involvement through focus groups of the target population; there was direct involvement of the target population in developing the videos, building upon the focus groups.*
Implementation: *Target population were involved in shaping the implementation of the intervention*
Analysis: *No explicit evidence of non-academic stakeholder involvement, engagement, or influence.*
Evaluation: *Target population evaluated the intervention through participant interviews*
Dissemination: *No explicit evidence of non-academic stakeholder involvement, engagement, or influence.*

20. Quandt, Grzywacz et al. 2013

Aim & Target population: *Pesticide safety intervention for migrant farmworkers*

Inception: *Based on an existing relationship between the university and community partners*
Design: *Based on an existing partnership; not immediately obvious how taget population and non-academic stakeholders were involved.*
Implementation: *Implemented by the community partners; lay health promoters were recruited from within the community and trained by the community partner*
Analysis: *No explicit evidence of non-academic stakeholder involvement, engagement, or influence.*
Evaluation: *No explicit evidence of non-academic stakeholder involvement, engagement, or influence.*
Dissemination: *No explicit evidence of non-academic stakeholder involvement, engagement, or influence.*

21. Solorio, Norton-Shelpuk et al. 2014

Aim & Target population: *HIV prevention messages for young Latino immigrant men who have sex with men*

Inception: *There is a previous community-university relationship, uncertain as to where the inception for the project came.*
Design: *Storyboard/intervention was put before the target group in focus groups, Latino MSM were hired to facilitate the focus groups and help interpret the findings*
Implementation: *Intervention was actioned by an external digital marketing/media team who used the design focus groups to develop the campaign*
Analysis: *Latino MSM were hired to help interpret the findings*
Evaluation: *The target population were involved in focus groups to assess their comprehension of the intervention*
Dissemination: *No explicit evidence of non-academic stakeholder involvement, engagement, or influence.*

22. Song, Han et al. 2010

Aim & Target population: *Translating dietary guidelines into a culturally tailored nutrition education program for Korean-American immigrants with type 2 diabetes*

Inception: *There is a previous relationship between researchers and community, but no clear link as to how this explicitly influenced inception*
Design: *No explicit evidence of non-academic stakeholder involvement, engagement, or influence.*
Implementation: *No explicit evidence of non-academic stakeholder involvement, engagement, or influence; appears the education program was delivered by nurses and dieticians*
Analysis: *No explicit evidence of non-academic stakeholder involvement, engagement, or influence.*

For peer review only

Evaluation: *Target population were involved in evaluating the interventions' acceptability*
Dissemination: *No explicit evidence of non-academic stakeholder involvement, engagement, or influence.*

23. Suarez-Balcazar, Early et al. 2018

Aim & Target population: *Healthy lifestyle promotion among Latino immigrant families with youth with disabilities*

Inception: *No explicit evidence of non-academic stakeholder involvement, engagement, or influence.*
Design: *Focus groups with the target population; interviews and conversations with community stakeholders and agency personnel; research built on by collaboration with mothers and staff from community organisations*
Implementation: *Delivered by bilingual occupational therapy and nutrition students; co-facilitated by mothers who presented Zumba sessions and prepared healthy snacks.*
Analysis: *No explicit evidence of non-academic stakeholder involvement, engagement, or influence.*
Evaluation: *Evaluated through focus groups at the end of the intervention*
Dissemination: *No explicit evidence of non-academic stakeholder involvement, engagement, or influence.*

24. Vaughn & Jacquez. 2019

Aim & Target population: *Stress and coping intervention for Latino immigrants*

Inception: *There are existing relations between the researchers and community; migrant community members were selected to be co-researcher*
Design: *Design input came from researchers and community co-researchers*
Implementation: *Community co-researchers implemented the intervention*
Analysis:
Evaluation: *Co-researchers provided evaluation through interviews with the researchers*
Dissemination: *No clear evidence, though does mention co-researchers want to be involved in dissemination including publications and presentations*

25. Wieland, Nigon. 2019

Aim & Target population: *Tuberculosis screening for migrant adults*

Inception: *Conceived and initiated by a community organisation*
Design: *Academic and non-academic community organisation co-designed the intervention; not sure of the involvement of the target population*
Implementation: *Intervention was implemented by community organisation staff, along with clinical/nursing staff and students*
Analysis: *Evidence was collected and analysed by local health practitioners*
Evaluation: *No explicit evidence of non-academic stakeholder involvement, engagement, or influence.*
Dissemination: *No explicit evidence of non-academic stakeholder involvement, engagement, or influence.*

26. Wieland, Njeru et al. 2017

Aim & Target population: *Digital storytelling intervention for immigrants and refugees with diabetes*

Inception: *Impetus came from an existing researcher-community partnership; diabetes co-agreed as an area for research and intervention*
Design: *survey developed by community-academic partnership to understand target population, attitudes and behaviours in T2DM; focus groups were conducted to further inform intervention*

For peer review only - http://bmjopen.bmj.com/site/about/guidelines.xhtml

content and to identify gifted storytellers; storytellers were recruited by community partners and their stories were captured, recorded and edited to derive the final intervention product.
Implementation: *No explicit evidence of non-academic stakeholder involvement, engagement, or influence.*
Analysis: *No explicit evidence of non-academic stakeholder involvement, engagement, or influence.*
Evaluation: *participants completed follow-up surveys*
Dissemination: *No explicit evidence of non-academic stakeholder involvement, engagement, or influence.*

27. Wieland, Wies. 2012

Aim & Target population: **Physical activity and nutrition program for immigrant and refugee women**

Inception: *Conceived from a community-academic partnership with long-standing ties; formalised to use CBPR to improve migrant health*
Design: *Exercise programme piloted; refugee and immigrant women were included as focus group participants*
Implementation: *Implemented by researchers and community organisation; community organisation recruited women to the programme; community organisation provided facilities*
Analysis: *No explicit evidence of non-academic stakeholder involvement, engagement, or influence.*
Evaluation: *No explicit evidence of non-academic stakeholder involvement, engagement, or influence.*
Dissemination: *No explicit evidence of non-academic stakeholder involvement, engagement, or influence.*

28. Williams, Ochsner et al. 2010

Aim & Target population: *Health and safety training for Latino day laborers*

Inception: *Inception appears to come from advocacy/community groups*
Design: *Focus groups held with the target population to identify typical exposures and situations to be addressed by peer trainers; curriculum was revised with feedback from these peer trainers*
Implementation: *Delivered by peer-trainers*
Analysis: *No explicit evidence of non-academic stakeholder involvement, engagement, or influence.*
Evaluation: *Evaluated through focus group discussions and pre & post-training surveys;*
Dissemination: *No explicit evidence of non-academic stakeholder involvement, engagement, or influence.*

For peer review only

PRISMA 2020 Checklist

Section and Topic	Item #	Checklist item	Location where item is reported
**TITLE**			
Title	1	Identify the report as a systematic review.	Title (pg.1); Abstract (pg.2)
**ABSTRACT**			
Abstract	2	See the PRISMA 2020 for Abstracts checklist.	Abstract (pg. 2-3)
**INTRODUCTION**			
Rationale	3	Describe the rationale for the review in the context of existing knowledge.	Introduction (pg.5)
Objectives	4	Provide an explicit statement of the objective(s) or question(s) the review addresses.	Introduction (pg.5-7)
**METHODS**			
Eligibility criteria	5	Specify the inclusion and exclusion criteria for the review and how studies were grouped for the syntheses.	Pg.7
Information sources	6	Specify all databases, registers, websites, organisations, reference lists and other sources searched or consulted to identify studies. Specify the date when each source was last searched or consulted.	Pg.8
Search strategy	7	Present the full search strategies for all databases, registers and websites, including any filters and limits used.	Additional file 1.
Selection process	8	Specify the methods used to decide whether a study met the inclusion criteria of the review, including how many reviewers screened each record and each report retrieved, whether they worked independently, and if applicable, details of automation tools used in the process.	Pg.8-9
Data collection process	9	Specify the methods used to collect data from reports, including how many reviewers collected data from each report, whether they worked independently, any processes for obtaining or confirming data from study investigators, and if applicable, details of automation tools used in the process.	Pg.9
Data items	10a	List and define all outcomes for which data were sought. Specify whether all results that were compatible with each outcome domain in each study were sought (e.g. for all measures, time points, analyses), and if not, the methods used to decide which results to collect.	Pg. 9-11
	10b	List and define all other variables for which data were sought (e.g. participant and intervention characteristics, funding sources). Describe any assumptions made about any missing or unclear information.	Pg. 9-11
Study risk of bias assessment	11	Specify the methods used to assess risk of bias in the included studies, including details of the tool(s) used, how many reviewers assessed each study and whether they worked independently, and if applicable, details of automation tools used in the process.	n/a – well recognised and validated tools are not available for participatory research
Effect measures	12	Specify for each outcome the effect measure(s) (e.g. risk ratio, mean difference) used in the synthesis or presentation of results.	n/a – did not include numerical outcomes such as risk ratio or mean

For peer review only - http://bmjopen.bmj.com/site/about/guidelines.xhtml

PRISMA 2020 Checklist

Section and Topic	Item #	Checklist item	Location where item is reported
			difference
Synthesis methods	13a	Describe the processes used to decide which studies were eligible for each synthesis (e.g. tabulating the study intervention characteristics and comparing against the planned groups for each synthesis (item #5)).	Pg. 9-11
	13b	Describe any methods required to prepare the data for presentation or synthesis, such as handling of missing summary statistics, or data conversions.	Pg. 9-11
	13c	Describe any methods used to tabulate or visually display results of individual studies and syntheses.	Pg. 9-11
	13d	Describe any methods used to synthesize results and provide a rationale for the choice(s). If meta-analysis was performed, describe the model(s), method(s) to identify the presence and extent of statistical heterogeneity, and software package(s) used.	Pg. 9-11
	13e	Describe any methods used to explore possible causes of heterogeneity among study results (e.g. subgroup analysis, meta-regression).	n/a – we did not explore heterogeneity
	13f	Describe any sensitivity analyses conducted to assess robustness of the synthesized results.	n/a – we did not perform sensitivity analyses as we did not include numerical outcomes that would be suitable
Reporting bias assessment	14	Describe any methods used to assess risk of bias due to missing results in a synthesis (arising from reporting biases).	n/a – we have no formal reporting bias assessment; we do discuss reporting issues in the discussion
Certainty assessment	15	Describe any methods used to assess certainty (or confidence) in the body of evidence for an outcome.	n/a – there are no definitive methods for assessing certainty around the data we utilise. We do discuss issues

For peer review only

PRISMA 2020 Checklist

Section and Topic	Item #	Checklist item	Location where item is reported
			around variability in terms, application of methods and lack of reporting.
**RESULTS**			
Study selection	16a	Describe the results of the search and selection process, from the number of records identified in the search to the number of studies included in the review, ideally using a flow diagram.	Figure 1 (pg.15); pg.14
	16b	Cite studies that might appear to meet the inclusion criteria, but which were excluded, and explain why they were excluded.	Figure 1 details reasons for exclusion.
Study characteristics	17	Cite each included study and present its characteristics.	Table 3 (pg.16-19)
Risk of bias in studies	18	Present assessments of risk of bias for each included study.	n/a - there are no definitive methods for assessing certainty around the data we utilise. We do discuss issues around variability in terms, application of methods and lack of reporting.
Results of individual studies	19	For all outcomes, present, for each study: (a) summary statistics for each group (where appropriate) and (b) an effect estimate and its precision (e.g. confidence/credible interval), ideally using structured tables or plots.	Table 2 & table 4. There are no numerical outcomes to report or synthesise
Results of	20a	For each synthesis, briefly summarise the characteristics and risk of bias among contributing studies.	Pg. 19-23

For peer review only - http://bmjopen.bmj.com/site/about/guidelines.xhtml

PRISMA 2020 Checklist

Section and Topic	Item #	Checklist item	Location where item is reported
syntheses	20b	Present results of all statistical syntheses conducted. If meta-analysis was done, present for each the summary estimate and its precision (e.g. confidence/credible interval) and measures of statistical heterogeneity. If comparing groups, describe the direction of the effect.	n/a
	20c	Present results of all investigations of possible causes of heterogeneity among study results.	n/a – we did not examine heterogeneity
	20d	Present results of all sensitivity analyses conducted to assess the robustness of the synthesized results.	n/a – there were no numerical outcomes on which to perform sensitivity analysis
Reporting biases	21	Present assessments of risk of bias due to missing results (arising from reporting biases) for each synthesis assessed.	n/a - we have no formal reporting bias assessment; we do discuss reporting issues in the discussion
Certainty of evidence	22	Present assessments of certainty (or confidence) in the body of evidence for each outcome assessed.	n/a – there are no definitive methods for assessing certainty around the data we utilise. We do discuss issues around variability in terms, application of methods and lack of reporting.
**DISCUSSION**			
Discussion	23a	Provide a general interpretation of the results in the context of other evidence.	Pg. 26-31
	23b	Discuss any limitations of the evidence included in the review.	Pg. 31-32

For peer review only

PRISMA 2020 Checklist

Section and Topic	Item #	Checklist item	Location where item is reported
	23c	Discuss any limitations of the review processes used.	Pg. 31-32
	23d	Discuss implications of the results for practice, policy, and future research.	Pg. 30-31; 33 (conclusion)
**OTHER INFORMATION**			
Registration and protocol	24a	Provide registration information for the review, including register name and registration number, or state that the review was not registered.	Pg. 3 (abstract); pg. 7
	24b	Indicate where the review protocol can be accessed, or state that a protocol was not prepared.	Pg. 3 (abstract); pg. 7
	24c	Describe and explain any amendments to information provided at registration or in the protocol.	n/a
Support	25	Describe sources of financial or non-financial support for the review, and the role of the funders or sponsors in the review.	Pg. 34
Competing interests	26	Declare any competing interests of review authors.	Pg. 34
Availability of data, code and other materials	27	Report which of the following are publicly available and where they can be found: template data collection forms; data extracted from included studies; data used for all analyses; analytic code; any other materials used in the review.	Pg. 34

From: Page MJ, McKenzie JE, Bossuyt PM, Boutron I, Hoffmann TC, Mulrow CD, et al. The PRISMA 2020 statement: an updated guideline for reporting systematic reviews. BMJ 2021;372:n71. doi: 10.1136/bmj.n71

For more information, visit: http://www.prisma-statement.org/

For peer review only

BMJ Open

Participatory approaches in the development of health interventions for migrants: a systematic review

Field	Value
Journal:	*BMJ Open*
Manuscript ID	bmjopen-2021-053678.R2
Article Type:	Original research
Date Submitted by the Author:	29-Sep-2021
Complete List of Authors:	Rustage, Kieran; St George's University of London, Institute for Infection and Immunity<br>Crawshaw, Alison; St George's University of London, Institute for Infection and Immunity<br>Majeed-Hajaj, Saliha; Queen Mary University of London<br>Deal, Anna; St George's University of London, Institute for Infection and Immunity; London School of Hygiene & Tropical Medicine<br>Nellums, Laura ; University of Nottingham, Division of Epidemiology and Public Health<br>Ciftci, Yusuf; Doctors of the World<br>Fuller, Sebastian; St George's University of London, Institute for Infection and Immunity<br>Goldsmith, Lucy; St George's University of London, Institute for Infection and Immunity, and Population Health Research Institute;<br>Friedland, Jon; St George's University of London<br>Hargreaves, Sally; St George's University of London, Institute for Infection and Immunity
**Primary Subject Heading**:	Global health
Secondary Subject Heading:	Public health, Patient-centred medicine
Keywords:	PUBLIC HEALTH, STATISTICS & RESEARCH METHODS, EDUCATION & TRAINING (see Medical Education & Training)

SCHOLARONE™
Manuscripts

I, the Submitting Author has the right to grant and does grant on behalf of all authors of the Work (as defined in the below author licence), an exclusive licence and/or a non-exclusive licence for contributions from authors who are: i) UK Crown employees; ii) where BMJ has agreed a CC-BY licence shall apply, and/or iii) in accordance with the terms applicable for US Federal Government officers or employees acting as part of their official duties; on a worldwide, perpetual, irrevocable, royalty-free basis to BMJ Publishing Group Ltd ("BMJ") its licensees and where the relevant Journal is co-owned by BMJ to the co-owners of the Journal, to publish the Work in this journal and any other BMJ products and to exploit all rights, as set out in our licence.

The Submitting Author accepts and understands that any supply made under these terms is made by BMJ to the Submitting Author unless you are acting as an employee on behalf of your employer or a postgraduate student of an affiliated institution which is paying any applicable article publishing charge ("APC") for Open Access articles. Where the Submitting Author wishes to make the Work available on an Open Access basis (and intends to pay the relevant APC), the terms of reuse of such Open Access shall be governed by a Creative Commons licence – details of these licences and which Creative Commons licence will apply to this Work are set out in our licence referred to above.

Other than as permitted in any relevant BMJ Author's Self Archiving Policies, I confirm this Work has not been accepted for publication elsewhere, is not being considered for publication elsewhere and does not duplicate material already published. I confirm all authors consent to publication of this Work and authorise the granting of this licence.

For peer review only

Participatory approaches in the development of health interventions for migrants: a systematic review

Kieran Rustage,[1] Alison Crawshaw,[1] Saliha Majeed-Hajaj,[2] Anna Deal,[1] Laura B Nellums,[3] Yusuf Ciftci,[4] Sebastian Fuller,[1] Lucy Goldsmith,[1] Jon S Friedland,[1] Sally Hargreaves[1]

Corresponding author: Sally Hargreaves (s.hargreaves@sgul.ac.uk)

Word count: 4760

Key Words: Participatory Research; Systematic Review; Migrant Health; Health Intervention

Institutional affiliations:

1. The Migrant Health Research Group, Institute for Infection & Immunity, St George's University of London, United Kingdom
2. Queen Mary University of London, London, United Kingdom
3. University of Nottingham, Nottingham, United Kingdom
4. Doctors of the World UK, part of the Médecins du Monde network

For peer review only

For peer review only - http://bmjopen.bmj.com/site/about/guidelines.xhtml

Abstract

Objective

Analysis of participatory approaches to developing health interventions for migrants, and how approaches embody core participatory principles of inclusivity and democracy.

Design

A systematic review of original articles. Electronic searches within the databases MEDLINE, Embase, Global Health and PsychINFO (from inception – Nov 2020).

Eligibility criteria for study selection

Original peer-reviewed articles reporting research to develop and implement a health intervention for migrants, incorporating participatory approaches. We defined migrants as foreign-born individuals. Only articles reporting the full research cycle (inception, design, implementation, analysis, evaluation, dissemination) were included.

Data extraction

We extracted information related to who was involved in research (migrants or other non-academic stakeholders), the research stage at which they were involved (inception, design, implementation, analysis, evaluation, dissemination), the method of their involvement, and how this aligned with the core principles of participatory research – categorising studies as exhibiting active or pseudo (including proxy and indirect) participation.

Results

1793 publication were screened of which 28 were included in our analysis. We found substantial variation in the application of participatory approaches in designing health interventions targeting migrants: across 168 individual research stages analysed across the 28 studies, we recorded 46 instances of active participation of migrants, 30 instances of proxy participation, and 24 instances of

For peer review only - http://bmjopen.bmj.com/site/about/guidelines.xhtml

indirect participation. all studies involved non-academic stakeholders in at least one stage of the research, only two studies exhibited evidence of active participation of migrants across all research stages. Evidence is limited due to the variability of terms and approaches used.

Conclusions

Important shortfalls in the meaningful inclusion of migrants in developing health interventions exists, suggesting a more rigorous and standardised approach is warranted to better define and deliver participatory research and improve quality.

Registration

This review followed PRISMA guidelines and is registered on the Open Science Framework (osf.io/2bnz5).

Strengths and Limitations

For peer review only - http://bmjopen.bmj.com/site/about/guidelines.xhtml

- This systematic review represents a robust and novel assessment of the application of participatory approaches and principles toward research into health interventions involving migrants.
- This review casts a critical lens over the relationship between how participatory approaches are applied, and how participatory principles such as inclusivity and democracy are embodied.
- Due to the varied and interchangeable use of participatory research terms the categorisations and definitions we use could be interpreted differently by others.
- This review is limited by the lack of clear and consistent reporting of participatory methods used, suggesting that guidelines must be developed and more consistently adopted to improve transparency in all participatory research.
- This review does not address possible associations between participatory methods and final health or research outcomes, which should be better considered in future research.

Introduction

For peer review only - http://bmjopen.bmj.com/site/about/guidelines.xhtml

Considerable emphasis is now being placed on ensuring patient and public engagement in health research, including striving for greater involvement of marginalised groups such as migrants and ethnic minorities.[1,2] However, whether this is effectively and meaningfully done in practice to ensure truly patient-centred research has yet to be fully elucidated. Participatory research represents a distinct research paradigm in which research is done collaboratively with the individuals whose lived experiences and actions are the subject of study, as active partners who share power and influence over research processes and outcomes.[3-6]

Two fundamental principles of participatory research that underpin the ability for stakeholders to effectively co-operate and share power are those of inclusivity and democracy, particularly in relation to those directly affected by the research in question.[3] That is to say, has the research included the individuals the research would otherwise be about, and have these individuals, during their inclusion, had influence or power over research decisions on par with the research professionals? Included under the umbrella of participatory research approaches are more specific methodologies which look to uphold these principles, including: community based participatory research (CBPR)[7]; action research[8]; some patient & public involvement (PPI)[9]; as well as broader derivatives such as community-based collaborative action research. Participatory research holds the potential to bridge the gap between public health research and practice, creating a context in which patients and the public have meaningful influence over research decisions, increasing the relevance and impact of research outcomes to their own lives.[10]

Participatory research is likely to be particularly powerful when working with underserved and marginalised groups such as migrants, where traditional research has frequently failed to provide an appreciable health benefit. Whilst a heterogenous group, comprising a multitude of cultures, ethnicities and socio-cultural circumstances, many migrants can find themselves in vulnerable situations, marginalised by health systems,[11,12] and society alike.[13,14] There is a growing consensus around the need for academics and health systems to become more responsive to, and inclusive of

For peer review only - http://bmjopen.bmj.com/site/about/guidelines.xhtml

refugee and migrant health concerns.[15] Indeed, limited community engagement in public health interventions has already been shown to be effective when working with marginalised groups around a range of health outcomes, and can provide benefits to participants themselves, such as in improving health behaviours and participant self-efficacy.[16] However, the ultimate goal is to conduct participatory research with migrants as a matter of routine, so that research is better centred around, and grounded in the needs of migrant communities.

Despite the potential of participatory research, there are varied interpretations as to how to apply such approaches. A review of peer models in participatory research, in which partnerships with 'insiders' are established reveal a norm in which practices and terms are interchangeable, and inconsistently applied.[17] Challenges exist in deciding who should be involved, and whether involvement should extend beyond the target group (in this case, migrants), to other non-academic stakeholder groups such as community organisations and professionals.[3,17] There are also differing interpretations of the degree of participation required of individuals for research to be considered participatory rather than tokenistic, though it is suggested that unless involved individuals are partners or co-researchers throughout the entirety of a project, the work cannot be participatory.[3,18] Overall, it is widely agreed quality participation is characterised by non-academic stakeholders having opportunities to engage with, make decisions about, and perform leadership roles around such research,[5] empowering the public at the highest level and asserting their right to be involved in decision-making and to influence outcomes. Understanding the different approaches to participatory research and whether the core principles of participatory research are upheld is crucially important if good practice is to be identified.

We therefore did a systematic review to analyse participatory approaches in the development of health interventions for migrants, through use of a framework which relates categories of participation to core principles of participation (inclusivity and democracy), and collates evidence of

For peer review only - http://bmjopen.bmj.com/site/about/guidelines.xhtml

the benefits of utilising a participatory approach to research, and of the challenges of utilising a participatory approach to research.

For peer review only

For peer review only - http://bmjopen.bmj.com/site/about/guidelines.xhtml

Methods

We did a systematic review, following PRISMA guidelines, which is registered on the Open Science Framework (osf.io/2bnz5). The primary aim of this systematic review was to analyse the use of participatory approaches to develop health interventions targeting migrants as the intended beneficiaries. Specifically, we established a framework of categories of participation, which related the data we extracted to participatory principles of inclusivity and democracy. Our secondary aims were to describe the challenges and benefits of utilising participatory approaches experienced in the research process.

Inclusion and Exclusion criteria

We included peer-reviewed primary-research reporting on health research into interventions aimed at benefitting migrant populations that described using a participatory approach across the whole research process. Research which purported to use a participatory research approach through descriptors in their introduction and methods, or which utilised a recognised participatory approach such as community-based participatory research (CBPR), action research, or community-based collaborative action research, and specifically targeted migrants, was included in the review. We defined migrants as foreign-born individuals and considered a health intervention to be any initiative, tool, or programme that looked to improve health outcomes, including those related to mental health and health literacy.

Studies were excluded if they did not report on all stages of research into the health intervention: Inception, design, implementation, analysis, evaluation, dissemination.
As such, publications presenting interim results of studies which had not completed the full research cycle, as well as studies specifically focusing on only co-designing interventions were excluded. We took this approach so as not to unfairly penalise ongoing research in our analysis, nor co-designed

For peer review only - http://bmjopen.bmj.com/site/about/guidelines.xhtml

research; we consider co-design to be one component of the broader participatory research paradigm and were most interested in how approaches manifest across the entirety of a research cycle. Studies explicitly defining migrant status according to ethnic or ancestral background but not country of birth were excluded, as were papers where primary data were not reported (e.g., comments, editorials, letters, and reviews).

Search strategy

We individually searched the databases MEDLINE (1946 – November 2020), Embase (1974 – November 2020), Global Health (1910 – November 2020) and PsychINFO (1967 – November 2020) within the Ovid platform utilising a Boolean search strategy with keywords and medical subheadings (MeSH) related to two major themes: migrants and participatory research. There were no geographic or language restrictions. An additional text file outlines the full searches carried out (see Additional file 1). The retrieved hits from each database were combined and deduplicated manually within Rayyan. We identified additional studies through hand searching the bibliographies of publications included after full-text screening.

Study selection

Two reviewers duplicated the title and abstract screening and full-text screening (KR & SM-H), which was carried out using the web-based application Rayyan.[19] The reasons for excluding studies during full-text screening were recorded. Any discrepancies in screening decision between the two initial reviewers were mediated by a third reviewer (AC). Where retrieved articles indicated the existence of a separate methodological article, we also screened this in conjunction with the first article on condition that it was a retrospective report of methods used (across all stages of the research) rather than a prospective outline of planned methods.

Data extraction and analysis

For peer review only - http://bmjopen.bmj.com/site/about/guidelines.xhtml

Studies which reported using participatory research approaches, and which reported on all stages of the research were extracted using a piloted form by KR and SH. We extracted summary data on geographical location, the self-described participatory approach, specific target population, and aims of the research. Data relating to the participatory approach of each study was extracted and analysed to achieve our primary aim. We extracted data on the stages of the research in which migrants were involved (Table 1). Where specific research stages did not involve migrants, but did involve other non-academic stakeholders this was recorded, sub-categorising these groups as community groups/third-sector organisations or professional services. We subsequently extracted data on the methods used to involve migrants (or other non-academic stakeholders) at each stage of the research (inception, design, implementation, analysis, evaluation, and dissemination). An additional file provides details of the summary extracted data (see Additional file 2).

For peer review only - http://bmjopen.bmj.com/site/about/guidelines.xhtml

Table 1. Stages of research and evidence sought for each stage during data extraction

	Research Stages					
	Inception	Design	Implementation	Analysis	Evaluation	Dissemination
Evidence Sought	Who was responsible for having the idea for the research, or initiating the study?	Who was involved in the initial planning and design of the intervention/study? Who decided what the final design of the intervention/study would be?	Who was responsible for implementing/piloting the intervention within the remit of the study? Who decided what this implementation should look like?	Who was involved in analysing data relating to primary endpoints/outcomes? Who decided what these endpoints or outcomes should be?	Who was involved in the overall evaluation of the intervention/study? For example, process evaluation, reflective evaluation? Who had a say in determining successes/failures/future considerations?	Who was involved in disseminating findings? What form did this take? Who decided this?

For peer review only - http://bmjopen.bmj.com/site/about/guidelines.xhtml

We related extracted information on who was involved, when they were involved, and how they were involved in the described research, relating these factors to participatory principles of inclusion and democracy and categorising them within a framework we developed (Table 2). The framework was developed with reference to the literature, particularly that relating to participatory research as a democratic process and being necessary to implement at all stage of the research.[3,5,18,20] . We used data extracted as to who was involved, and when, to guide our assessment of inclusivity. Specifically, we were concerned with whether the evidence displayed relevant inclusivity, that is, the involvement of migrant individuals that are the target, or intended beneficiary of the health intervention. We used data extracted as to the method and means of involvement to guide our assessment of democracy. In this instance we sought evidence of whether methods employed in the research suggested greater levels of democracy, such as through power-sharing and decision-making mechanisms such as equal voting, or committees for those involved. Within the framework we categorised the aggregated data from each study, with specific reference to migrant individuals, as: Active participation; Pseudo Participation (including proxy and indirect participation); or No explicit evidence of participation. The final framework and definitions were agreed by all co-authors (table 2).

To achieve our secondary aim, we specifically scanned the included articles for evidence of any evaluation of the use of participatory approaches within the research, inclusive of reflections that appeared in the discussion of included articles. We extracted this data, where found, and categorised it as representing a challenge or benefit associated with the use of participatory approaches toward the overall research process.

Patient and Public Involvement

For peer review only - http://bmjopen.bmj.com/site/about/guidelines.xhtml

Members of our authorship team have past and current experience of working within third sector organisations. This experience helped to frame the research questions, and definitions used in the analysis. However, lay-patients and public specifically, were not involved in this research.

For peer review only - http://bmjopen.bmj.com/site/about/guidelines.xhtml

Table 2. Framework of the category of participation, with definitions, criteria, and relationships to participatory principles applied to aggregated data extracted in this review.

Category of Participation		Definition	Criteria	Relationship to participatory principles
Active Participation		Migrants appeared to be both actively involved, and wielded influence in decisions relating to the research.	• Migrant individuals were involved in this stage of the research.<br>&<br>• Individuals involved appeared to have direct power and influence over the research stage through shared processes with researchers such as voting, or committees.	Relevant inclusivity & greater democracy with regards to migrant involvement.
Pseudo-participation	Proxy-participation	Community/third-sector organisations and/or professional services are actively involved and wield influence in decisions related to the research stage ahead (or in lieu) of migrants.	• There is uncertain/no clear evidence migrant individuals were involved.<br>Or<br>• Where migrant individuals were involved, they did not appear to have direct power and influence over the research through shared processes with researchers such as voting, or committees. Rather, they were appeared to be involved as research subjects, in surveys or focus groups.<br>But<br>• Third-sector organisations and/or professional services were involved.<br>&<br>• Third-sector organisation and/or professional services appeared to have direct power and influence over the research stage through	There may be relevant inclusivity, but lesser democracy with regards to migrant involvement.<br><br>There may be greater democracy with other stakeholders.

For peer review only

			shared processes with researchers such as voting, or committees.	
	Indirect participation	Migrants' involvement is restricted to activities in which they are research subjects (surveys, focus groups, interviews). No other stakeholders appear to be involved.	• Where migrant individuals were involved, they did not appear to have direct power and influence over the research through shared processes with researchers such as voting, or committees. Rather, they appeared to be involved as research subjects, in surveys or focus groups.<br>&<br>• There is uncertain/no clear evidence as to the involvement of third-sector organisations or professional services.	There may be relevant inclusivity, but lesser democracy with regards to migrant involvement.<br><br>There is no clear evidence of inclusivity or democracy with other stakeholders.
No explicit evidence		No non-academic stakeholders (migrant or otherwise) appear to be involved in this stage of the research.	• There is no clear evidence as to the involvement of migrant individuals.<br><br>• There is no clear evidence as to the involvement of third-sector organisations or professional services.	There is no clear evidence of inclusivity or democracy with migrants or other stakeholders.

For peer review only - http://bmjopen.bmj.com/site/about/guidelines.xhtml

Results

Screening results

Database searches returned 1793 results; a total of 292 duplicates were removed, and 1501 publications were retained for title and abstract screening. Of the 1501 remaining publications, 1357 were excluded during title and abstract screening, and 144 were retained for full-text screening. During full-text screening, 116 publications did not meet our criteria and were excluded, with the reasons for exclusion recorded (Fig 1). Overall, 28 publications met the inclusion criteria and were included in this systematic review (Table 3).

For peer review only

For peer review only - http://bmjopen.bmj.com/site/about/guidelines.xhtml

Table 3. Descriptive characteristic of studies included in this systematic review.

Citation	***Location***	***Self-described participatory approach/methodology***	***Specific Target population***	***Aim of the health intervention***
Afifi, Makhoul. 2011	*Lebanon*	Community-Based Participatory Research (CBPR)	*Palestinian refugee youth*	*Mental health promotion*
Aitaoto, Braun. 2012	*United States*	Community-Based Participatory Research (CBPR)	*Micronesian women*	*Cancer outreach/education*
Baird, Domian. 2015	*United States*	*Community-based collaborative-action research*	*Sudanese Refugee women*	*Addressing health challenges associated with relocation*
Barbee, Kobetz. 2010	*United States*	Community-Based Participatory Research (CBPR)	*Haitian immigrant women*	*To assess the acceptability of HPV self-sampling with community health workers to detect cervical cancer*
Chesla, Chun. 2013	*United States*	Community-Based Participatory Research (CBPR)	*Chinese-American Immigrants*	*To culturally adapt coping skills training for type-2 diabetes*
Evans, suggs. 2019	*United Kingdom*	Community-Based Participatory Research (CBPR)	*African migrants*	*To promote HIV testing uptake*
Forst, Ahonen. 2013	*United States*	Community-Based Participatory Research (CBPR)	*Hispanic construction workers*	*To increase awareness of workplace hazards and self-efficacy; expansion of worker centre agenda to include occupation health and safety*
Goodkind, Amer. 2017	*United States*	Community-Based Participatory Research (CBPR)	*Afghan, Great lakes Region African, and Iraqi refugee adults*	*To address social determinants of health; to improve linkage to mental health services and retention in trauma-focused treatment*
Grigg-Saito, Och. 2008	*United States*	*Community-Based Outreach*	*Cambodian immigrants*	*Strength-based outreach to eliminate cardiovascular disease and diabetes disparities*
Henderson, Slater.	*Canada*	*Action Research*	*Newly arrived migrants*	*To provide tailored nutritional information and support*
Jacquez, Vaughn. 2019	*United States*	Community-Based Participatory Research (CBPR)	*Latino immigrants*	*Stress reduction*
Kaiser, Martinez. 2015	*United States*	Community-Based Participatory Research (CBPR)	*Mexican immigrants*	*To provide obesity prevention education & outreach*
Kandula, Dave. 2016	*United States*	Community-Based Participatory Research (CBPR)	*South Asian immigrant women*	*Exercise intervention for those at risk of diabetes*
Karasz, Raghavan. 2015	*United*	Community-Based Participatory Research	*Bangladeshi immigrant women*	*To provide and intervention tackling common mental disorders*

For peer review only - http://bmjopen.bmj.com/site/about/guidelines.xhtml

	States	(CBPR)		
Kim, Koniak-Griffin. 2004	*United States*	Community-Based Participatory Research (CBPR)	*Latino immigrants*	*To utilise Lay health advisors for cardiovascular health promotion*
Lam, Mcphee. 2003	*United States*	Community-Based Participatory Research (CBPR)	*Vietnamese immigrants*	*To increase pap screening through education and outreach through lay health workers and media*
Li, Yeh. 2013	*United States*	Community-Based Participatory Research (CBPR)	*Chinese-American immigrants*	*To prevent colorectal cancer through education and outreach*
Nilvarangkul, McCann. 2011	*Thailand*	*Action Research*	*Laotian migrants*	*Enhancement of a quality-of-life model*
Pinsker, Call. 2016	*United States*	Community-Based Participatory Research (CBPR)	Somali youth	*To provide a culturally appropriate smoking cessation intervention*
Quandt, Grzywacz. 2014	*United States*	Community-Based Participatory Research (CBPR)	*Latino immigrants*	*To provide Lay health promoter-led pesticide safety education*
Solorio, Norton-Shelpuk. 2014	*United States*	Community-Based Participatory Research (CBPR)	*Latino immigrant MSM*	*To provide HIV prevention outreach for men who have sex with men*
Song, Han. 2010	*United States*	Community-Based Participatory Research (CBPR)	*Korean-American immigrants*	*To translate current dietary guidelines into a culturally tailored nutrition program*
Suarez-Balcazar, Early. 2018	*United States*	Community-Based Participatory Research (CBPR)	*Latino immigrant families with youth with disabilities*	*To provide healthy lifestyle promotion*
Vaughn, Jacquez. 2019	*United States*	Community-Based Participatory Research (CBPR)	*Latino immigrants*	*To reduce stress and increase coping skills*
Wieland, Nigon. 2019	*United States*	Community-Based Participatory Research (CBPR)	*Foreign-born*	*To promote Tuberculosis screening*
Wieland, Njeru. 2017	*United States*	Community-Based Participatory Research (CBPR)	*Immigrants and refugees with T2DM*	*To provide a digital story-telling intervention to improve management of type-2 diabetes among those affected*
Wieland, Wies. 2012	*United States*	Community-Based Participatory Research (CBPR)	*Immigrant and refugee women*	*To provide a physical activity and nutrition program*
Williams, Ochsner. 2010	*United States*	Community-Based Participatory Research (CBPR)	*Latino immigrants*	*Health and safety education in construction*

For peer review only

Study Characteristics

The research articles included in this systematic review were published between 2003 and 2019. Only 13 of the publications had any discernible dates relating to when the reported work was conducted, with these dates being between 2003 and 2018. The majority of the publications related to work carried out in the United States (24 out of 28); the remaining publications related to work carried out in Canada,[21] Lebanon,[22] Thailand,[23] and the United Kingdom.[24] The self-described approach taken by 24 of the 28 included studies was community-based participatory research (CBPR)[22,24-46]; the remaining four studies described using community-based collaborative-action research,[47] community-based outreach,[48] and action research.[21,23] The dominant focus of the included studies was around education or outreach (Table 3), for example around cancer education,[25] or healthy lifestyles promotion;[41] five studies specifically mentioned including refugees (Table 3).[22,29,44,45,47]

Analysis of participatory approaches in research to develop health interventions for migrants

In our analysis, participation varied substantially according to the stage of the research under scrutiny. Only two of the included studies reported explicit evidence of some degree of participation of at least one non-academic stakeholder groups across all research stages (Table 4).[29,30] Overall, we extracted and categorised evidence of the participation of at least one non-academic stakeholder group in 22 studies during the inception;[21-23,25,26,29-34,36,38-40,42-48] 25 studies during the design;[21-23,25-39,41-46,48] 23 studies during implementation;[23-32,34,35,37-43,45-48] 4 studies during analysis;[29,30,39,43] 22 studies during evaluation;[21,23,24,27-37,39-42,44,46-48] and 4 studies during dissemination (Table 4).[26,29,30,42]

For peer review only - http://bmjopen.bmj.com/site/about/guidelines.xhtml

Table 4. Analysis and categorisation of participatory character displayed across research stages within included studies

Citation	*Research Stage*					
	Inception	*Design*	*Implementation*	*Analysis*	*Evaluation*	*Dissemination*
Afifi, Makhoul. 2011	○	◆	X	X	X	X
Aitaoto, Braun. 2012	●	◆	◆	X	X	X
Baird, Domian. 2015	◆	X	○	X	○	X
Barbee, Kobetz. 2010	◆	◆	◆	X	X	◆
Chesla, Chun. 2013	X	◆	●	X	○	X
Evans, suggs. 2019	X	X	○	X	○	X
Forst, Ahonen. 2013	X	●	●	X	◆	X
Goodkind, Amer. 2017	◆	◆	◆	◆	◆	◆
Grigg-Saito, Och. 2008	◆	◆	◆	X	◆	X
Henderson, Slater.	●	○	X	X	○	X
Jacquez, Vaughn. 2019	◆	◆	◆	◆	◆	◆
Kaiser, Martinez. 2015	○	◆	○	X	○	X
Kandula, Dave. 2016	●	●	●	X	●	X
Karasz, Raghavan. 2015	●	◆	X	X	○	X
Kim, Koniak-Griffin. 2004	●	◆	◆	X	◆	X
Lam, Mcphee. 2003	X	◆	◆	X	◆	X
Li, Yeh. 2013	●	●	X	X	○	X
Nilvarangkul, McCann. 2011	◆	◆	●	X	●	X
Pinsker, Call. 2016	X	◆	◆	X	○	X
Quandt, Grzywacz. 2014	●	●	●	X	X	X
Solorio, Norton-Shelpuk. 2014	●	○	●	○	○	X
Song, Han. 2010	●	X	●	X	○	X
Suarez-Balcazar, Early. 2018	X	◆	◆	X	○	X
Vaughn, Jacquez. 2019	◆	◆	◆	X	○	◆

For peer review only - http://bmjopen.bmj.com/site/about/guidelines.xhtml

Wieland, Nigon. 2019	●	●	●	●	X	X
Wieland, Njeru. 2017	●	○	X	X	○	X
Wieland, Wies. 2012	●	○	●	X	X	X
Williams, Ochsner. 2010	●	◆	◆	X	○	X

◆ **Active participation:** Migrants appeared to be both actively involved, and wielded influence in decisions relating to the research.

● **Proxy-participation:** Community/third-sector organisations and/or professional services are actively involved and wield influence in decisions related to the research stage ahead (or in lieu) of migrants.

○ **Indirect participation**: Migrants' involvement is restricted to activities in which they are research subjects (surveys, focus groups, interviews). No other stakeholders appear to be involved.

X **No explicit evidence:** No non-academic stakeholders (migrant or otherwise) appear to be involved in this stage of the research.

For peer review only

However, there was greater variation and divergence in participatory approaches when considering the degree of participation of migrants. In our analysis only 18 of the 28 included studies exhibit active participation of migrants (as the primary focus and target of the intervention) at any stage of the research process.[22,23,25-31,33-35,37,41,42,46-48] Of these 18 studies, only two display evidence of active participation of migrants at all stages of the research process.[29,30]

Across all 168 individual research stages analysed across the 28 studies, we recorded 46 instances of active participation of migrants; 30 instances of proxy participation; 24 instances of indirect participation; and 68 instances in which there was insufficient evidence to make a determination (Table 4). The active participation recorded also appears to be associated with the stage of the research. There were 7 instances of active participation during study inception;[23,26,30,42,47-49] 16 during design;[22,23,25-27,30,31,33-35,37,41,42,46,48,49] 10 during implementation;[25,26,30,34,35,37,41,42,46,48,49] 2 during analysis;[30,49] 6 during evaluation;[28,30,34,35,48,49] and 4 during dissemination.[26,30,42,49]

Evidence of the benefits of utilising a participatory approach to research

The benefit most often reported among the included articles in utilising participatory approaches was the assertion that interventions were better tailored to the target population through involving non-academic stakeholders.[22,25,27,32,37,41,42,46,50] This included two studies which spoke of the benefits of participatory research in facilitating interventions going beyond more immediately actionable cultural adaptations (such as language-adaptation and ethnically matched providers), to providing interventions that more deeply reflect community values and priorities.[27,42]

Participatory approaches provided benefits through the partnerships established during the research. One study reported how participatory approaches allowed for the modification of the research programme throughout conception, development, and implementation.[34] Multiple publications provided evidence on how iterative feedback from stakeholders during the studies

For peer review only - http://bmjopen.bmj.com/site/about/guidelines.xhtml

could further grow partnerships, improving the recruitment of individuals to implement or take part in the intervention,[21,22,27,32,42,51] and dissemination.[27] One study also highlighted that partnerships were a feasible and appropriate means to support intervention implementation,[24] whilst one set of authors reported that partnerships with non-academics can ultimately strengthen research.[26]

Better relationships between the community and academics were cited as having the capability to enhance the familiarity and trust of individuals involved in participatory research. One study cited that increased trust had direct benefits to research, leading to more open and honest dialogue than in traditional research, improving the accuracy and findings of these activities.[47] Researchers becoming part of ongoing community relations was seen as positive, or a catalyst, acting as an impartial bridge between disparate community groups.[22] Long-lasting partnerships built over the course of participatory research studies was cited as producing a capacity building element, increasing the health-related knowledge and resources of the community which academics partnered with.[28,35,43] Finally, partnerships catalysed a greater degree of understanding of a subject among communities, leading to increased self-determination and the ability to generate change of their own accord.[47]

Evidence of the challenges of utilising a participatory approach to research

Multiple studies highlighted the importance of balancing the culture and expectations of both researchers and migrant individuals to enact participatory research.[22,27,39] For example, one study reported that reaching equitability in the research process and working on level-terms with migrants directly conflicted with the cultural norms of some of these individuals, who may revere authority figures, and so would in normal circumstances defer to their judgement.[47] A further study highlighted challenges exist in bringing together differing stakeholders with varied views and experiences. In these situations it was suggested there is no “one size fits all” approach and that processes must be adapted to individual groups.[35] Noting varied perspective, one study highlights

For peer review only - http://bmjopen.bmj.com/site/about/guidelines.xhtml

the challenge that divergent perspectives as to what is most salient and important to address amongst those involved can present a challenge.[29]

The challenge (and importance) of building rapport and addressing mistrust,[22,23,48] or even research fatigue amongst some groups,[22] was also evident within publications. One set of authors, spoke not just of the need for non-academic stakeholders to trust researchers, but how It is also essential for researchers to reciprocate this trust, and prioritise the collaborative and democratic aim of participatory methods; this was perceived as challenging as it may shift the power dynamic and locus of control in the research away from the academics.[38] Even when partnerships overcome challenges of culture, expectations and trust, there remain other practical challenges to operationalising these partnerships.[33,37]

Challenges in ensuring equitability in research understanding, and balancing the participatory nature of a project, with the standards expected by the wider scientific community were also highlighted.[22,35] Furthermore, one study cited the difficulty of navigating acknowledgement and authorship of non-academics in published materials;[27]a scenario which serves to reiterate power-imbalances that can often persist,[52] in that despite being 'equal partners' in research, migrants may still not be equally recognised. The lack of recognition of the requirements of participatory research in traditional academic circles is also cited as a challenge, with one set of authors stating the need for managerial, institutional, and funder-level buy-in and commitment regarding participatory research.[22] Similarly, institutional review limited participation in at least one study, preventing non-academic stakeholders involvement in data collection and analysis.[35]

Other practical challenges to operationalising participatory research included effective, timely communication,[23,36] and the challenge of working with communities in which the dominant language of the researchers and migrant communities differ.[39,47] Finally, the iterative and tailored nature of

For peer review only - http://bmjopen.bmj.com/site/about/guidelines.xhtml

the interventions produced may also impact on the generalisability of findings,[41] whilst some work could seemingly omit or contradict research evidence due to localising the intervention.[44]

For peer review only

For peer review only - http://bmjopen.bmj.com/site/about/guidelines.xhtml

Discussion

To our knowledge this is the first systematic review to robustly measure the application of participatory approaches and principles to health intervention research with migrants, and specifically examine how core participatory principles of inclusivity and democracy are reflected in this application. Whilst specifically focusing on research with migrants, many of the findings, and the framework discussed are likely to provide insight to all practitioners of participatory research. The 28 studies included reported on a variety of developed health interventions, predominantly revolving around outreach and education. Our analysis shows that 18 of the 28 included studies actively involved migrants themselves, but only two studies actively involved migrants during all stages of the research process. The remaining studies either provide insufficient evidence to determine the participatory approach utilised, or were characterised by pseudo-participation, in which community groups/third-sector organisations were directly involved (proxy-participation), or migrants were only involved through being subjects in research activities (indirect-participation).

The participatory approaches taken to develop interventions in the included studies varied. The examples that represent the most participatory approach, according to our analysis, were characterised by consistent relevant inclusivity and greater democracy, which saw co-operation between researchers and migrant individuals (whom had power on par with the researchers) (Table 5). The difference between active participatory approaches, and those we characterised as pseudo-participation, appear subtle when viewed from a research-centric perspective but are stark when considering a participatory perspective. Firstly, indirect participation, in which migrants are involved in activities such as surveys or interviews designed to inform health interventions may represent a perfectly suitable means to guide development and build evidence but do little to distribute power in a participatory manner. The risk that research is framed as participatory whilst failing to develop equitable partnerships has previously been highlighted, and still appears to persist.[3,53] There is also concern that participatory research continues to be one-sided, with a continued focus on and glorification of methods on the part of researchers in studies involving migrants, at the expense of

For peer review only - http://bmjopen.bmj.com/site/about/guidelines.xhtml

participatory principles.[54] Secondly, proxy-participation, which may do more to uphold principles of participatory research, may still be at risk of not equitably involving the actual target population. Uncertainty persists around how to best involve non-academic stakeholders, and ensure those that are involved are representative of the population of interest.[55] Whilst community-groups and/or professional services involvement may at times be the only, or most readily available way to represent the population of interest (due to difficulties (perceived or otherwise) in accessing, or providing access to migrants), they cannot be assumed to be representative of them. Previous research has shown that health-service users can identify different needs to service-providers.[56] Furthermore, whilst overall understandings of involvement processes may align, service-providers may place different values on some aspects of involvement.[57] Therefore, proxy-participation could conceivably skew participatory research away from being centred on migrants' needs.

For peer review only - http://bmjopen.bmj.com/site/about/guidelines.xhtml

489 **Table 5.** Descriptive tabulation of two studies classified as displaying active participation throughout all stage of the health intervention research with
490 migrants.

		Research Stage					
		Inception	Design	Implementation	Analysis	Evaluation	Dissemination
Studies	Goodkind, Amer. 2017	Study conceived from previous relations with community groups. The present study was guided by refugees, and community service providers.	The community was involved in designing interview protocols and participant recruitment procedures.	All interpreters and interviewers were refugees; procedures had been agreed during inception and design.	Refugees were involved in analysis and were actively involved in setting the agenda for what evidence was meaningful.	Refugees and community involved in discussions to evaluate the process; indication that the decision a community intervention paradigm be adopted appears to have been adopted and championed by researchers as a result.	Refugees were involved in the dissemination and are co-authors of the research publication.
	Jacquez, Vaughn. 2019	Manifested from a previous relationship with latinos unidos por la salud to promote health and healthcare for the local latino community; co-researchers in this project were drawn from the local community.	co-researchers worked with academic partners to identify primary outcomes, and helped decide that health-worker delivered strategies were the preferred intervention option	Co-researchers recruited and worked with participants to identify strategies for stress reduction.	co-researchers and academic partners identified the primary outcomes.	Academic and community partners shared decision-making in all aspects of the research process, including evaluation.	Academic and community partners shared decision-making in all aspects of the research process, including dissemination.

For peer review only - http://bmjopen.bmj.com/site/about/guidelines.xhtml

Upholding the core principles of participatory research, in this instance, democratising research and power-sharing, is particularly pertinent to partnering with migrants. Participatory research origins are firmly rooted in increasing social justice, and the promotion of doing research with, not on or about individuals and communities, particularly those that are disadvantaged.[53] Migrant communities are often marginalised within recipient countries,[13,14] and by local health systems.[11,12] Our categorisations, and the challenges and considerations we highlight speak to the deeper underlying influence of power dynamics, which are present in all research and interactions, and can manifest at individual, interpersonal and structural levels within participatory research.[52] These dynamics should not be overlooked, regardless of the perceived benefits and potential of participatory research approaches, lest participatory research unconsciously becomes a means to reinforce and further entrench power inequities individuals such as migrant participants may experience. Not only is it inappropriate for research to perpetuate or deepen any marginalisation through failing to include migrants' voices, insights, and skills, but there are benefits to the proper utilisation of participatory approaches to the overall research process. Included studies provide evidence of the benefits to participant recruitment, implementation, and dissemination. Researchers also highlighted that the iterative nature of participatory research allows more effective tailoring of work to the needs of migrants, through learning from and embedding migrant partners knowledge and experience into research. Whilst studies we identify predominantly focus on community outreach and education within health research, participatory research could be better utilised across all disciplines. Similar methodology could be employed to better design pharmaceuticals, or on a larger scales, procedures and systems at a governance level.

Effectively partner with migrants requires specific strategies, to address the challenges identified in this review. Some of these strategies include: early participatory involvement to guide research

For peer review only - http://bmjopen.bmj.com/site/about/guidelines.xhtml

priorities, methodological approaches, and strategies to manage ongoing relations; translating and back-translating materials; giving reassurance as to the confidentiality of involvement, and respecting decisions around reporting (particularly as some partners may be undocumented migrants or have precarious legal status); utilising a variety of outreach and recruitment outlets, such as NGOs and religious groups trusted by migrants; and identifying opportunities for bidirectional benefits in the research, and capacity building to facilitate collaborative and democratic participation. Those partnering with migrants must demonstrate flexibility to negotiate potential power divides, and acknowledge and be considerate of residual mistrust that may exist among communities, even after researcher-community relationships appear well established.[58] The challenges and extra consideration highlighted by this review must not be underestimated, whilst from a research perspective, more still needs to be done to assess the impact of participatory approaches on overall research processes and output, as well as assessing whether there are distinct benefits to adopting particular participatory approaches (e.g. active, pseudo). However, if research is to become more democratic, patient-centred, and representative of the populations impacted by its work, traditional scientific approaches are likely to be inadequate, with academic researchers holding the majority of power over research.[59]

Greater adoption of consistent and transparent reporting of participatory research is needed to support the need for more critical analysis of involvement and participatory research.[17,60] Whilst guidelines have been developed,[61] they have not been widely adopted, with no material improvement in the reporting quality of published studies seen within some fields as a result of the their publication, which could be attributed to a lack of awareness of the guidelines existence.[62] Tensions exist as to whether participatory research should be conceptualised and evaluated similarly to traditional research.[60,63] However, we believe reporting can be sympathetic of the need to evidence impact of methods and processes on research. We propose the plain reporting of: who was involved in each element of the research; why these individuals were involved; how they were

For peer review only - http://bmjopen.bmj.com/site/about/guidelines.xhtml

involved; and who ultimately controlled decisions relating the research. These questions should be answered by all research involving non-academic stakeholders, at every stage of the research process. The development of guidelines to support this reporting would simultaneously allow a more complete assessment of how participatory approaches impacted overall outcomes, and greater reflection and evaluation of the participatory approaches employed;[63] Such guidelines and evaluative methods should incorporate and build upon existing monitoring tools, such as those which specifically seek to address existing challenges around power dynamics.[52] Any guidelines must also consider the distinct nature of participatory research, in that conventional evaluation is likely inappropriate, with participatory research, and consequently its' evaluation emphasising internal group evaluation which is done for combined stakeholders in an adaptive and negotiated manner.[64]

Comparing our review to existing literature there appears to be a common trend where academics and research as whole are primarily concerned with the impact or benefits of participatory approaches on research processes and outcomes, which is a valid question, but is only encompassed in our research as a secondary aim. Nevertheless, our findings as to challenges and benefits corroborate and align with existing research that participatory approaches can provide benefits particularly to the recruitment and retention of trial participants.[65-68] Challenges associated with participatory research that is previously reported and complementary to our findings often involve methodological challenges around collecting, interpreting, and disseminating research.[65,67] We found, similar to previous reviews, that the extent of engagement and involvement of non-academic stakeholders is highly varied, and that research that is expressly participatory is often limited.[17,65-68] We also find similar trends to these previous review in that there is also variability in the use of naming conventions, and application of reported approaches across all fields and topics of research.[17,66]

Our review has several limitations and caveats. Firstly, we acknowledge the taxonomy of terminology around participatory research is not standardised and terms are used inconsistently. This is an ongoing challenge within the field which has previously been evidenced in similar

For peer review only - http://bmjopen.bmj.com/site/about/guidelines.xhtml

reviews.[17] As such, the categorisations we have introduced and used in this review may be defined differently by others. There is also the possibility that additional publications that don't explicitly use the same language as we have are present in the literature, and that these articles may have been missed by our searches. Furthermore, whether the included studies categorised in our analysis exhibit greater or lesser participation is potentially immaterial to the quality of the research carried out, or the impact of the final intervention; quality criteria for participatory research have not been agreed upon or widely adopted. The amalgamation of these limitations is that formal assessments of study quality, certainty around evidence, and reporting biases, are not readily applied to this systematic review as for more homogenised approaches and methods, such as clinical trials. A further consideration relates to the reporting of benefits and challenges, in that it is often unclear which individuals or groups drove evaluations, and whether academics alone, or academics and communities in partnership decided on what was reported. We have sought to include evidence from formal evaluation where possible, but also to include evidence from the article as a whole, such as in the discussion, which could be considered reflective in nature, and potentially less rigorous on the whole. It is conceivable, given the subjective nature of some of the reported items, that differing sets of benefits and challenges would be reported dependent on the populations involved. This review represents our attempt to cast a critical lens over how the principles of participatory research are applied in practice. Our conceptual focus on migrants' involvement is therefore not intended to denigrate the efforts of third-sector organisations or professionals services, whose involvement we may have classified as proxy-participation. Fundamentally we believe the examined studies are inherently more participatory than traditional research endeavours, for having even considered and attempted to involve non-academic stakeholders. We recognise the challenges associated with participatory research, and as stated, hold no assumptions about the extent of participation and its' association with beneficial outcomes for target populations.

For peer review only

Conclusion

In conclusion, participatory approaches to developing health interventions aimed at migrants are insufficiently applied and reported. We provide evidence that the application of approaches does not fully embody core principles of participatory research, particularly relating to providing decision-making power to individuals ultimately affected by the research. Those who wish to engage in participatory research must consider the approach they take, being cognisant and open to reflecting on question of representation, democracy, and overall power dynamics, and from this critically analysing whether their approach is sufficient to achieve high-quality participation, not just high-quality research. Crucially, guidelines for reporting of participatory research methods must be introduced. This will enable all parties, from academics to communities to better assess the participatory nature of individual research projects and is an important pre-requisite to exploring the overall impact of participatory research, which currently remains inadequately understood.

For peer review only - http://bmjopen.bmj.com/site/about/guidelines.xhtml

Declarations

Ethics approval and consent to participate

This research is a systematic review of existing literature. As such, no human participants were involved.

Consent for publication

Not applicable

Availability of data and materials

All data generated or analysed during this study are included in this published article.

Competing interests

The authors declare that they have no competing interests.

Funding Statement.

This work has been funded by the Rosetrees Trust (M775) and the NIHR (NIHR300072). KR is specifically funded by the Rosetrees Trust (M775), SH, AFC, and LG are funded by the NIHR (NIHR Advanced Fellowship NIHR300072), and SH and AFC are additionally funded by the Academy of Medical Sciences (SBF005\1111). AD is funded by the MRC (MR/N013638/1). We acknowledge the support of the European Society of Clinical Microbiology and Infectious Diseases (ESCMID) Study Group for Infections in Travellers and Migrants (ESGITM).

Author Contributions

The study was conceptualised by KR, LN, SH and JSF with input on finalising the study question and protocol from SM-H and AC. Searches were carried out by KR, and screening done by KR, SM-H and AC. Data extraction and analysis was done by KR, AC and SH. Interpretation of the results and drafting of the manuscript was done primarily by KR with input from all authors (AC, SM-H, AD, LN, YC, SF, LG, JSF, SH). All authors commented upon and approved the final manuscript.

For peer review only - http://bmjopen.bmj.com/site/about/guidelines.xhtml

Acknowledgements

Not applicable

Authors' information

Not applicable

Additional Files

Additional file 1.docx – Representative search strategy and terms used in the systematic review. This additional file contains a table displaying a representative search strategy used to source articles through database searching.

Additional file 2.docx – Summary extracted data from studies included in this systematic review. This additional file contains a table displaying the summary extracted data obtained and analysed in this systematic review.

Figure Legends:

Figure 1. PRISMA flow-diagram of the study selection process

For peer review only

References

1 European Centre for Disease Prevention and Control. Guidance on community engagement for public health events caused by communicable disease threats in the EU/EEA. (ECDC; 2020, Stockholm, 2020).

2 Research., N. I. f. H. UK Standards for Public Involvement. (2019).

3 Bergold, J. & Thomes, S. Participatory Research Methods: A Methodological Approach in Motion. *Forum Qualitative Sozialforschung / Forum: Qualitative Social Research* **13**, doi:http://nbn-resolving.de/urn:nbn:de:0114-fqs1201302. (2012).

4 Position Paper 1: What is Participatory Health Research? , (International Collaboration for Participatory Health Research, Berlin, 2013).

5 Springett, J., Katkey, K., Kongats, K., Zulla, R. & Wilkins, E. Conceptualizing Quality in Participatory Health Research: A Phenomenographic Inquiry. *Forum Qualitative Sozialforschung / Forum: Qualitative Social Research* **17**, doi:http://nbn-resolving.de/urn:nbn:de:0114-fqs1602274. (2016).

6 Crawshaw, A. F. *et al.* What must be done to tackle vaccine hesitancy and barriers to COVID-19 vaccination in migrants? *Journal of Travel Medicine*, doi:10.1093/jtm/taab048 (2021).

7 Israel, B. A., Schulz, A. J., Parker, E. A. & Becker, A. B. Community-based participatory research: policy recommendations for promoting a partnership approach in health research. *Education for health (Abingdon, England)* **14**, doi:10.1080/13576280110051055 (2001).

8 Koshy, E., Koshy, V. & Waterman, H. *Action Research in Healthcare*. Vol. 2021 (SAGE Publications Ltd, 2011).

9 Health Research Authority. *What is public involvement in research?*, 2021).

10 Cargo, M. & Mercer, S. The Value and Challenges of Participatory Research: Strengthening its Practice. *Annual Review of Public Health* **29**, 325-350 (2008).

11 Legido-Quigley, H. *et al.* Healthcare is not universal if undocumented migrants are excluded. doi:10.1136/bmj.l4160 (2019).

12 O'Donnel, c. Health Care Access for Migrants in Europe - Oxford Research Encyclopedia of Global Public Health. *Global Public Health*, doi:10.1093/acrefore/9780190632366.013.6 (2018).

13 Thorbjørnsrud, K. & Figenschou, T. U. Do Marginalized Sources Matter? *http://dx.doi.org/10.1080/1461670X.2014.987549*, doi:10.1080/1461670X.2014.987549 (2014).

14 Migrants perspectives on Brexit and UK immigration policies. (Migrants Rights Network, 2017).

15 Health promotion for improved refugee and migrant health: Technical guidance. (World Health Organization, Geneva, 2017).

16 O'Mara-Eves, A. *et al.* The effectiveness of community engagement in public health interventions for disadvantaged groups: a meta-analysis. *BMC Public Health* **15**, 1-23, doi:doi:10.1186/s12889-015-1352-y (2015).

17 Vaughn, L. M. *et al.* Partnering with insiders: A review of peer models across community-engaged research, education and social care. *Health & social care in the community* **26**, doi:10.1111/hsc.12562 (2018).

For peer review only - http://bmjopen.bmj.com/site/about/guidelines.xhtml

18 Cook, T. Where Participatory Approaches Meet Pragmatism in Funded (Health) Research: The Challenge of Finding Meaningful Spaces. *Forum Qualitative Sozialforschung / Forum: Qualitative Social Research* **13**, doi:http://dx.doi.org/10.17169/fqs-13.1.1783 (2012).

19 Ouzzani, M., Hammady, H., Fedorowicz, Z. & Elmagarmid, A. Rayyan—a web and mobile app for systematic reviews. *Systematic Reviews* **5**, 1-10, doi:doi:10.1186/s13643-016-0384-4 (2016).

20 *Core Values, Ethics, Spectrum - The 3 Pillars of Public Participation*, <https://www.iap2.org/page/pillars> (2020).

21 Henderson, A. & Slater, J. Growing Roots: A Newcomer Nutrition Program Designed Using Action Research Methods. *Ecology of Food and Nutrition* **58**, 430-455, doi:10.1080/03670244.2019.1636792. (2019).

22 Afifi, R. A., Makhoul, J., El Hajj, T. & Nakkash, R. T. Developing a logic model for youth mental health: participatory research with a refugee community in Beirut. *Health Policy Plan* **26**, 508-517, doi:10.1093/heapol/czr001 (2011).

23 Nilvarangkul, K., McCann, T. V., Rungreangkulkij, S. & Wongprom, J. Enhancing a health-related quality-of-life model for Laotian migrant workers in Thailand. *Qual Health Res* **21**, 312-323, doi:10.1177/1049732310376635 (2011).

24 Evans, C. *et al.* Mobile phone messaging to promote uptake of HIV testing among migrant African communities in the UK:. *https://doi.org/10.1177/0017896918785928*, doi:10.1177_0017896918785928 (2018).

25 Aitaoto, N., Braun, K. L., Estrella, J., Epeluk, A. & Tsark, J. Design and results of a culturally tailored cancer outreach project by and for Micronesian women. *Prev Chronic Dis* **9**, E82, doi:10.5888/pcd9.100262 (2012).

26 Barbee, L. *et al.* Assessing the acceptability of self-sampling for HPV among Haitian immigrant women: CBPR in action. *Cancer Causes Control* **21**, 421-431, doi:10.1007/s10552-009-9474-0 (2010).

27 Chesla, C. A. *et al.* Testing the efficacy of culturally adapted coping skills training for Chinese American immigrants with type 2 diabetes using community-based participatory research. *Res Nurs Health* **36**, 359-372, doi:10.1002/nur.21543 (2013).

28 Forst, L. *et al.* More than training: Community-based participatory research to reduce injuries among hispanic construction workers. *Am J Ind Med* **56**, 827-837, doi:10.1002/ajim.22187 (2013).

29 Goodkind, J. R. *et al.* Challenges and Innovations in a Community-Based Participatory Randomized Controlled Trial. *Health Education & Behavior* **44**, 123-130, doi:10.1177/1090198116639243 (2017).

30 Jacquez, F., Vaughn, L. M. & Suarez-Cano, G. Implementation of a Stress Intervention with Latino Immigrants in a Non-traditional Migration City. *Journal of Immigrant and Minority Health* **21**, 372-382, doi:doi:10.1007/s10903-018-0732-7 (2018).

31 Kaiser, L. *et al.* Adaptation of a culturally relevant nutrition and physical activity program for low-income, Mexican-origin parents with young children. *Prev Chronic Dis* **12**, E72, doi:10.5888/pcd12.140591 (2015).

32 Kandula, N. R. *et al.* An Exercise Intervention for South Asian Mothers with Risk Factors for Diabetes. *Transl J Am Coll Sports Med* **1**, 52-59 (2016).

33 Karasz, A. *et al.* ASHA: Using Participatory Methods to Develop an Asset-building Mental Health

Intervention for Bangladeshi Immigrant Women. *Progress in Community Health Partnerships: Research, Education, and Action* **9**, 501-512, doi:https://doi.org/10.1353/cpr.2015.0080 (2015).

34 Kim, S., Koniak-Griffin, D., Flaskerud, J. H. & Guarnero, P. A. The impact of lay health advisors on cardiovascular health promotion: using a community-based participatory approach. *J Cardiovasc Nurs* **19**, 192-199, doi:10.1097/00005082-200405000-00008 (2004).

For peer review only - http://bmjopen.bmj.com/site/about/guidelines.xhtml

35 Lam, T. K. *et al.* Encouraging Vietnamese-American Women to Obtain Pap Tests Through Lay Health Worker Outreach and Media Education. *J Gen Intern Med* **18**, 516-524, doi:10.1046/j.1525-1497.2003.21043.x (2003).

36 Li, M., Yeh, Y.-L., Sun, H., Chang, B. & Chen, L.-s. Community-Based Participatory Research: a Family Health History-Based Colorectal Cancer Prevention Program Among Chinese Americans. *Journal of Cancer Education : the Official Journal of the American Association for Cancer Education*, doi:10.1007/s13187-019-1486-1 (2019).

37 Pinsker, E. *et al.* The Development of Culturally Appropriate Tobacco Prevention Videos Targeted

Toward Somali Youth. *Progress in Community Health Partnerships: Research, Education, and Action* **11**, doi:https://doi.org/10.1353/cpr.2017.0017 (2017).

38 Quandt, S. A. *et al.* Evaluating the effectiveness of a lay health promoter-led, community-based participatory pesticide safety intervention with farmworker families. *Health Promot Pract* **14**, 425-432, doi:10.1177/1524839912459652 (2013).

39 Solorio, R., Norton-Shelpuk, P., Forehand, M., Martinez, M. & Aguirre, J. HIV Prevention Messages Targeting Young Latino Immigrant MSM. *AIDS Res Treat* **2014**, 353092, doi:10.1155/2014/353092 (2014).

40 Song, H. J. *et al.* Translating current dietary guidelines into a culturally tailored nutrition education program for Korean American immigrants with type 2 diabetes. *Diabetes Educ* **36**, 752-761, doi:10.1177/0145721710376328 (2010).

41 Suarez-Balcazar, Y. *et al.* Community-based participatory research to promote healthy lifestyles among Latino immigrant families with youth with disabilities. *https://doi.org/10.1080/11038128.2018.1502348*, doi:1502348 (2018).

42 Vaughn, L., Jacquez, F. & Suarez-Cano, G. Developing and Implementing a Stress and Coping Intervention in Partnership With Latino Immigrant Coresearchers. *Translational Issues in Psychological Science* **5**, 62-77, doi:10.1037/tps0000172 (2019).

43 Wieland, M. L. *et al.* Sustainability of a Tuberculosis Screening Program at an Adult Education Center Through Community-Based Participatory Research. *J Public Health Manag Pract* **25**, 602-605, doi:10.1097/phh.0000000000000851 (2019).

44 Wieland, M. L. *et al.* Pilot Feasibility Study of A Digital Storytelling Intervention for Immigrant and Refugee Adults with Diabetes. *Diabetes Educ* **43**, 349-359, doi:10.1177/0145721717713317 (2017).

45 Wieland, M. L. *et al.* Physical activity and nutrition among immigrant and refugee women: a community-based participatory research approach. *Womens Health Issues* **22**, 225-232, doi:10.1016/j.whi.2011.10.002. (2012).

46 Williams, Q., Jr., Ochsner, M., Marshall, E., Kimmel, L. & Martino, C. The impact of a peer-led participatory health and safety training program for Latino day laborers in construction. *J Safety Res* **41**, 253-261, doi:10.1016/j.jsr.2010.02.009 (2010).

47 Baird, M. B. *et al.* Creating a Bridge of Understanding between Two Worlds: Community-Based Collaborative-Action Research with Sudanese Refugee Women. *Public Health Nurs* **32**, 388-396, doi:10.1111/phn.12172 (2015).

48 Grigg-Saito, D., Och, S., Liang, S., Toof, R. & Silka, L. Building on the strengths of a Cambodian refugee community through community-based outreach. *Health Promot Pract* **9**, 415-425, doi:10.1177/1524839906292176 (2008).

49 Goodkind, J. R. *et al.* Challenges and Innovations in a Community-Based Participatory Randomized Controlled Trial. *https://doi.org/10.1177/1090198116639243*, doi:10.1177_1090198116639243 (2016).

50 Coffman, M. J. *et al.* Using CBPR to Decrease Health Disparities in a Suburban Latino Neighborhood. *Hisp Health Care Int* **15**, 121-129, doi:10.1177/1540415317727569 (2017).

For peer review only

51 Documet, P. I. *et al.* A Male Promotores Network for Latinos: Process Evaluation From a Community-Based Participatory Project. *Health Promot Pract* **17**, 332-342, doi:10.1177/1524839915609059 (2016).

52 Roura, M. The Social Ecology of Power in Participatory Health Research. *https://doi.org/10.1177/1049732320979187*, doi:10.1177_1049732320979187 (2020).

53 Macaulay, A. C. Participatory research: What is the history? Has the purpose changed? *Family Practice* **34**, 256-258, doi:https://doi.org/10.1093/fampra/cmw117 (2017).

54 Ozkul, D. Participatory Research: Still a One-Sided Research Agenda? *Migration Letters* **17**, doi:https://journals.tplondon.com/ml/article/view/804 (2020).

55 Lander, J., Langhof, H. & Dierks, M.-L. Involving patients and the public in medical and health care research studies: An exploratory survey on participant recruiting and representativeness from the perspective of study authors. *PLoS One* **14**, doi:10.1371/journal.pone.0204187 (2019).

56 Edwards, L. *et al.* What matters most to patients about primary healthcare: mixed-methods patient priority setting exercises within the PREFeR (PRioritiEs For Research) project. doi:10.1136/bmjopen-2018-025954 (2019).

57 Rise, M. B. *et al.* Same description, different values. How service users and providers define patient and public involvement in health care. *Health Expect.* **16**, 266-276, doi:10.1111/j.1369-7625.2011.00713.x (2013).

58 Ahmed, A., Vandrevala, T., Hendy, J., Kelly, C. & Ala, A. An examination of how to engage migrants in the research process: building trust through an ‘insider’ perspective. *https://doi.org/10.1080/13557858.2019.1685651*, doi:CETH-2019-0030.R2 (2019).

59 Green, G. Power to the people: To what extent has public involvement in applied health research achieved this? *Research Involvement and Engagement* **2**, 1-13, doi:doi:10.1186/s40900-016-0042-y (2016).

60 Russel, J., Fudge, N. & Greenhalgh, T. The impact of public involvement in health research: what are we measuring? Why are we measuring it? Should we stop measuring it? *Research Involvement and Engagement* **6**, doi:https://doi.org/10.1186/s40900-020-00239-w (2020).

61 Smith, L., Rosenzweig, L. & Schmidt, M. Best Practices in the Reporting of Participatory Action Research: Embracing Both the Forest and the Trees 1Ψ7:. *http://dx.doi.org/10.1177/0011000010376416*, doi:10.1177_0011000010376416 (2010).

62 Kato, D. *et al.* Reporting Guidelines for Community-Based Participatory Research Did Not Improve the Reporting Quality of Published Studies: A Systematic Review of Studies on Smoking Cessation. *Int J Environ Res Public Health* **17**, doi:10.3390/ijerph17113898 (2020).

63 Staley, K. & Barron, D. Learning as an outcome of involvement in research: what are the implications for practice, reporting and evaluation? *Research Involvement and Engagement* **5**, doi:https://doi.org/10.1186/s40900-019-0147-1 (2019).

64 Campilan, D. in *Forum on Evaluation of International Cooperation Projects: Centering on Development of Human Resources in the Field of Agriculture.*

65 De las Nueces, D., Hacker, K., DiGirolamo, A. & Hicks, L. S. A systematic review of community-based participatory research to enhance clinical trials in racial and ethnic minority groups. *Health services research* **47**, doi:10.1111/j.1475-6773.2012.01386.x (2012).

66 Vaughn, L. M., Jacquez, F., Lindquist-Grantz, R., Parsons, A. & Melink, K. Immigrants as Research Partners: A Review of Immigrants in Community-Based Participatory Research (CBPR). *Journal of Immigrant and Minority Health* **19**, 1457-1468, doi:doi:10.1007/s10903-016-0474-3 (2016).

67 Dadich, A., Moore, L. & Eapen. What does it mean to conduct participatory research with Indigenous peoples? A lexical review. *BMC public health* **19**, doi:10.1186/s12889-019-7494-6 (2019).

68 Wieland, M. L., Njeru, J. W., Alahdab, F., Doubeni, C. A. & Sia, I. G. Community-Engaged Approaches for Minority Recruitment Into Clinical Research: A Scoping Review of the

For peer review only

Literature. *Mayo Clinical Proceedings* **93**, 733-743, doi:https://doi.org/10.1016/j.mayocp.2020.03.028 (2021).

For peer review only

For peer review only - http://bmjopen.bmj.com/site/about/guidelines.xhtml

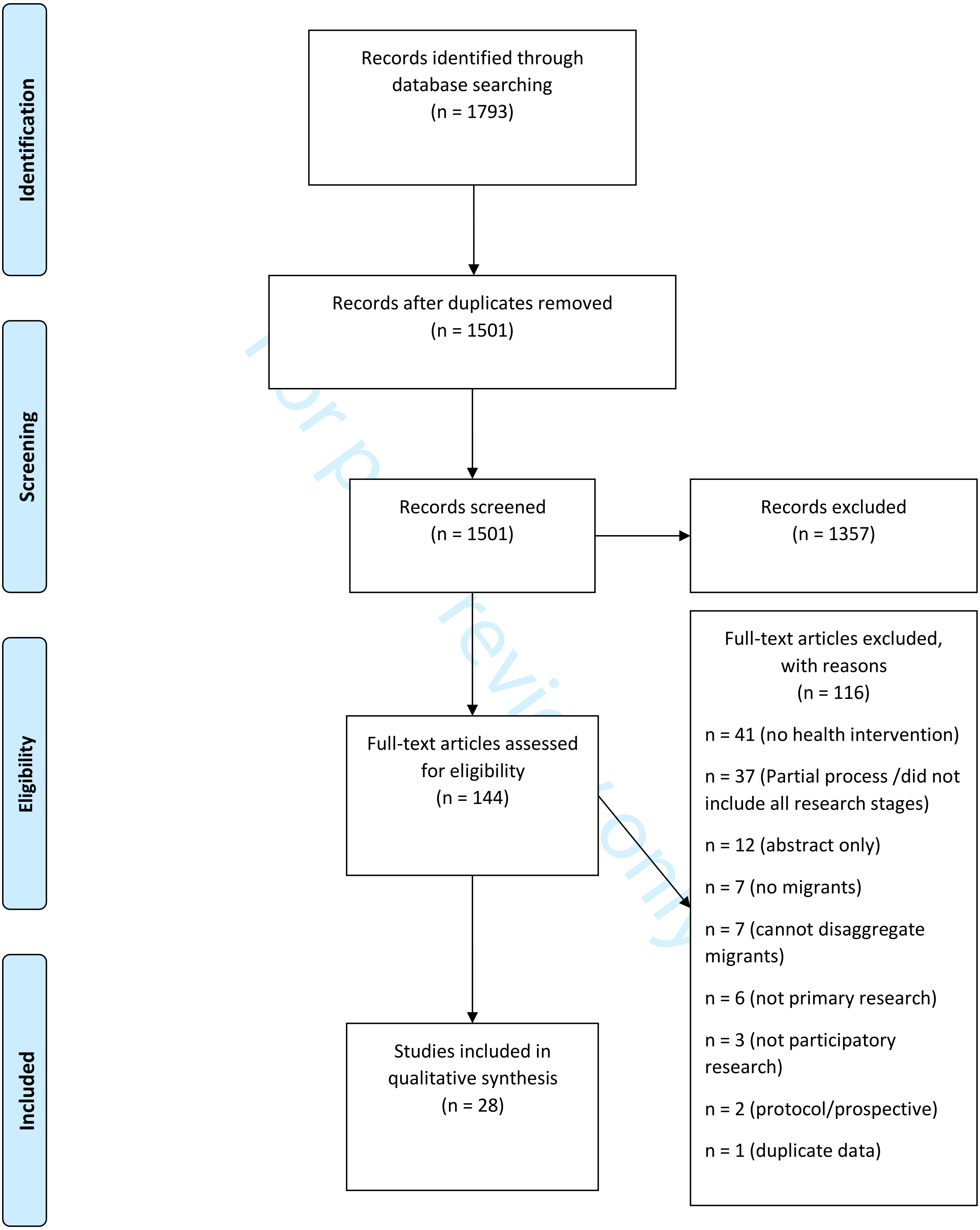

Figure 1. PRISMA flow-diagram of the study selection process

Additional File 1. Full reproduction of searches used within the individual databases

MEDLINE Database Search Strategy (1946 – November 2020):

1. "Transients and Migrants"/ or Migrant*.mp.
2. Migrat*.mp. [mp=title, abstract, heading word, drug trade name, original title, device manufacturer, drug manufacturer, device trade name, keyword, floating subheading word, candidate term word]
3. Refugee*.mp. or Refugees/
4. Asylum-seeker*.mp.
5. Foreign*.mp.
6. Foreign-born.mp.
7. Non-native*.mp.
8. "Emigrants and Immigrants"/ or Immigra*.mp.
9. Emigra*.mp. or "Emigration and Immigration"/
10. Oversea*.mp.
11. Foreign student*.mp.
12. International Student*.mp.
13. 1 or 2 or 3 or 4 or 5 or 6 or 7 or 8 or 9 or 10 or 11 or 12
14. Co-design*.mp.
15. Co-prod*.mp. [mp=title, abstract, heading word, drug trade name, original title, device manufacturer, drug manufacturer, device trade name, keyword, floating subheading word, candidate term word]
16. Co-creat*.mp. [mp=title, abstract, heading word, drug trade name, original title, device manufacturer, drug manufacturer, device trade name, keyword, floating subheading word, candidate term word]
17. Collab* design*.mp. [mp=title, abstract, heading word, drug trade name, original title, device manufacturer, drug manufacturer, device trade name, keyword, floating subheading word, candidate term word]
18. Collab* prod*.mp. [mp=title, abstract, heading word, drug trade name, original title, device manufacturer, drug manufacturer, device trade name, keyword, floating subheading word, candidate term word]
19. Collab* creat*.mp. [mp=title, abstract, heading word, drug trade name, original title, device manufacturer, drug manufacturer, device trade name, keyword, floating subheading word, candidate term word]
20. Community design*.mp.
21. Community prod*.mp.
22. Community creat*.mp.
23. Community-based participatory research.mp. [mp=title, abstract, heading word, drug trade name, original title, device manufacturer, drug manufacturer, device trade name, keyword, floating subheading word, candidate term word]
24. Participatory design*.mp.
25. Participatory action research.mp. or Community-Based Participatory Research/
26. (Participatory adj4 Research).mp. [mp=title, abstract, heading word, drug trade name, original title, device manufacturer, drug manufacturer, device trade name, keyword, floating subheading word, candidate term word]
27. Action Research.mp.
28. 14 or 15 or 16 or 17 or 18 or 19 or 20 or 21 or 22 or 23 or 24 or 25 or 26 or 27
29. 13 and 28

Embase Database Search Strategy (1974 – November 2020):

1. migrant/ or migrant worker/ or Migrant*.mp.
2. migration/ or Migrat*.mp.
3. refugee/ or Refugee*.mp.
4. asylum seeker/ or Asylum seeker*.mp.
5. Foreign*.mp.
6. Foreign Born.mp.
7. Non-native*.mp.
8. immigration/ or immigrant/ or Immigra*.mp.
9. emigrant/ or emigration/ or Emigrants/ or Emigra*.mp.
10. Oversea*.mp.

11. Foreign Student*.mp. or foreign student/
12. International Student*.mp.
13. 1 or 2 or 3 or 4 or 5 or 6 or 7 or 8 or 9 or 10 or 11 or 12
14. Co-design*.mp.
15. Co-prod*.mp.
16. Co-creat*.mp.
17. Collab* design*.mp.
18. Collab* prod*.mp.
19. Collab* creat*.mp.
20. Community design*.mp.
21. Community prod*.mp.
22. Community creat*.mp.
23. Community-based participatory research.mp. [mp=title, abstract, heading word, drug trade name, original title, device manufacturer, drug manufacturer, device trade name, keyword, floating subheading word, candidate term word]
24. Action research.mp. or action research/
25. Participatory research.mp. or participatory research/
26. Participatory action research.mp.
27. (Participatory adj4 Research).mp. [mp=title, abstract, heading word, drug trade name, original title, device manufacturer, drug manufacturer, device trade name, keyword, floating subheading word, candidate term word]
28. 14 or 15 or 16 or 17 or 18 or 19 or 20 or 21 or 22 or 23 or 24 or 25 or 26 or 27
29. 13 and 28

Global Health Database Search Strategy (1920 – November 2020):

1. Migrant*.mp.
2. Migrat*.mp. [mp=title, abstract, heading word, drug trade name, original title, device manufacturer, drug manufacturer, device trade name, keyword, floating subheading word, candidate term word]
3. Refugee*.mp. or refugees/
4. Asylum-seeker*.mp.
5. Foreign*.mp.
6. Foreign-born.mp.
7. Non-native*.mp.
8. Immigra*.mp.
9. Emigra*.mp. [mp=title, abstract, heading word, drug trade name, original title, device manufacturer, drug manufacturer, device trade name, keyword, floating subheading word, candidate term word]
10. Oversea*.mp.
11. Foreign Student*.mp. or foreign students.sh.
12. International Student*.mp.
13. 1 or 2 or 3 or 4 or 5 or 6 or 7 or 8 or 9 or 10 or 11 or 12
14. Co-design*.mp.
15. Co-prod*.mp. [mp=title, abstract, heading word, drug trade name, original title, device manufacturer, drug manufacturer, device trade name, keyword, floating subheading word, candidate term word]
16. Co-creat*.mp. [mp=title, abstract, heading word, drug trade name, original title, device manufacturer, drug manufacturer, device trade name, keyword, floating subheading word, candidate term word]
17. Collab* design*.mp. [mp=title, abstract, heading word, drug trade name, original title, device manufacturer, drug manufacturer, device trade name, keyword, floating subheading word, candidate term word]
18. Collab* prod*.mp. [mp=title, abstract, heading word, drug trade name, original title, device manufacturer, drug manufacturer, device trade name, keyword, floating subheading word, candidate term word]
19. Collab* creat*.mp. [mp=title, abstract, heading word, drug trade name, original title, device manufacturer, drug manufacturer, device trade name, keyword, floating subheading word, candidate term word]
20. Community design*.mp.
21. Community prod*.mp. [mp=title, abstract, heading word, drug trade name, original title, device manufacturer, drug manufacturer, device trade name, keyword, floating subheading word, candidate term word]
22. Community creat*.mp.

23.	Community-based participatory research.mp. [mp=title, abstract, heading word, drug trade name, original title, device manufacturer, drug manufacturer, device trade name, keyword, floating subheading word, candidate term word]
24.	Participatory design*.mp.
25.	Participatory action research.mp.
26.	(Participatory adj4 Research).mp. [mp=title, abstract, heading word, drug trade name, original title, device manufacturer, drug manufacturer, device trade name, keyword, floating subheading word, candidate term word]
27.	Action research.mp.
28.	14 or 15 or 16 or 17 or 18 or 19 or 20 or 21 or 22 or 23 or 24 or 25 or 26 or 27
29.	13 and 28

PsychINFO Database Search Strategy (1967 – November 2020):

1.	Migrant*.mp.
2.	Migrat*.mp.
3.	exp REFUGEES/ or Refugee*.mp.
4.	exp Asylum Seeking/ or Asylum-seeker*.mp.
5.	Foreign*.mp.
6.	Foreign-born.mp.
7.	Non-native*.mp.
8.	exp Immigration/ or Immigra*.mp.
9.	Emigra*.mp. [mp=title, abstract, heading word, drug trade name, original title, device manufacturer, drug manufacturer, device trade name, keyword, floating subheading word, candidate term word]
10.	Oversea*.mp. [mp=title, abstract, heading word, drug trade name, original title, device manufacturer, drug manufacturer, device trade name, keyword, floating subheading word, candidate term word]
11.	Foreign Student*.mp.
12.	exp International Students/ or International Student*.mp.
13.	1 or 2 or 3 or 4 or 5 or 6 or 7 or 8 or 9 or 10 or 11 or 12
14.	Co-design*.mp.
15.	Co-prod*.mp. [mp=title, abstract, heading word, drug trade name, original title, device manufacturer, drug manufacturer, device trade name, keyword, floating subheading word, candidate term word]
16.	Co-creat*.mp. [mp=title, abstract, heading word, drug trade name, original title, device manufacturer, drug manufacturer, device trade name, keyword, floating subheading word, candidate term word]
17.	Collab* design*.mp.
18.	Collab* prod*.mp. [mp=title, abstract, heading word, drug trade name, original title, device manufacturer, drug manufacturer, device trade name, keyword, floating subheading word, candidate term word]
19.	Collab* creat*.mp. [mp=title, abstract, heading word, drug trade name, original title, device manufacturer, drug manufacturer, device trade name, keyword, floating subheading word, candidate term word]
20.	Community design*.mp.
21.	Community prod*.mp.
22.	Community creat*.mp. [mp=title, abstract, heading word, drug trade name, original title, device manufacturer, drug manufacturer, device trade name, keyword, floating subheading word, candidate term word]
23.	Community-based participatory research.mp. [mp=title, abstract, heading word, drug trade name, original title, device manufacturer, drug manufacturer, device trade name, keyword, floating subheading word, candidate term word]
24.	Participatory design*.mp.
25.	Participatory research.mp.
26.	(Participatory adj4 Research).mp. [mp=title, abstract, heading word, drug trade name, original title, device manufacturer, drug manufacturer, device trade name, keyword, floating subheading word, candidate term word]
27.	Participatory action research.mp.
28.	Action research.mp. or exp Action Research/
29.	14 or 15 or 16 or 17 or 18 or 19 or 20 or 21 or 22 or 23 or 24 or 25 or 26 or 27 or 28
30.	13 and 29

Additional File 2. Summary of extracted data relating to involvement and engagement of non-academic stakeholders, and their influence over given research stages.

1. Afifi, Makhoul et al. 2011

Aim & Target population: *Development of a mental health promotion intervention for Palestinian refugee youths.*

Inception: *Based on a previous survey of the target population which had been designed with community input*
Design: *Interview and survey of target population; formation of a community youth council with youth council representatives which agreed internal rules and implemented democratic voting re: design decisions*
Implementation: *No explicit evidence of non-academic stakeholder involvement, engagement, or influence.*
Analysis: *No explicit evidence of non-academic stakeholder involvement, engagement, or influence.*
Evaluation: *No explicit evidence of non-academic stakeholder involvement, engagement, or influence.*
Dissemination: *No explicit evidence of non-academic stakeholder involvement, engagement, or influence.*

2. Aitaoto, Braun et al. 2012

Aim & Target population: *Implementation of a cancer outreach programme for Micronesian women*

Inception: *Community group representing the target population approached researchers to highlight this topic*
Design: *Focus groups with the target population; champions from the target population were involved in literature review and aided in designing the outreach programme*
Implementation: *Target population delivered the programme as lay-educators, a decision clearly defined in the design stage by working with the champions*
Analysis: *No explicit evidence of non-academic stakeholder involvement, engagement, or influence.*
Evaluation: *No explicit evidence of non-academic stakeholder involvement, engagement, or influence; reported that the research did not ask the target population about perceptions of the intervention*
Dissemination: *No explicit evidence of non-academic stakeholder involvement, engagement, or influence.*

3. Baird, Domian et al. 2015

Aim & Target population: *To address the health challenges associated with relocation among Sudanese refugees*

Inception: *Collaborative inception between Sudanese community and researchers manifesting from previous research shared to community members, who then formed a committee which worked with the researchers; Whilst topics for intervention were collaboratively developed between researchers and the target population*
Design: *research team developed educational seminars on intervention topics.*
Implementation: *Educational seminars were delivered by the research team; target population were involved in advertising and recruiting other participants.*
Analysis: *No explicit evidence of non-academic stakeholder involvement, engagement, or influence.*
Evaluation: *Focus group discussion to evaluate the process, discuss overall findings, and plan future relationships.*

For peer review only

	Dissemination: *No explicit evidence of non-academic stakeholder involvement, engagement, or influence.*
4.	**Barbee, Kobetz et al. 2010**<br><br>***Aim & Target population:*** *To assess the acceptability of HPV self-sampling with community health workers, to detect cervical cancer among Haitian immigrant women.*<br><br>***Inception:*** *Conceived by a campus-community partnership; the partnership is formed of community leaders and academics; community leaders are largely responsible for identifying research needs*<br>***Design:*** *Community partners responsible for defining research focus, recruitment strategies, and data collection methods; academic partners write grants applications and obtain institutional approvals*<br>***Implementation:*** *Community and academic partners contribute collectively to study implementation*<br>***Analysis:*** *No explicit evidence of non-academic stakeholder involvement, engagement, or influence.*<br>***Evaluation:*** *No explicit evidence of non-academic stakeholder involvement, engagement, or influence.*<br>***Dissemination:*** *Community and academic partners contribute collectively to study dissemination*
5.	**Chesla, Chun. 2013**<br><br>***Aim & Target population:*** *Cultural adaptation of coping skills training for Chinese-American immigrants with type 2 diabetes.*<br><br>***Inception:*** *No explicit evidence of non-academic stakeholder involvement, or influence; there is mention of a CBPR work group, and a community advisory board, but no indication these groups were involved in the inception.*<br>***Design:*** *A CBPR work group composed of 10 paid staff from community agencies and academic institutions. The work group culturally adapted protocols based on clinical guidelines, clinical knowledge, and previous studies.; A community advisory board met semi-annually and provided oversight on all aspect of the CBPR activities.*<br>***Implementation:*** *Work group members who were bilingual with degree level qualification in social work implemented the intervention.*<br>***Analysis:*** *No explicit evidence of non-academic stakeholder involvement, engagement, or influence.*<br>***Evaluation:*** *Evaluated through research/survey with participants.*<br>***Dissemination:*** *No explicit evidence of non-academic stakeholder involvement, engagement, or influence.*
6.	**Evans, Suggs et al. 2019**<br><br>***Aim & Target population:*** *Promoting HIV testing uptake amongst African migrant communities*<br><br>***Inception:*** *No explicit evidence of non-academic stakeholder involvement, engagement, or influence.*<br>***Design:*** *The design was guided by previous formative research conducted by the researchers*<br>***Implementation:*** *Participants were invited by community researchers that represented diverse African communities and genders.*<br>***Analysis:*** *No explicit evidence of non-academic stakeholder involvement, engagement, or influence.*<br>***Evaluation:*** *The target population provided feedback through surveys*<br>***Dissemination:*** *No explicit evidence of non-academic stakeholder involvement, engagement, or influence.*

For peer review only

**7. Forst, Ahonen et al. 2013**<br><br>***Aim & Target population:*** Increase awareness of workplace hazards and self-efficacy among foreign-born Hispanic construction workers; and expand worker centre agenda to include occupational health & safety.<br><br>***Inception:*** *No explicit evidence of non-academic stakeholder involvement, engagement, or influence.*<br>***Design:*** *Worker centres (WC) and academics partnered to set joint goals and specific responsibilities: WC would recruit peer-educators and workers to the training, partner with implementation and evaluation; university partners assumed responsibility for subject protection, evaluation, design and implementation.*<br>***Implementation:*** *Worker centre staff were responsible for recruiting peer-educators to deliver the curriculum; university partners assumed responsibility for implementation*<br>***Analysis:*** *No explicit evidence of non-academic stakeholder involvement, engagement, or influence.*<br>***Evaluation:*** *Iterative evaluation of the intervention with peer-educators in a democratic manner; peer-educators were able to influence study design*<br>***Dissemination:*** *No explicit evidence of non-academic stakeholder involvement, engagement, or influence.*
**8. Goodkind, Amer et al. 2017**<br><br>***Aim & Target population:*** **to address social determinants of health, improve links to mental health services, and improve retention in trauma-focused treatment for Afghan, Great lakes region african, and iraqi Refugee adults**<br><br>***Inception:*** *Inception came from previous work with other community groups; present study guided by refugees, formers students and community service providers*<br>***Design:*** *Community involved in designing the interview protocols, and participant recruitment*<br>*Implementation: target population involved in implementation; all interpreters and interviewers were refugees*<br>***Analysis:*** *target population involved in analysis; active involvement in asking what evidence is meaningful*<br>***Evaluation:*** *CAC were involved in conversation concerning the use of CAC; have voice opinions that a community intervention paradigm should be used which the researchers appear to have heeded.*<br>***Dissemination****: target population involved in dissemination; 2nd and 8th author are refugees*
**9. Grigg-Saito, Och et al.**<br><br>***Aim & Target population:*** *Outreach to reduce health disparities in cardiovascular disease and diabetes among Cambodian refugees*<br><br>***Inception:*** *Coalition formed prior to this intervention to address needs of Cambodian community - resulted in the funding bid for this work; the coalition involved community leaders and elders*<br>***Design:*** *Collaboration amongst the coalition to develop a community action plan. Target population took part in focus groups; community forum involving target population (engagement event) took place*<br>***Implementation:*** *Cambodian community health programme Staff health educators go door-to-door, staff also encouraged participation from elders; Staff conducted business outreach and educational groups/workshops; A peer-support group was established; elders council (advisory group) established at the behest of elder program participants*<br>***Analysis:*** *No explicit evidence of non-academic stakeholder involvement, engagement, or influence.*<br>***Evaluation:*** *Data was reported back to staff and steering committee periodically, everyone was able to contribute to lessons learned, draw conclusions and make suggestions for improvements based on the evaluation.*

For peer review only

***Dissemination**: No explicit evidence of non-academic stakeholder involvement, engagement, or influence.*

10. Henderson, Slater

Aim & Target population: Nutrition information for newly arrived immigrants

***Inception:** based on previous community-based participatory research*
***Design:** Community consultation*
Implementation: *No explicit evidence of non-academic stakeholder involvement, engagement, or influence.*
Analysis: *No explicit evidence of non-academic stakeholder involvement, engagement, or influence.*
***Evaluation:** community consultation on evaluation*
***Dissemination**: No explicit evidence of non-academic stakeholder involvement, engagement, or influence.*

11. Jacquez, Vaughn et al. 2019

***Aim & Target population:** Implementation of a stress reduction intervention for latino immigrants.*

***Inception:** Manifested from a previous relationship with latinos unidos por la salud to promote health and healthcare for the local latino community; community members applied and were selected as co-researchers*
***Design**: Community group co-researchers worked with academic partners to identify primary outcomes, and that health-worker delivered strategies were the preferred intervention option.*
***Implementation:** Co-researchers recruited and worked with participants to identify strategies with participants.*
***Analysis:** co-researchers and academic partners identified the primary outcomes*
***Evaluation:** Academic and community partners shared decision-making in all aspects of the research process, including evaluation.*
***Dissemination**: Academic and community partners shared decision-making in all aspects of the research process, including dissemination.*

12. Kaiser, Martinez et al. 2015

***Aim & Target population:** Adaptation of a culturally relevant nutrition and exercise program for Mexican immigrant parents with young children*

***Inception:** Researchers conceived through reviewing literature and involving the target population in focus groups to assess their interpretation and prioritisation of key childhood obesity messages.*
***Design:** Researchers performed a literature search and involved the target population in focus groups; the researchers presented the plans for a nutrition program to a local advisory committee whom convened quarterly and gave advice on how to deal with specific issues*
***Implementation:** A local educator with experience of family counselling was hired to deliver the intervention*
Analysis: *No explicit evidence of non-academic stakeholder involvement, engagement, or influence.*
***Evaluation:** pilot testing with a subsection of families who were invited to give feedback; educator and specialist would evaluate intervention events after each one for changes; participants were invited to focus groups to report back on the intervention.*
***Dissemination**: No explicit evidence of non-academic stakeholder involvement, engagement, or influence.*

13. Kandula, Dave et al. 2016

For peer review only

Aim & Target population: *Exercise intervention for south-asian mother with risk factors for diabetes*

Inception: *Local community group and researchers collaboratively chose the focus of the research*
Design: *Community partner and academics adapted and applied evidence-based behaviour change principles in partnership; a memorandum of understanding was agreed by the partners: The community group was involved in design, implementation, recruitment and evaluation.*
Implementation: *Community partner was responsible for recruitment and outreach*
Analysis: *No explicit evidence of non-academic stakeholder involvement, engagement, or influence.*
Evaluation: *The community partner was involved in evaluation; participants were involved in the evaluation of the intervention through qualitative interviews.*
Dissemination*: No explicit evidence of non-academic stakeholder involvement, engagement, or influence.*

14. Karasz, Raghavan et al. 2015

Aim & Target population: *Development of an asset-building mental health intervention for Bangladeshi immigrant women*

Inception: *Outgrowth from previous partnerships; the development involved local physicians, a health advocate and researchers.*
Design: *The target population were included as part of the intervention design group*
Implementation: *No explicit evidence of non-academic stakeholder involvement, engagement, or influence.*
Analysis: *No explicit evidence of non-academic stakeholder involvement, engagement, or influence.*
Evaluation: *No explicit evidence of non-academic stakeholder involvement, engagement, or influence; target population were involved in the evaluation through research methods.*
Dissemination: *No explicit evidence of non-academic stakeholder involvement, engagement, or influence.*

15. Kim, Koniak-Griffin et al. 2004

Aim & Target population: *Cardovascular health promotion among latino immigrants*

Inception: *Collaboration with local department of health and nurses*
Design: *Local department of health refined area for targeting; 2 community based organizations and the formation of an advisory board (including migrants) was instrumental in tailoring the study to the target community (e.g. changing name from lay health advisor to promotoras de salud).*
Implementation: *Lay health advisors were recruited and trained, by the local department of health, to deliver the intervention*
Analysis: *No explicit evidence of non-academic stakeholder involvement, engagement, or influence.*
Evaluation: *Feedback from the local health department, community advisory boards and lay health educators allowed flexible iteration of the intervention*
Dissemination: *No explicit evidence of non-academic stakeholder involvement, engagement, or influence.*

16. Lam, McPhee et al. 2003

Aim & Target population: *Increase pap/cervical cancer screening among Vietnamese-american women*

Inception: *No explicit evidence of non-academic stakeholder involvement, engagement, or influence.*
Design: *Shared with the REACH (community) coalition (who had overall power); community forum was held with 200 vietnamese-american women and their families to discuss cervical cancer and brainstorm strategies to increase screening (the REACH coalition then took findings to generate the intervention); The results were shared with a second community forum, with feedback used to finalise the intervention (by the coalition).*
Implementation: *The lay health worker campaign was community-led; Lay health educators were the target population; the community coalition was involved in implementing a media campaign.*
Analysis: *No explicit evidence of non-academic stakeholder involvement, engagement, or influence; institutional review board deemed partners could not collect data.*
Evaluation: *Media campaign was evaluated at a community forum in which community members opinions were sought through questionnaires and discussions. The REACH coalition retained the power to effect changes; lay health worker opinion was solicited through research methods/questionnaires; No evidence of individual health workers being able to influence intervention in progress.*
Dissemination: *No explicit evidence of non-academic stakeholder involvement, engagement, or influence.*

17. Li, Yeh et al. 2019

Aim & Target population: *Colorectal cancer prevention among Chinese-americans*

Inception: *Researchers and leaders from community groups agreed to promote colorectal cancer prevention in Chinese-americans.*
Design: *Designed between the researchers with feedback from the community group; Researcher carried out a literature review with feedback from community partner, research with the actual community in the form of qualitative interviews was conducted to inform the design; Appears community group did have influence, changed from a web-based idea due to community group input.*
Implementation: *Delivered by the researchers*
Analysis: *No explicit evidence of non-academic stakeholder involvement, engagement, or influence.*
Evaluation: *The community group members were involved in filling out surveys as part of the evaluation*
Dissemination: *No explicit evidence of non-academic stakeholder involvement, engagement, or influence.*

18. Nilvarangkul, McCann et al. 2011

Aim & Target population: *Enhancing health-related quality of life for Laotian migrants*

Inception: *Conceived by researchers with a broad action plan developed in collaboration with the target population*
Design: *The target population were involved in interviews used to shape the intervention; meetings were held with community workers and stakeholders to create action plans*
Implementation: *Community workers, stakeholders and researchers implemented the action plan*
Analysis: *No explicit evidence of non-academic stakeholder involvement, engagement, or influence.*
Evaluation: *Meetings were held with community workers and stakeholders as part of the formative evaluation; intervention participants were followed up via qualitative data collection*
Dissemination: *No explicit evidence of non-academic stakeholder involvement, engagement, or influence.*

19. Pinsker, Call et al. 2017

Aim & Target population: *Tobacco use prevention videos for Somali youth*

For peer review only

Inception: *No explicit evidence of non-academic stakeholder involvement, engagement, or influence.*
Design: *There was indirect involvement through focus groups of the target population; there was direct involvement of the target population in developing the videos, building upon the focus groups.*
Implementation: *Target population were involved in shaping the implementation of the intervention*
Analysis: *No explicit evidence of non-academic stakeholder involvement, engagement, or influence.*
Evaluation: *Target population evaluated the intervention through participant interviews*
Dissemination: *No explicit evidence of non-academic stakeholder involvement, engagement, or influence.*

20. Quandt, Grzywacz et al. 2013

Aim & Target population: *Pesticide safety intervention for migrant farmworkers*

Inception: *Based on an existing relationship between the university and community partners*
Design: *Based on an existing partnership; not immediately obvious how taget population and non-academic stakeholders were involved.*
Implementation: *Implemented by the community partners; lay health promoters were recruited from within the community and trained by the community partner*
Analysis: *No explicit evidence of non-academic stakeholder involvement, engagement, or influence.*
Evaluation: *No explicit evidence of non-academic stakeholder involvement, engagement, or influence.*
Dissemination: *No explicit evidence of non-academic stakeholder involvement, engagement, or influence.*

21. Solorio, Norton-Shelpuk et al. 2014

Aim & Target population: *HIV prevention messages for young Latino immigrant men who have sex with men*

Inception: *There is a previous community-university relationship, uncertain as to where the inception for the project came.*
Design: *Storyboard/intervention was put before the target group in focus groups, Latino MSM were hired to facilitate the focus groups and help interpret the findings*
Implementation: *Intervention was actioned by an external digital marketing/media team who used the design focus groups to develop the campaign*
Analysis: *Latino MSM were hired to help interpret the findings*
Evaluation: *The target population were involved in focus groups to assess their comprehension of the intervention*
Dissemination: *No explicit evidence of non-academic stakeholder involvement, engagement, or influence.*

22. Song, Han et al. 2010

Aim & Target population: *Translating dietary guidelines into a culturally tailored nutrition education program for Korean-American immigrants with type 2 diabetes*

Inception: *There is a previous relationship between researchers and community, but no clear link as to how this explicitly influenced inception*
Design: *No explicit evidence of non-academic stakeholder involvement, engagement, or influence.*
Implementation: *No explicit evidence of non-academic stakeholder involvement, engagement, or influence; appears the education program was delivered by nurses and dieticians*
Analysis: *No explicit evidence of non-academic stakeholder involvement, engagement, or influence.*

For peer review only

Evaluation: *Target population were involved in evaluating the interventions' acceptability*
Dissemination: *No explicit evidence of non-academic stakeholder involvement, engagement, or influence.*

23. Suarez-Balcazar, Early et al. 2018

Aim & Target population: *Healthy lifestyle promotion among Latino immigrant families with youth with disabilities*

Inception: *No explicit evidence of non-academic stakeholder involvement, engagement, or influence.*
Design: *Focus groups with the target population; interviews and conversations with community stakeholders and agency personnel; research built on by collaboration with mothers and staff from community organisations*
Implementation: *Delivered by bilingual occupational therapy and nutrition students; co-facilitated by mothers who presented Zumba sessions and prepared healthy snacks.*
Analysis: *No explicit evidence of non-academic stakeholder involvement, engagement, or influence.*
Evaluation: *Evaluated through focus groups at the end of the intervention*
Dissemination: *No explicit evidence of non-academic stakeholder involvement, engagement, or influence.*

24. Vaughn & Jacquez. 2019

Aim & Target population: *Stress and coping intervention for Latino immigrants*

Inception: *There are existing relations between the researchers and community; migrant community members were selected to be co-researcher*
Design: *Design input came from researchers and community co-researchers*
Implementation: *Community co-researchers implemented the intervention*
Analysis:
Evaluation: *Co-researchers provided evaluation through interviews with the researchers*
Dissemination: *No clear evidence, though does mention co-researchers want to be involved in dissemination including publications and presentations*

25. Wieland, Nigon. 2019

Aim & Target population: *Tuberculosis screening for migrant adults*

Inception: *Conceived and initiated by a community organisation*
Design: *Academic and non-academic community organisation co-designed the intervention; not sure of the involvement of the target population*
Implementation: *Intervention was implemented by community organisation staff, along with clinical/nursing staff and students*
Analysis: *Evidence was collected and analysed by local health practitioners*
Evaluation: *No explicit evidence of non-academic stakeholder involvement, engagement, or influence.*
Dissemination: *No explicit evidence of non-academic stakeholder involvement, engagement, or influence.*

26. Wieland, Njeru et al. 2017

Aim & Target population: *Digital storytelling intervention for immigrants and refugees with diabetes*

Inception: *Impetus came from an existing researcher-community partnership; diabetes co-agreed as an area for research and intervention*
Design: *survey developed by community-academic partnership to understand target population, attitudes and behaviours in T2DM; focus groups were conducted to further inform intervention*

For peer review only

	content and to identify gifted storytellers; storytellers were recruited by community partners and their stories were captured, recorded and edited to derive the final intervention product.<br>***Implementation:*** *No explicit evidence of non-academic stakeholder involvement, engagement, or influence.*<br>***Analysis:*** *No explicit evidence of non-academic stakeholder involvement, engagement, or influence.*<br>***Evaluation:*** *participants completed follow-up surveys*<br>***Dissemination:*** *No explicit evidence of non-academic stakeholder involvement, engagement, or influence.*
**27.**	**Wieland, Wies. 2012**<br><br>***Aim & Target population:*** **Physical activity and nutrition program for immigrant and refugee women**<br><br>***Inception:*** *Conceived from a community-academic partnership with long-standing ties; formalised to use CBPR to improve migrant health*<br>***Design:*** *Exercise programme piloted; refugee and immigrant women were included as focus group participants*<br>***Implementation:*** *Implemented by researchers and community organisation; community organisation recruited women to the programme; community organisation provided facilities*<br>***Analysis:*** *No explicit evidence of non-academic stakeholder involvement, engagement, or influence.*<br>***Evaluation:*** *No explicit evidence of non-academic stakeholder involvement, engagement, or influence.*<br>***Dissemination:*** *No explicit evidence of non-academic stakeholder involvement, engagement, or influence.*
**28.**	**Williams, Ochsner et al. 2010**<br><br>***Aim & Target population:*** *Health and safety training for Latino day laborers*<br><br>***Inception:*** *Inception appears to come from advocacy/community groups*<br>***Design:*** *Focus groups held with the target population to identify typical exposures and situations to be addressed by peer trainers; curriculum was revised with feedback from these peer trainers*<br>***Implementation:*** *Delivered by peer-trainers*<br>***Analysis:*** *No explicit evidence of non-academic stakeholder involvement, engagement, or influence.*<br>***Evaluation:*** *Evaluated through focus group discussions and pre & post-training surveys;*<br>***Dissemination:*** *No explicit evidence of non-academic stakeholder involvement, engagement, or influence.*

For peer review only

PRISMA 2020 Checklist

Section and Topic	Item #	Checklist item	Location where item is reported
**TITLE**			
Title	1	Identify the report as a systematic review.	Title (pg.1); Abstract (pg.2)
**ABSTRACT**			
Abstract	2	See the PRISMA 2020 for Abstracts checklist.	Abstract (pg. 2-3)
**INTRODUCTION**			
Rationale	3	Describe the rationale for the review in the context of existing knowledge.	Introduction (pg.5)
Objectives	4	Provide an explicit statement of the objective(s) or question(s) the review addresses.	Introduction (pg.5-7)
**METHODS**			
Eligibility criteria	5	Specify the inclusion and exclusion criteria for the review and how studies were grouped for the syntheses.	Pg.7
Information sources	6	Specify all databases, registers, websites, organisations, reference lists and other sources searched or consulted to identify studies. Specify the date when each source was last searched or consulted.	Pg.8
Search strategy	7	Present the full search strategies for all databases, registers and websites, including any filters and limits used.	Additional file 1.
Selection process	8	Specify the methods used to decide whether a study met the inclusion criteria of the review, including how many reviewers screened each record and each report retrieved, whether they worked independently, and if applicable, details of automation tools used in the process.	Pg.8-9
Data collection process	9	Specify the methods used to collect data from reports, including how many reviewers collected data from each report, whether they worked independently, any processes for obtaining or confirming data from study investigators, and if applicable, details of automation tools used in the process.	Pg.9
Data items	10a	List and define all outcomes for which data were sought. Specify whether all results that were compatible with each outcome domain in each study were sought (e.g. for all measures, time points, analyses), and if not, the methods used to decide which results to collect.	Pg. 9-11
	10b	List and define all other variables for which data were sought (e.g. participant and intervention characteristics, funding sources). Describe any assumptions made about any missing or unclear information.	Pg. 9-11
Study risk of bias assessment	11	Specify the methods used to assess risk of bias in the included studies, including details of the tool(s) used, how many reviewers assessed each study and whether they worked independently, and if applicable, details of automation tools used in the process.	n/a – well recognised and validated tools are not available for participatory research
Effect measures	12	Specify for each outcome the effect measure(s) (e.g. risk ratio, mean difference) used in the synthesis or presentation of results.	n/a – did not include numerical outcomes such as risk ratio or mean

For peer review only - http://bmjopen.bmj.com/site/about/guidelines.xhtml

PRISMA 2020 Checklist

Section and Topic	Item #	Checklist item	Location where item is reported
			difference
Synthesis methods	13a	Describe the processes used to decide which studies were eligible for each synthesis (e.g. tabulating the study intervention characteristics and comparing against the planned groups for each synthesis (item #5)).	Pg. 9-11
	13b	Describe any methods required to prepare the data for presentation or synthesis, such as handling of missing summary statistics, or data conversions.	Pg. 9-11
	13c	Describe any methods used to tabulate or visually display results of individual studies and syntheses.	Pg. 9-11
	13d	Describe any methods used to synthesize results and provide a rationale for the choice(s). If meta-analysis was performed, describe the model(s), method(s) to identify the presence and extent of statistical heterogeneity, and software package(s) used.	Pg. 9-11
	13e	Describe any methods used to explore possible causes of heterogeneity among study results (e.g. subgroup analysis, meta-regression).	n/a – we did not explore heterogeneity
	13f	Describe any sensitivity analyses conducted to assess robustness of the synthesized results.	n/a – we did not perform sensitivity analyses as we did not include numerical outcomes that would be suitable
Reporting bias assessment	14	Describe any methods used to assess risk of bias due to missing results in a synthesis (arising from reporting biases).	n/a – we have no formal reporting bias assessment; we do discuss reporting issues in the discussion
Certainty assessment	15	Describe any methods used to assess certainty (or confidence) in the body of evidence for an outcome.	n/a – there are no definitive methods for assessing certainty around the data we utilise. We do discuss issues

For peer review only

PRISMA 2020 Checklist

Section and Topic	Item #	Checklist item	Location where item is reported
			around variability in terms, application of methods and lack of reporting.
**RESULTS**			
Study selection	16a	Describe the results of the search and selection process, from the number of records identified in the search to the number of studies included in the review, ideally using a flow diagram.	Figure 1 (pg.15); pg.14
	16b	Cite studies that might appear to meet the inclusion criteria, but which were excluded, and explain why they were excluded.	Figure 1 details reasons for exclusion.
Study characteristics	17	Cite each included study and present its characteristics.	Table 3 (pg.16-19)
Risk of bias in studies	18	Present assessments of risk of bias for each included study.	n/a - there are no definitive methods for assessing certainty around the data we utilise. We do discuss issues around variability in terms, application of methods and lack of reporting.
Results of individual studies	19	For all outcomes, present, for each study: (a) summary statistics for each group (where appropriate) and (b) an effect estimate and its precision (e.g. confidence/credible interval), ideally using structured tables or plots.	Table 2 & table 4.<br>There are no numerical outcomes to report or synthesise
Results of	20a	For each synthesis, briefly summarise the characteristics and risk of bias among contributing studies.	Pg. 19-23

For peer review only - http://bmjopen.bmj.com/site/about/guidelines.xhtml

PRISMA 2020 Checklist

Section and Topic	Item #	Checklist item	Location where item is reported
syntheses	20b	Present results of all statistical syntheses conducted. If meta-analysis was done, present for each the summary estimate and its precision (e.g. confidence/credible interval) and measures of statistical heterogeneity. If comparing groups, describe the direction of the effect.	n/a
	20c	Present results of all investigations of possible causes of heterogeneity among study results.	n/a – we did not examine heterogeneity
	20d	Present results of all sensitivity analyses conducted to assess the robustness of the synthesized results.	n/a – there were no numerical outcomes on which to perform sensitivity analysis
Reporting biases	21	Present assessments of risk of bias due to missing results (arising from reporting biases) for each synthesis assessed.	n/a - we have no formal reporting bias assessment; we do discuss reporting issues in the discussion
Certainty of evidence	22	Present assessments of certainty (or confidence) in the body of evidence for each outcome assessed.	n/a – there are no definitive methods for assessing certainty around the data we utilise. We do discuss issues around variability in terms, application of methods and lack of reporting.
**DISCUSSION**			
Discussion	23a	Provide a general interpretation of the results in the context of other evidence.	Pg. 26-31
	23b	Discuss any limitations of the evidence included in the review.	Pg. 31-32

For peer review only - http://bmjopen.bmj.com/site/about/guidelines.xhtml

PRISMA 2020 Checklist

Section and Topic	Item #	Checklist item	Location where item is reported
	23c	Discuss any limitations of the review processes used.	Pg. 31-32
	23d	Discuss implications of the results for practice, policy, and future research.	Pg. 30-31; 33 (conclusion)
**OTHER INFORMATION**			
Registration and protocol	24a	Provide registration information for the review, including register name and registration number, or state that the review was not registered.	Pg. 3 (abstract); pg. 7
	24b	Indicate where the review protocol can be accessed, or state that a protocol was not prepared.	Pg. 3 (abstract); pg. 7
	24c	Describe and explain any amendments to information provided at registration or in the protocol.	n/a
Support	25	Describe sources of financial or non-financial support for the review, and the role of the funders or sponsors in the review.	Pg. 34
Competing interests	26	Declare any competing interests of review authors.	Pg. 34
Availability of data, code and other materials	27	Report which of the following are publicly available and where they can be found: template data collection forms; data extracted from included studies; data used for all analyses; analytic code; any other materials used in the review.	Pg. 34

From: Page MJ, McKenzie JE, Bossuyt PM, Boutron I, Hoffmann TC, Mulrow CD, et al. The PRISMA 2020 statement: an updated guideline for reporting systematic reviews. BMJ 2021;372:n71. doi: 10.1136/bmj.n71

For more information, visit: http://www.prisma-statement.org/

For peer review only
